# Polycyclic nitrogen heterocycles as potential thymidine phosphorylase inhibitors: synthesis, biological evaluation, and molecular docking study

**DOI:** 10.1080/14756366.2021.2001806

**Published:** 2021-12-22

**Authors:** Karen Aknin, Alexis Bontemps, Amaury Farce, Eric Merlet, Philippe Belmont, Philippe Helissey, Philippe Chavatte, Marie-Agnès Sari, Sylviane Giorgi-Renault, Stéphanie Desbène-Finck

**Affiliations:** a Faculté de Santé, Faculté de Pharmacie de Paris, Cibles Thérapeutiques et Conception de Médicaments (CiTCoM), CNRS UMR8038, Université de Paris, Paris, France; b Inserm, CHU Lille, U1286 – INFINITE – Institute for Translational Research in Inflammation, Université de Lille, Lille, France; c Faculté des Sciences, CNRS, UMR 8601, Laboratoire de Chimie et Biochimie Pharmacologiques et Toxicologiques, Université de Paris, Paris, France

**Keywords:** Thymidine phosphorylase inhibitor, multicomponent reactions, molecular docking, pyrido[2,3-*d*]pyrimidinedione, pyrimido[4,5-*b*]quinoline-2,4-dione

## Abstract

New polycyclic heterocycles were synthesised and evaluated as potential inhibitors of thymidine phosphorylase (TP). Inspired by the pharmacophoric pyrimidinedione core of the natural substrate, four series have been designed in order to interact with large empty pockets of the active site: pyrimidoquinoline-2,4-diones (series A), pyrimidinedione linked to a pyrroloquinoline-1,3-diones (series B and C), the polycyclic heterocycle has been replaced by a pyrimidopyridopyrrolidinetetraone (series D). In each series, the tricyclic nitrogen heterocyclic moiety has been synthesised by a one-pot multicomponent reaction. Compared to **7-DX** used as control, **2d**, **2l**, **2p** (series A), **28a** (series D), and the open intermediate **30** showed modest to good activities. A kinetic study confirmed that the most active compounds **2d**, **2p** are competitive inhibitors. Molecular docking analysis confirmed the interaction of these new compounds at the active binding site of TP and highlighted a plausible specific interaction in a pocket that had not yet been explored.

## 1. Introduction

Angiogenesis – the formation of new blood vessels from pre-existing vasculature – has been validated as a target for several tumours and has been shown to promote tumour growth and metastasis^1^. Given the complexity of this process, there is a need for new compounds targeting multiple pro-angiogenic factors^2–5^. In this context, thymidine phosphorylase (TP) seems to be an interesting and understudied therapeutic target.

**Figure 1.**
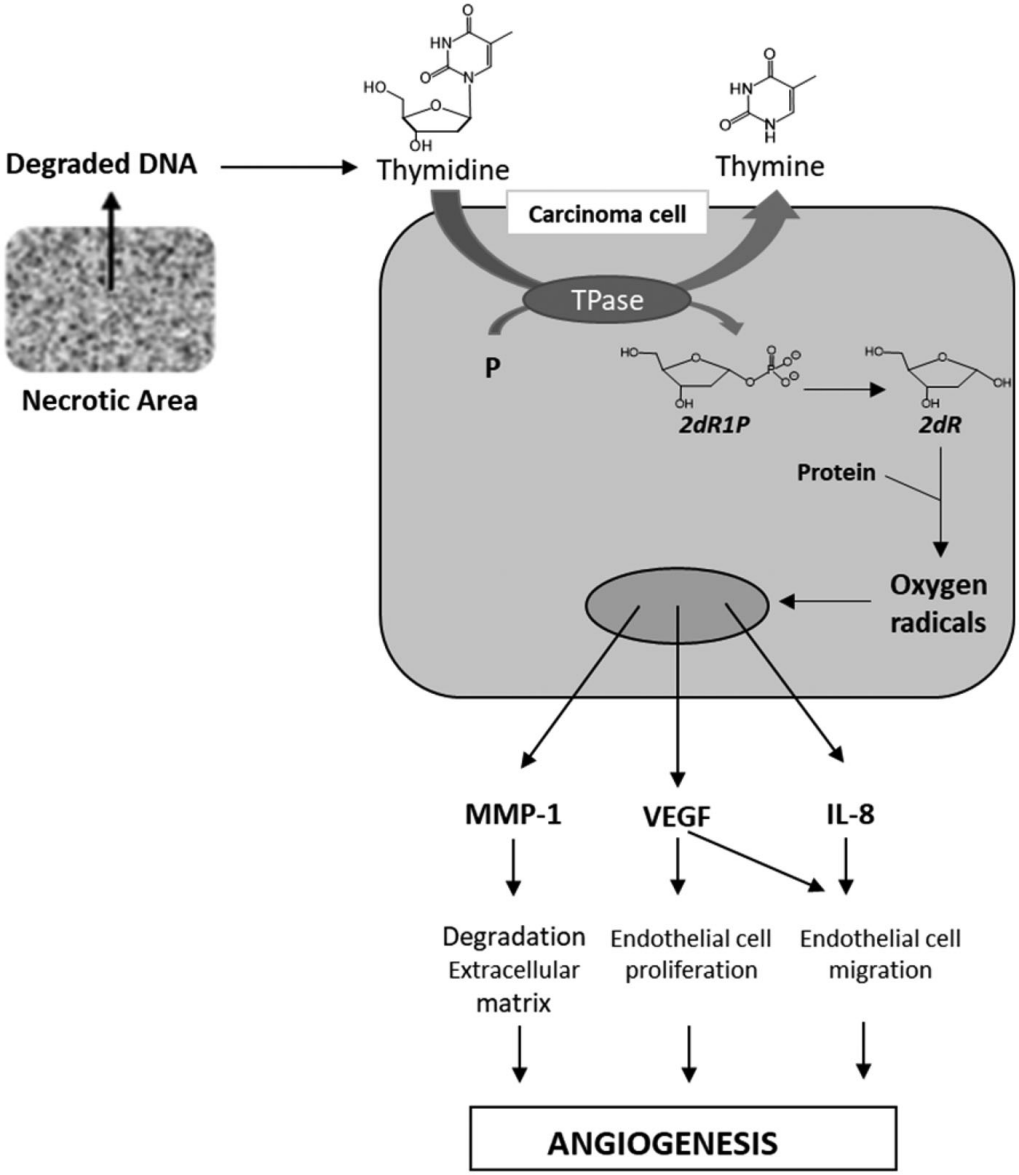
TP and 2dR1P roles in tumour development.

The physiological role of TP is to catalyse the reversible phosphorolysis of thymidine into thymine and 2-deoxyribose-1-phosphate (2dR1P) that is metabolised into 2-deoxyribose (2dR) (Figure 1). TP has been shown to be up-regulated in the hypoxic regions of many solid tumours (stomach, pancreas, kidney, oesophagus, breast, ovary, lung, colon, bladder, uterus, kidney …)^4^^,^^6^^,^^7^. Usually, this over-expression is highly associated with tumour micro vessel level, infiltration, metastasis and also correlates with the aggressiveness and invasiveness of the cancer^6^. Via 2dR, TP stimulates the secretion of several pro-angiogenic factors such as VEGF, MMP-1, IL-8 by both malignant cells and stromal cells located in the tumour microenvironment^8^^,^^9^. Consequently, TP inhibitors seem to be promising agents^10^.

**Figure 2.**
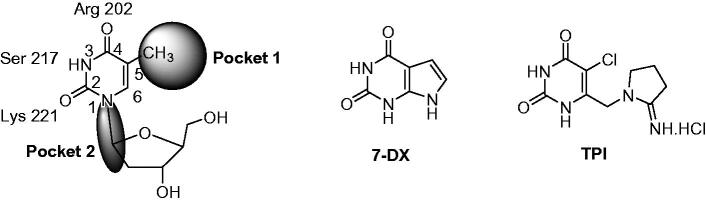
Schematic representation of the pyridimidinedione binding site of hTP and chemical structures of **7-DX** and **TPI**.

Various crystal structures of *Escherichia coli* TP and human TP (hTP) complexes with thymine or thymidine analogues have been published^11–16^. Briefly, the binding of thymidine is ensured by three hydrogen bonds between the two carbonyls and the NH function with an arginine, a serine and a lysine. These studies revealed a large empty space (pocket 1) facing the C-5 and C-6 of the pyrimidinedione and another (pocket 2) close to the 1-position (Figure 2).

Various TP inhibitors have been reported in the literature^8^^,^^17^^,^^18^. For a recent review see Sajid et al.^19^. In order to interact with the thymidine binding site, most of them are pyrimidine-2,4-dione derivatives. 7-Deazaxanthine (**7-DX**) and 5-chloro-6-[1-(2-iminopyrrolidinyl)methyl]uracil hydrochloride (**TPI**) have emerged (Figure 2)^20^^,^^21^. Up to now, the most potent inhibitor is **TPI** (IC_50 hTP_ = 0.035 µM^8^), whose anti-angiogenic activity was attested through several *in vitro* and *in vivo* models^22–24^. **TPI** has been co-crystallized with hTP^12^. In combination with trifluridine, **TPI** has been recently approved (TAS-102, Lonsurf^®^) for the treatment of metastatic colorectal cancer^25^. The role of **TPI** is dual, it prevents the degradation of trifluridine by TP and exerts anti-angiogenic effect.

Only a few polycyclic TP inhibitors have been reported (for a review see Bera et al.^8^). Crystallographic studies data allowed us to design inhibitors in which the natural ligand feature is linked to a polycyclic aromatic nitrogen heterocycle either by ring annelation or via various linkers.

In the present study, we describe the synthesis and the biological *in vitro* evaluation as TP inhibitors of four series of new original aromatic derivatives. To elucidate the mechanism of enzyme inhibition of the hits, a brief kinetic study was attempted. *In silico* molecular docking studies have also been performed to explore the binding site and possible interactions mode of these new derivatives with TP.

## 2. Results and discussion

### 2.1. Chemistry

**Figure 3.**
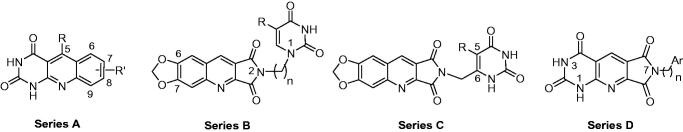
General structures of the designed derivatives in series A–D.

In a first time, we have designed three series of new heterocycles as potential TP inhibitors. The general structures are presented in Figure 3. In series A, the pyrimidinedione was annelated to a quinoline giving a rigid tricyclic heterocycle. The substituents on the 5 to 9-positions could modulate the interaction with pocket 1. Compounds of series B and C were more flexible. Thus, a pyrimidinedione substituted by a methylenic chain either on the 1-position (series B) or on the 6-position (series C) was linked to the 2-position of a quinolopyrrolidinedione. In a second time, the results of a first docking study allowed us to design compounds of series D in which the quinolopyrrolidinedione has been replaced by a pyrimidopyridopyrrolidinetetraone.

Our strategies for synthesising the tricyclic heterocycles are based on one-pot multicomponent reactions, developed in our group, that involved an aniline, an aldehyde, and a 1,3-dicarbonyl derivative^26–28^.

#### 2.1.1. Pyrimido[4,5-b]quinoline-2,4(1H, 3H)-diones (series A)

We have described a very simple and convenient one-pot reaction for the synthesis of pyrimido[4,5-*b*]quinoline-2,4(*1H*,3*H*)-diones involving an aldehyde, an aniline, and barbituric acid **1** as the 1,3-dicarbonyl reagent (Table 1)^27^. This straightforward method circumvents the preparation of unstable substituted 2-aminobenzaldehydes that limits the scope of previously described syntheses^29–31^. The use of commercially available anilines allowed the facile syntheses of pyrimido[4,5-*b*]quinolinediones substituted on the 6 to 9-positions with electron donor or electron-withdrawing groups. Access to the 5-substituted derivatives is also possible starting from aliphatic or aromatic aldehydes. We have previously described the synthesis of compounds **2a–d**, **2f–h**, **2j–k**, **2m**, **2p–r** which have been obtained in 25% to 88% yield^27^. According to this method, six new derivatives (**2e**, **2i**, **2l**, **2n**, **2o, 2s**) have been synthesised in modest-to-good yields (Table 1).

**Table 1.** Synthesis of the pyrimido[4,5*-b*]quinoline-2,4(*1H,3H*)-diones **2**. 
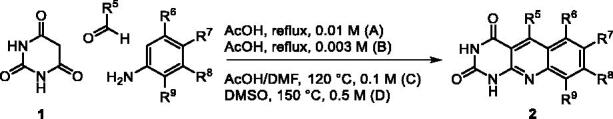

Compound	R^5^	R^6^	R^7^	R^8^	R^9^	Method	Reaction time (h)	Yield (%)
**2a** ^a^	H	H	CH_3_	H	H	D	72	45**^b^**
**2b** ^a^	H	H	H	CH_3_	H	C	2	56**^b^**
**2c** ^a^	H	H	H	H	CH_3_	A	1^e^	25**^d^**
**2d** ^a^	H	H	H	OCH_3_	H	C	2	70**^b^**
**2e**	H	H	H	OPh	H	C	6	57^d^
**2f** ^a^	H	H	H	OCH_2_Ph	H	C	8	66**^b^**
**2g** ^a^	H	H	O–CH_2_–O		H	A	19	75**^b^**
**2h** ^a^	H	OCH_3_	OCH_3_	OCH_3_	H	A	2	73**^b^**
**2i**	H	H	H	CH_2_Ph	H	C	3	41^d^
**2j** ^a^	H	H	H	Cl	H	A	100	50**^b^**
**2k** ^a^	H	H	H	CF_3_	H	A	1^e^	51**^b^**
**2l**	H	H	Cl	Cl	H	A	5	17^b^
**2m** ^a^	H	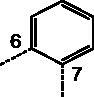		H	H	A	48	65**^b^**
**2n**	H	H	H	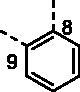		A	20	32^b^
**2o**	H	H	H	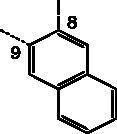		B	3	67^d^
**2p** ^a^	CH_3_	H	H	OCH_3_	H	A	24	77**^b^**
**2q** ^a^	CH_3_	H	O-CH_2_-O		H	A	4.5	44**^b^**
**2r** ^a^	Ar^c^	H	O-CH_2_-O		H	A	67	88**^b^**

^a^Previously described compound^27^. ^b^Yield of pure crude product. ^c^Ar = 3,4,5-trimethoxyphenyl. ^d^Yield of purified product. ^e^Incomplete reaction.

It is worth noting that the presence of two electron-withdrawing groups on anilines is allowed but provided compound **2l** in low yield (17%). Tetra and pentacyclic derivatives have been obtained respectively using 1-naphthylamine and 1-aminoanthracene (compounds **2n** and **2o**). In the last case, it was necessary to dilute the reaction mixture (330 mL of AcOH/mmol instead of 100 mL) to avoid the precipitation of the poorly soluble dihydro intermediate. The angular structures of compounds **2n**, and **2o** have been unambiguously determined by NMR.

**Scheme 1.**
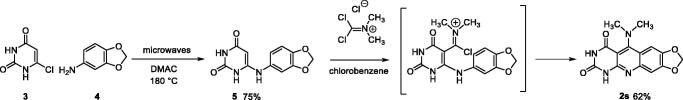
Synthesis of compound **2s**.

The preparation of compound **2s** has required another reaction scheme. In the 1,3-dimethyl series, the literature described the synthesis of 5-dimethylaminopyrimidoquinolines using 6-arylamino-1,3-dimethyluracils as starting materials. When the synthesis was performed by nucleophilic substitution on an intermediate 5-thiomethyl heterocycle, the overall yields were very low due to the preparation of the thiomethylheterocycle^32^. The use of phosgene iminium chloride (Viehe’s salt) furnished pyrimidoquinolines substituted either on the 5-position by a 5-dimethylamino group (in the presence of triethylamine) or by a chloride (without triethylamine)^33^. The dimethylpyrimidoquinoline **2s** has been prepared using this second procedure, so 6-arylaminouracil **5** was obtained by reaction of 6-chlorouracil **3** with 3,4-methylenedioxyaniline **4**, under microwave irradiation, according to the method described by Fang et al.^34^. Then, the reaction of the Viehe’s salt on **5** gave an iminium intermediate that cyclized into **2s** (Scheme 1). Surprisingly, the reaction leads to the dimethylamino compound even in the absence of triethylamine. The low solubility of **2s**, which precipitates in the reaction mixture, probably prevents the nucleophilic attack by chloride ions observed in other series.

#### 2.1.2. 2H-pyrroloquinoline-1,3-diones/pyrimidinediones (series B and C)

Compounds of series B et C have been obtained by N^2^-alkylation of the 6,7-methylenedioxy-2*H*-pyrrolo[3,4-*b*]quinolines (**10**) using a pyrimidinedione substituted by a halogenoalkyl chain.

Although many publications have already reported the synthesis of N^2^-substituted *2H*-pyrrolo[3,4-*b*]quinolines, only three have been covered regarding the N^2^-unsubstituted heterocycle. The reported methods are multistep syntheses. Starting from the corresponding 2-aminoacetophenones, the 9-methyl derivatives were obtained as a guanidinium salt in two steps and 38% overall yield^35^ and the 9-phenyl analogue in 5 steps and 12% overall yield^36^. The ultimate step of the third chemical scheme involved the condensation of urea on the quinoline-2,3-dicarboxylic diacid to give the unsubstituted heterocycle^37^.

In a first time, we intended to apply this last method to the synthesis of the 6,7-methylenedioxy derivative **10** using a 6,7-methylenedioxyquinoline-2,3-dicarboxylate. In the literature, an analogous dimethyl ester has been already described either by reaction of the 2-aminobenzaldehyde with dimethyl acetylenedicarboxylate and subsequent acidic treatment^38^ or by reaction of the methylenedioxyaniline with the dimethyl acetylenedicarboxylate and subsequent treatment with the Vilsmeier reagent^39^. In both cases, the yield of the second step was not specified.

**Scheme 2.**
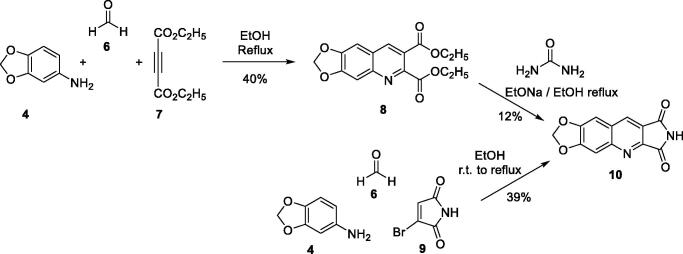
Synthesis of compound **10**.

We thought that it would be possible to prepare the diethyl ester **8** in a one-pot reaction. Indeed, **8** was obtained in 40% yield by refluxing in EtOH a mixture of aniline **4**, diethyl acetylenedicarboxylate (**7**) and formaldehyde (**6**) (Scheme 2). Unfortunately, the condensation of **8** with urea afforded **10** in only 12% yield. Assays with the corresponding diacid were unsuccessful.

Finally, we have synthesised **10** in 39% yield according to an adjustment of our three-component one-pot method replacing the cyclic 1,3-diketone by bromomaleimide (**9**) which is more easily accessible than the hydroxymaleimide (Scheme 2)^28^. With this example, we have shown that the scope of the one-pot reaction could be extended to 1,3-diketone analogues such as a cyclic β-halogenoketone.

**Scheme 3.**
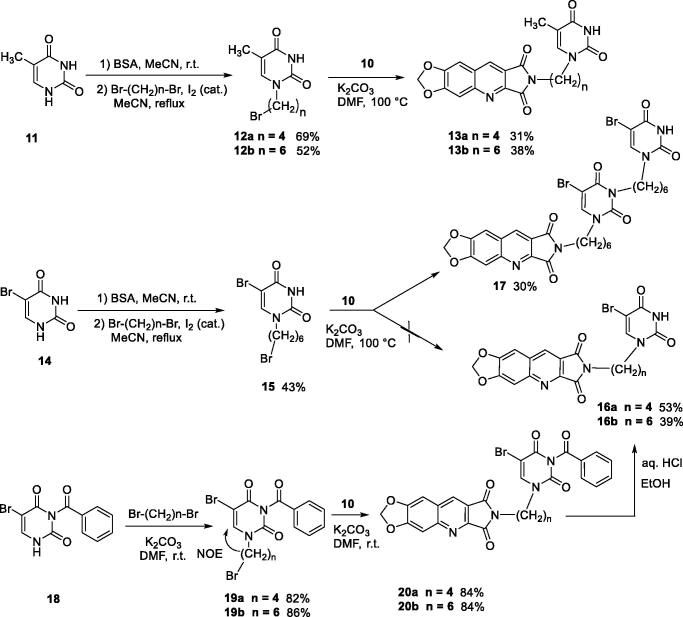
Synthesis of compounds **13a**, **13b**, **16a**, **16b**, and **17**.

It is well known that pyrimidinediones could be alkylated on the N^1^ and/or on the N^3^. As an example, the reaction of thymine with the ω-dibromobutane and the ω-dibromohexane lead to a mixture of N^1^-substituted derivatives and N^1^,N^1^′-dimers. With the dibromopentyl chain in the same conditions, only the N^1^,N^3^-disubstituted pyrimidinedione has been isolated^40^. In several papers, the N^1^-alkylation was achieved in two steps via the formation of the 2,4-O-disilyl pyrimidine in order to avoid the formation of side products^41–46^. We have prepared compounds **12a,b** and **15** according to the described procedure but without isolation of the unstable disilyl intermediates (Scheme 3).

Final compounds **13a** and **13b** were obtained by alkylation of **10** by **12a** and **12b** in DMF in the presence of K_2_CO_3_. Unfortunately, in the bromo-uracil series, it was not possible to isolate compound **16b** which reacted again with another molecule of **15** to give **17** (Scheme 3).

Access to the 5′-bromo derivatives required the use of a benzoyl protecting group on the N^3^ position of 5-bromouracil (**14**)^47^. After alkylation of the N^3^-benzoylbromouracil (**18**)^48^, the reaction of the resulting compounds **19a,b** with **10** and subsequent deprotection of the pyrimidinedione in acidic medium afforded compounds **16a** and **16b** (Scheme 3). A NOE NMR experiment realised on compound **19a** confirmed the N^1^-alkylation of the pyrimidinedione from bromouracil (**14**).

**Scheme 4.**
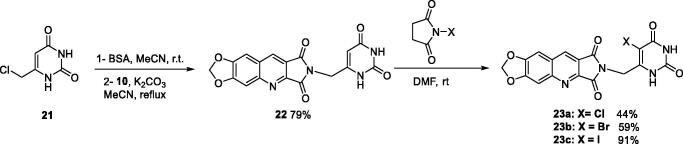
Synthesis of compounds **22** and **23a–c**.

For the synthesis of series C, it was necessary to protect the 6-chloromethyluracil (**21**) by bis(trimethylsilyl)acetamide (BSA) before the condensation with **10**. The reaction of the resulting 5-unsubstituted compound **22** with *N*-halogenosuccinimides lead to the 5′-halogeno compounds **23a–c** (Scheme 4).

Biological results and molecular modelling have prompted us to synthesise the N^2^-benzyl substituted compound **24** (Table 3) in which the pyrimidinedione moiety of derivative **22** is replaced by a phenyl nucleus. Thus, the reaction of benzyl bromide with **10** in the presence of K_2_CO_3_ in DMF furnished **24** in 65% yield.

#### 2.1.3. Pyrrolo[3′,4′:5,6]pyrido[2,3-d]pyrimidine-2,4,6,8(3H,7H)-tetraones (series D)

**Scheme 5.**
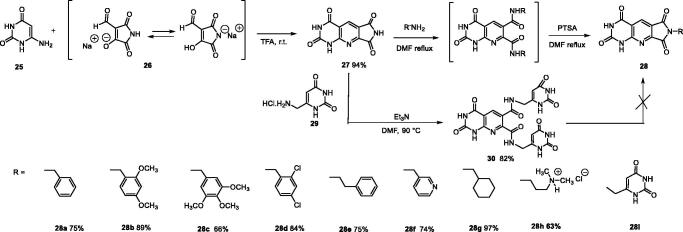
Synthesis of compounds **28a–h** and **30**.

Derivatives of series D have been synthesised from the unsubstituted tricyclic heterocycle **27** (Scheme 5). We have previously described easy access to this heterocyclic skeleton *via* a new synthon, the 4-formyl-3-hydroxy-2,5-dioxo-2,5-dihydro-1*H*-pyrrole (**26**)^28^. The functionalization of **27** was achieved by reaction with two equivalents of the appropriate amine in refluxed DMF, and cyclisation of the resulting diamide using two equivalents of *p*-toluenesulfonic acid (PTSA)^28^. Four new derivatives **28c, 28e, 28f**, and **28h** have been synthesised for the present study.

In an attempt to prepare **28i**, the reaction of the 6-aminomethyluracil hydrochloride (**29**)^49^^,^^50^ with **27** afforded only the diamide **30** which could not be cyclized into **28i** whatever the acidic conditions used (PTSA, TFA, HCl, H_3_PO_4_, B(OH)_3_) (Scheme 5).

### 2.2. In vitro thymidine phosphorylase inhibiting activity and molecular docking study

Inhibitory activities of the new heterocyclic derivatives in series A-D have been tested *in vitro* against recombinant *E. coli* TP. The adopted protocol is a modification of the original method developed by Khan^51^. TP activity and inhibition assays were based on the phosphorolysis of thymidine to thymine, this conversion results in a significant spectrophotometric decrease. At 290 nm and pH 7.4, thymidine has a higher extinction coefficient than thymine (Δ*ε* = −480 M^−1^ cm^−1^). The absorbance at 290 nm was measured every 2 min for 30 min in 96-well plates at 25 °C.

A preliminary screening has been carried out at 50 µM or at the maximum concentration allowed by the solubility of the tested compound in the incubation medium (aqueous buffer containing 2.5% of DMSO). Results are given in percentage of inhibition (average of three measurements). For the most promising compounds (inhibition > 30% and solubility > 50 µM), IC_50_ values have been determined (Tables 2–4). **7-DX**, which presents the activity of the same order of magnitude (IC_50_ *_E. coli_*_TP_ = 40 µM^8^) was used as a reference.

**Table 2.** *Escherichia coli* TP inhibition of series A derivatives.

Compound	Structure	TP inhibition (%) at 50 µM or at maximum solubility^a^ IC_50_ (µM)
**2a**	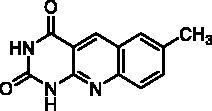	0% at 50 µM
**2b**	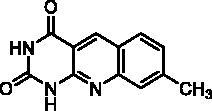	0% at 50 µM
**2c**	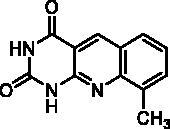	14% at 50 µM
**2d**	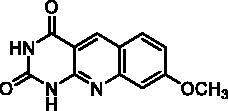	89% at 50 µM IC_50_ = 28 ± 1 µM
**2e**	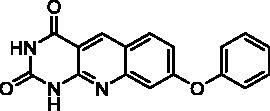	30% at 50 µM^a^
**2f**	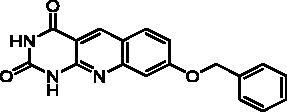	0% at 50 µM^a^
**2g**	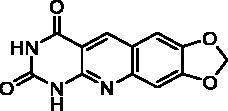	0% at 15 µM^a^
**2h**	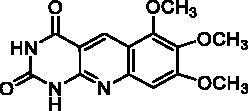	0% at 15 µM^a^
**2i**	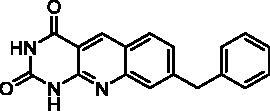	30% at 50 µM^a^
**2j**	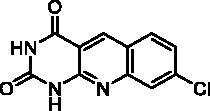	0% at 50 µM^a^
**2k**	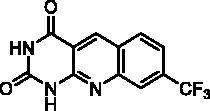	0% at 50 µM
**2l**	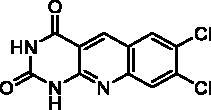	71% at 50 µM IC_50_ = 42 ± 7 µM
**2m**	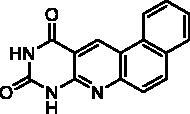	29% at 50 µM
**2n**	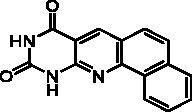	0% at 15 µM^a^
**2o**	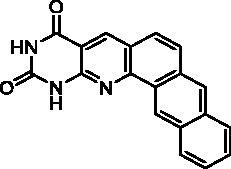	0% at 25 µM^a^
**2p**	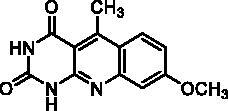	67% at 50 µM IC_50_ = 26 ± 3 µM
**2q**	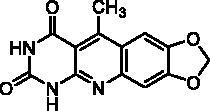	0% at 50 µM
**2r**	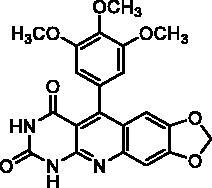	0% at 25 µM^a^
**2s**	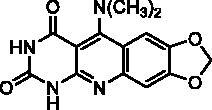	0% at 15 µM^a^
	**7-DX**	60% at 50 µM IC_50_ = 28 ± 6 µM

^a^Maximum solubility.

**Table 3.** *Escherichia coli* TP inhibition of series B and C derivatives.

Compound	Structure	TP inhibition (%) at 50 µM or maximum solubility^a^	
**13a**	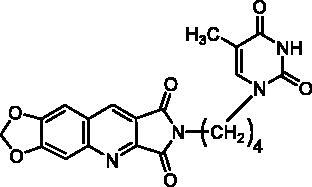	25% at 50 µM	
**13b**	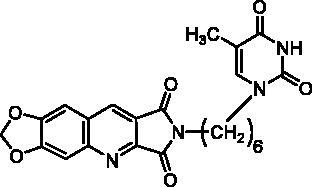	0% at 50 µM	
**16a**	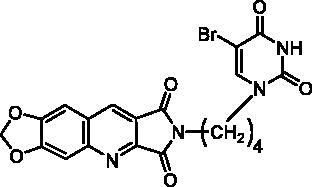	30% at 25 µM^a^	
**16b**	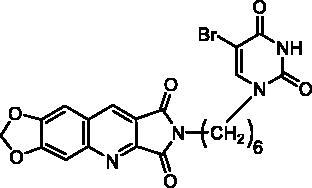	30% at 12.5 µM^a^	
**22**	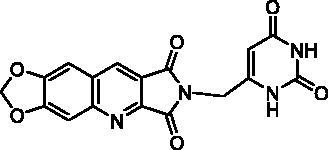	25% at 15 µM^a^	
**23a**	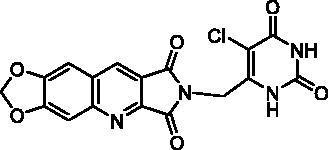	0% at 50 µM	
**23b**	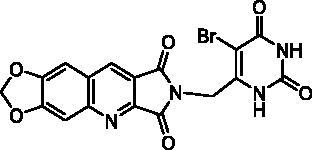	30% at 50 µM^a^	
**23c**	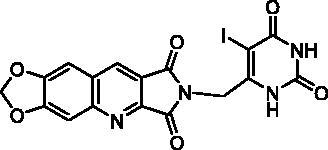	0% at 50 µM	
**24**	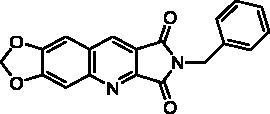	20% at 50 µM	
	**7-DX**	60% at 50 µM IC_50_ = 28 ± 6 µM	

^a^Maximum solubility.

**Table 4.** *Escherichia coli* TP inhibition of series D derivatives.

Compound	Structure	TP inhibition (%) at 50 µM or maximum solubility^a^
**28a**	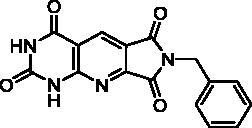	44% at 50 µM IC_50_ = 62 ± 7 µM
**28b**	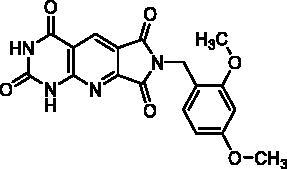	0% at 50 µM
**28c**	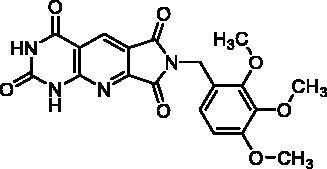	0% at 50 µM
**28d**	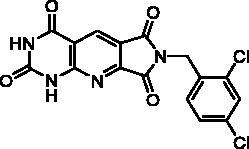	0% at 30µM^a^
**28e**	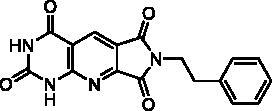	0% at 50 µM
**28f**	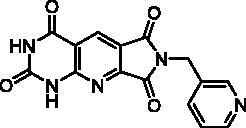	0% at 50 µM
**28g**	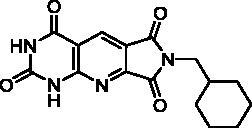	0% at 30 µM^a^
**28h**	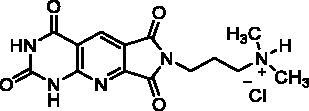	0% at 50 µM
**27**	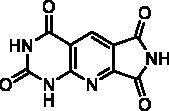	0% at 50 µM
**30**	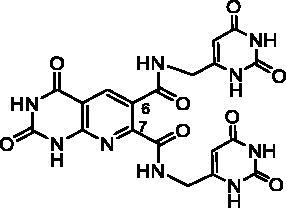	31% at 50 µM IC_50_ = 87 ± 4 µM
	**7-DX**	60% at 50 µM IC_50_ = 28 ± 6 µM

^a^Maximum solubility.

To help understand structure–activity relationships, the geometry optimised structures of the synthesised compounds were docked into the active site of hTP^12^ using GOLD 5.1. In order to validate the docking protocol, an X-ray crystal of TP complexed with **TPI** was retrieved, and the ligand was redocked into the active site of the enzyme. The predicted binding mode and interaction pattern of **TPI** generated in our study were found to be in accordance with the reported crystallographic study with an RMS deviation of 0.654 Å^12^.

Finally, a brief kinetic study was performed to elucidate the mechanism of inhibition of the two most interesting derivatives.

#### 2.2.1 Series A

Series A has been designed as a conformationally constrained series in which the pyrimidinedione moiety should interact with the thymine binding site and the other nuclei with pocket 1 facing the C-5 and C-6 of the thymine (Figure 2). In order to modulate the interactions and/or the solubility, various substituents have been introduced on the 5 to 9-positions.

Seven derivatives exhibited inhibiting activity towards *E. coli* TP (compounds **2c, 2d, 2e, 2i, 2l, 2m, 2p**), three of them (compounds **2d, 2l, 2p**) are interesting in terms of activity and solubility as compared to **7-DX** (Table 2). This preliminary enzyme inhibition study suggested the following structure-activity relationships for series A compounds:

- A methyl on the benzenic ring was unfavourable except for the 9-methyl derivative **2c** that was weakly active.
- Monosubstitution on the 8-position by an ether group seemed to be interesting in the case of a methoxy group (compound **2d**) but the activity decreased with the substituent size (compounds **2e** and **2f**). The replacement of the oxygen by methylene seemed to be possible (compare compounds **2e–2i**). Compounds **2g** and **2h** were sparingly soluble; consequently, it is not possible to conclude about the effect of the presence of two ether groups.
- The presence of an electron-withdrawing substituent on the 8-position such as a chlorine atom (compound **2j**) or a trifluoromethyl group (compound **2k**) was unfavourable. However, the 7,8-dichloro derivatives **2l** exhibited an IC_50_ in the same order of magnitude as the 8-methoxy derivative **2d**.
- The enzyme seemed to accept the presence of an additional angular benzene ring in compound **2m**. It is not possible to conclude concerning the geometrical isomer **2n** or the homologue **2o** which were less soluble.
- The introduction of methyl on the 5-position of the 8-methoxy derivative **2d** was possible (compound **2p**). In the case of compound **2g**, the 5-substituted derivatives remained inactive whatever the substituent sizes (compounds **2q, 2r, 2s**).

In this series, results were found to be optimum for the two 8-methoxypyrimido[4,5-*b*]quinoline-2,4(*1H,3H*)-diones **2d** and **2p**, and the 7,8-dichloro derivative **2l**.

**Figure 4.**
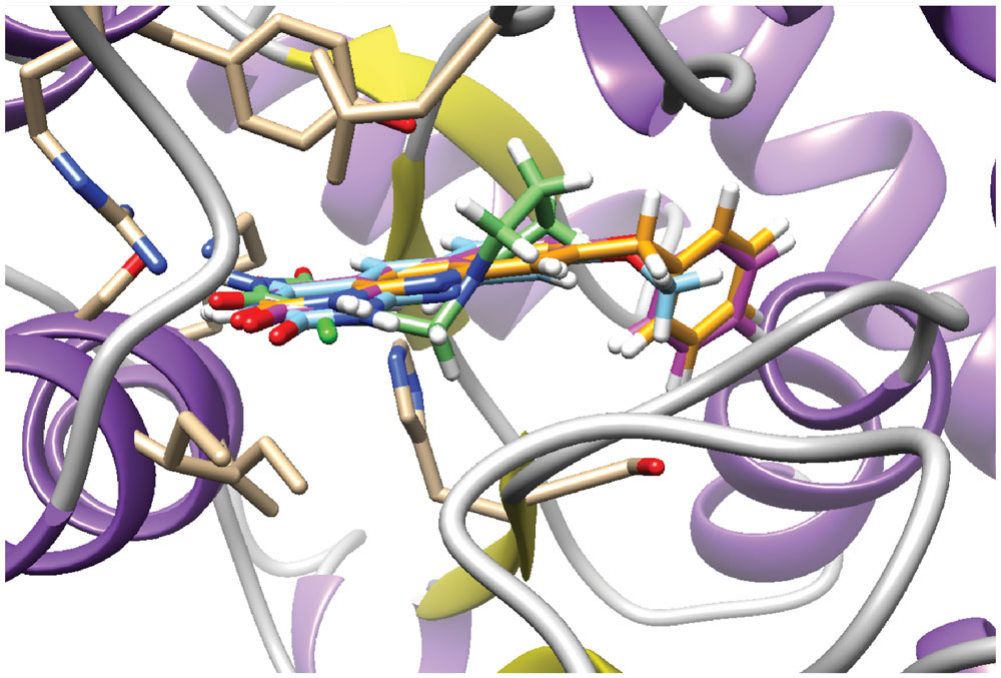
Docking superposition in the hTP active site of compounds **2d** (in blue), **2e** (in pink), **2i** (in orange), and **TPI** (in green).

As expected, preliminary docking study showed that the pyrimidinedione moiety of the series A derivatives interacted with the active site of hTP in a similar binding mode as the 5-chlorouracil fragment of **TPI**, that is, by establishing hydrogen bonds with arginine 202, serine 217 and lysine 221 of the α domain and histidine 116 of the *α*/*β* domain. As an example, docking of compound **2d** superposed on **TPI** (in green) is presented in Figure 4. Results were the same for the other active derivatives **2p** and **2l** (Supporting information, Figure S1).

The less marked activity of compounds **2c** and **2e** was also supported by the modelling results that highlighted two major binding modes corresponding in both 180° vertical and 180° horizontal rotations of the tricyclic moieties (Supporting information, Figure S2 for compound **2c** as an example). It is worth noting that several substituents on the 8-position seemed to be located in the same area of the active site as showed in Figure 4: methoxy group of compound **2d**, benzyl of compound **2i**, and phenoxy for one of the conformations of compound **2e**.

#### 2.2.2. Series B and C

Series B and C have been designed as more flexible series than series A with the aim of interactions of the pyrimidinedione in the thymidine binding site of TP and of the methylenedioxypyrroloquinoline moiety in pocket 1 (see Figure 2).

In terms of enzymatic inhibition, series B and C did not present really interesting activities in comparison with **7-DX**. The unsubstituted derivative **22** and the 5-bromopyrimidinediones **16a**, **16b**, or **23b** seemed to the more interesting but, unfortunately, they are the less soluble. The spacer size has no influence. In the case of a methylene linker (series C), the presence of a halogen on the 5-position was not favourable. More interestingly, the pyrimidinedione did not seem to be essential, it can be replaced by a phenyl nucleus (compound **24**) (Table 3).

**Figure 5.**
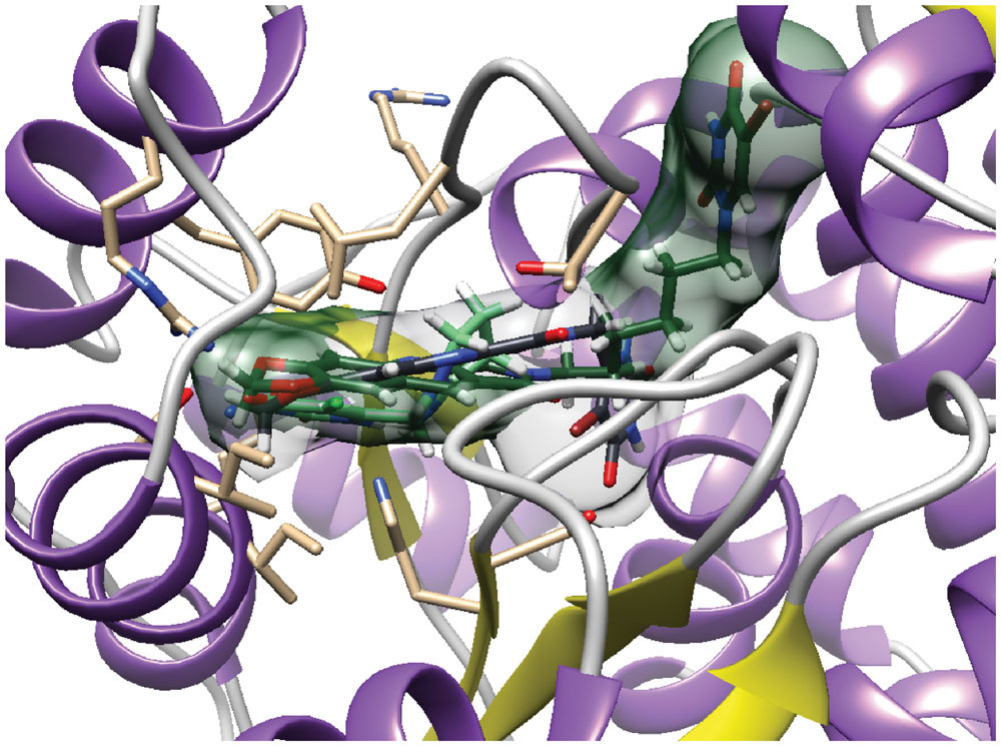
Docking superposition in the hTP active site of **TPI** (in green), compounds **16b** (dark green cloud), and **23b** (white cloud).

Surprisingly, docking study showed that in both series, the dioxolane of the pyrroloquinoline moiety is positioned in the same place as the pyrimidinedione of **TPI** suggesting interactions of the methylenedioxy group with the active site; the pyrimidinedione moiety interacting with a large pocket which has never been explored (superposition of compound **16b** and **TPI** on Figure 5).

However, as presented in Figure 5 for compound **16b** (dark green cloud) and compound **23b** (white cloud), two differences between series B and C were noticed. On the one hand, the tricyclic moieties were not superposed (180° vertical rotation). On the other hand, the aliphatic chains and pyrimidinediones of the B-series molecules were positioned in the upper part of the pockets, whereas pyrimidinediones of series C derivatives interacted with the lower part of this pocket. Interestingly, the phenyl nucleus of compound **2i** (series A) was also located in this white cloud (Supporting information, Figure S3).

#### 2.2.3. Series D

The B and C series modelling results led us to design the D-series in which the methylenedioxyphenyl rings were replaced by a pyrimidinedione with the aim to optimise interactions with the thymidine binding site of the natural ligand. Furthermore, various substituents have been introduced on the imide nitrogen in an attempt to explore the lower part of the “new” pocket.

In a physicochemical point of view, series D compounds are more soluble than the previous series. However, they were uninteresting in terms of enzymatic inhibition activity except for the N-benzyl derivative **28a** (Table 4).

**Figure 6.**
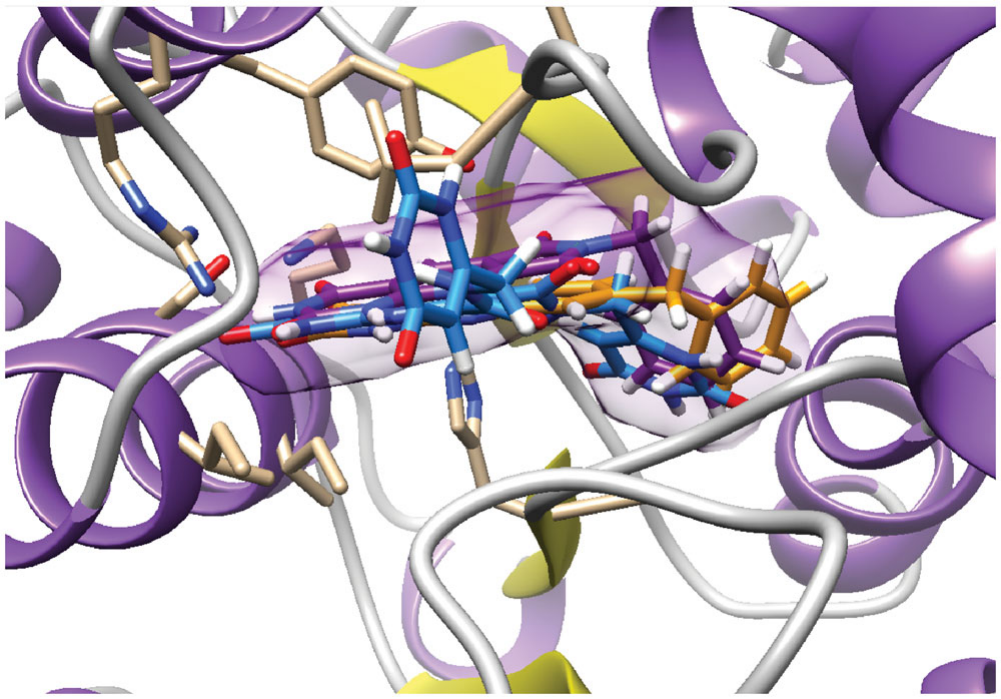
Docking superposition in the hTP active site of compounds **2i** (in orange), **28a** (in purple), and **30** (in blue).

As expected, the tricyclic moiety of compound **28a** was well superposed with compound **2i**, the benzyl analog in series A. The benzyl nuclei were positioned in the same area but not in the same position (Figure 6).

Surprisingly, the flexible open intermediate derivative **30** was as active as compound **28a**. Interestingly, one 6-pyrimidinomethylamino arm was positioned in the same area as previously described compounds (Figure 6), the other one was located at the entry of the pocket.

### 2.3. Mechanism of enzyme inhibition

The synthesised compounds have been designed as TP inhibitors interacting with the thymidine fixation site. However, TP being a two substrates enzyme, it was necessary to verify that new inhibitors do not bind to the phosphate site. This study has been realised with one of the most active and/or the most soluble compounds in each series, that is, compounds **2p**, **23b**, **28a**, and **30**. *E. coli* TP activity was determined in the presence of a saturating concentration of thymidine (equivalent to three times the value of the Michaelis-Menten constant [km]^52^) and variable amounts of phosphate (2–30 mM). Tested compounds have been used at a concentration corresponding to the IC_50_. For each tested derivative, phosphate concentration did not have any significant influence on the percentage of inhibition, indicating that the selected inhibitors did not bind to the phosphate site.

**Figure 7.**
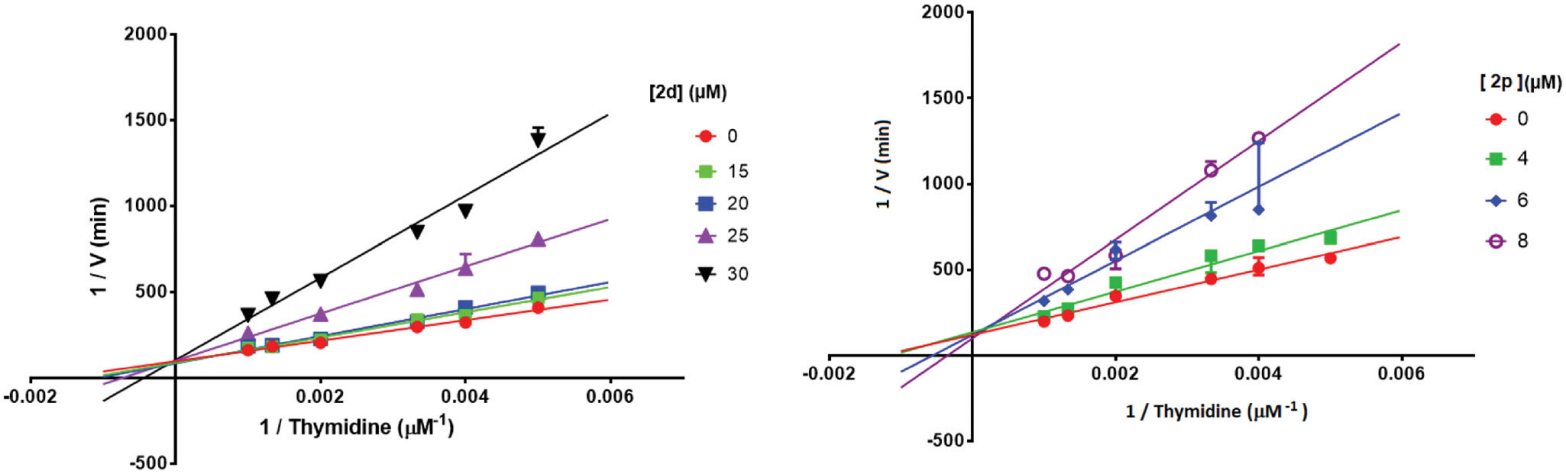
Lineweaver–Burk plots of *E. coli* TP inhibition by **2d** and **2p**, in the presence of variable concentrations of thymidine demonstrating competitive-type enzyme inhibition. Results are presented as means ± SD; SD denoted by error bars (experiments carried out in triplicate).

To explore whether **2d** and **2p**, the most active compounds which showed the same activities as 7-DX (TP CI_50_: 26–28 µM), acted as a competitive, uncompetitive, or mixed TP inhibitor, a brief kinetic study was performed according to previously described method^51^. The percentage of TP inhibition was determined for different concentrations of the inhibitors and thymidine. Lineweaver-Burk plots showed that all straight lines converged at the same point on the positive side of the y-axis (Figure 7). Consequently, **2d** and **2p** exhibited competitive inhibition kinetic on TP with thymidine as substrate. This was further supported by the fact that the Km values increased in the presence of **2d** and **2p**, while the Vmax values did not change significantly as the inhibitor concentration increased.

### 2.4. Cell proliferation assay (MTT)

The two most active compounds against TP (compounds **2d** and **2p**) were evaluated for growth-inhibiting properties in two cells lines namely human umbilical vein endothelial cells (HUVEC) and epidermal carcinoma cells (A431) which are well known to overexpress epidermal growth factor receptor (EGFR), a pathway involved in the cell proliferation and angiogenesis.

The inhibitory effect on cell proliferation was assessed using MTT assay^53^ after 72 h of treatment through dose-response assays performed in the 100 µM to 0.1 µM concentration range. The results were expressed as percentages of growth inhibition.

Compound **2d** exhibited better activity against A431 cell line (57% at 50 µM) than against HUVEC cell line (56% at 100 µM) but antiproliferative effect remains low. Compound **2p** can be considerate as less cytotoxic against the two cell lines (50% and 31% at 100 µM against respectively A431 and HUVEC cells).

## 3. Conclusion

In conclusion, a small library of 38 derivatives has been synthesised and evaluated for its TP inhibiting activity. Around the pharmacophoric pyrimidinedione core of the natural substrate thymidine, four series have been designed in order to interact with wide empty pockets of the active site.

The natural ligand has been annealed to a quinoline in series A (pyrimidoquinoline-2,4-diones), substituted *via* a methylenic chain by a quinolopyrrolidinedione in the more flexible series B and C, and the polycyclic heterocycle has been replaced by a pyrimidopyridopyrrolidinetetraone in series D. The tricyclic moieties of these new polycyclic nitrogen heterocycles have been synthesised by one-pot multicomponent reactions that involved an aniline, an aldehyde, and a 1,3-dicarbonyl derivative or an analogue.

The biological evaluation identified several structurally distinct TP inhibitors in series A and D. Among them, compounds **2d**, **2l**, **2p**, **28a**, and surprisingly the open intermediate **30** showed a modest to good TP inhibition (IC_50_ values ranging from 26 to 87 µM) when compared to **7-DX** used as a positive control. The two most active compounds **2d** and **2p** were shown to interact with the thymidine fixation site and to exhibit a competitive mode of inhibition towards TP.

Molecular docking analysis confirmed the interaction of these newly synthesised compounds at the active binding site of TP. Moreover, docking studies highlighted a plausible specific interaction in a wide pocket that had not been yet explored.

For the first time, our study showed that it is possible to inhibit TP with tricyclic heterocycles. It is worth noting that the active compounds have in common a pyrido[2,3-*d*]pyrimidinedione nucleus and possess a chain interacting with the same part of this pocket. Interestingly, the open intermediate **30** becomes a starting point for a novel series that will be further exploited in order to improve the activity against TP.

## 4. Experimental section

### 4.1. Chemistry

*General procedures:* Commercial reagents were used as received without further purification. Microwave irradiation reaction was performed with an InitiatorTM 2.0 device, Biotage. Reactions were followed with thin-layer chromatography (TLC) (using 0.20 mm silica or alumina gel 60 F_254_ aluminium plates, Merck) and visualisation was achieved with UV light (254 and 365 nm). Purifications were achieved through recrystallization or flash chromatography (using 40–63 µM silica, Merck). ^1^H and ^13^C NMR spectra were recorded on a Bruker AC 300 or 400 spectrometer by using DMSO-d_6_ as the solvent and internal standard. Chemical shifts are reported in ppm and coupling constants (*J*) are given in Hertz. Spin multiplicities are reported as follows: s = singlet, d = doublet, dd = doublet of doublet, m = multiplet, q = quadruplet, quint = quintuplet, t = triplet. Melting points were measured on a Stuart SMP3 melting point apparatus and are uncorrected. IR spectra were obtained on Perkin-Elmer 1600 spectrophotometer. Elemental analyses were performed at the CNRS Analysis Laboratory, Gif-sur-Yvette, France.

#### 4.1.1. Pyrimido[4,5-b]quinoline-2,4 (1H, 3H) -diones (series A)

##### 4.1.1.1. General procedure for the synthesis of compounds 2

A suspension of the requisite aniline (1.00 mmol, 1.0 eq.), barbituric acid **1** (128 mg, 1.00 mmol, 1.0 eq.), and paraformaldehyde (30.0 mg, 1.00 mmol, 1.0 eq.) was refluxed in AcOH (100 mL, method A; 300 mL, method B) or heated at 120 °C in a 1:1 mixture of AcOH/DMF (10 mL, method C). Reaction times and yields are given in Table 1.

##### 4.1.1.2. 8-Phenoxypyrimido[4,5-b]quinoline-2,4(1H,3H)-dione (2e)

Method C using 3-phenoxyaniline. After removal of the solvent under vacuum, the solid was boiled in H_2_O (10 mL) for 1 h, filtered and washed successively with H_2_O, EtOH and Et_2_O. Recrystallization from DMF afforded **2e** as a yellowish solid. mp: 334–336 °C (decomposition). ^1^H NMR (DMSO-d_6_, 400 MHz) *δ*: 11.61 (s, 1H), 11.46 (s, 1H), 8.95 (s, 1H), 8.16 (d, *J* = 9.0 Hz, 1H), 7.5 (t, *J* = 7.8 Hz, 2H), 7.34–7.28 (m, 2H), 7.22 (d, *J* = 7.8 Hz, 2H) 6.98 (d, *J* = 2.0 Hz, 1H) ppm. ^13^C NMR (100 MHz, DMSO-d_6_) *δ*: 162.2, 161.6, 154.6, 151.1, 150.7, 150.7, 138.7, 132.1, 130.4, 125.2, 120.9, 120.5, 118.2, 110.4, 109.5 ppm. IR *ν*: 3136, 3057, 3041, 2904, 2839, 1733, 1678, 1613, 1591, 1509, 1487, 1464, 1425, 1403, 1377, 1340, 1287, 1264, 1222, 1127, 970, 840, 817, 790, 775, 749, 707, 684 cm^−1^. Anal. Calcd. for C_17_H_11_N_3_O_3_ · 0.25 H_2_O (309.79): C, 65.91; H, 3.74; N, 13.56. Found: C, 65.93; H, 3.87; N, 13.77%.

##### 4.1.1.3. 8-Benzylpyrimido[4,5-b]quinoline-2,4(1H,3H)-dione (2i)

Method C using 3-benzylaniline. Work up was the same as used for **2e**. Recrystallization from DMF afforded **2i** as a yellowish solid. mp: 297–299 °C (decomposition). ^1^H NMR (300 MHz, DMSO-d_6_) *δ*: 11.65 (s, 1H), 11.47 (s, 1H), 8.92 (s, 1H), 8.04 (d, *J* = 8.4 Hz, 1H), 7.64 (s, 1H), 7.41 (dd, *J* = 8.4 Hz, 1.3, 1H), 7.35–7.28 (m, 4H), 7.26–7.18 (m, 1H), 4.16 (s, 2H) ppm. ^13^C NMR (75 MHz, DMSO-d_6_) *δ*: 162.3, 150.7, 150.2, 149.8, 147.2, 140.2, 138.7, 129.8, 129.0, 128.6, 126.8, 126.3, 125.7, 123.1, 110.5, 41.3 ppm. IR *ν*: 3135, 3045, 2909, 2833, 1731, 1703, 1679, 1611, 1580, 1513, 1490, 1451, 1397, 1344, 1315, 1285, 1268, 1027, 829, 789, 749, 705, 677 cm^−1^. Anal. Calcd. for C_18_H_13_N_3_O_2_ · 0.25 H_2_O (307.81): C, 70.23; H, 4.42; N, 13.65. Found: C, 70.00; H, 4.54; N, 13.72%.

##### 4.1.1.4. 7,8-Dichloropyrimido[4,5-b]quinoline-2,4(1H,3H)-dione (2l)

Method A using 3,4-dichloroaniline. The suspension was filtered and washed successively with H_2_O and Et_2_O to afford pure **2l** as a yellow solid. mp: > 360 °C. ^1^H NMR (400 MHz, DMSO-d_6_) *δ*: 11.97 (s, 1H), 11.88 (s, 1H), 11.61 (s, 1H), 9.04 (s, 1H), 8.54 (s, 1H), 8.09 (s, 1H) 1.91 (s, 3H) ppm. IR *ν*: 3179, 3053, 3024, 2837, 1690, 1655, 1624, 1578, 1560, 1481, 1450, 1418, 1369, 1335, 1265, 1182, 1126, 1034 cm^−1^. Anal. Calcd. for C_11_H_5_Cl_2_N_3_O_2_ · CH_3_COOH (342.13): C, 45.64; H, 2.65; N, 12.28. Found: C, 45.40; H, 2.39; N, 12.23%.

##### 4.1.1.5. Benzo[h]pyrimido[4,5-b]quinoline-8,10(9H,11H)-dione) (2n)

Method A using 1-naphthylamine. Work up was the same as used for **2l** to afford pure **2n** as a pink solid. mp: 326–328 °C. ^1^H NMR (400 MHz, DMSO-d_6_) *δ*: 11.88 (s, 1H), 11.57 (s, 1H), 9.08 (d, *J* = 8.0 Hz, 1H), 9.00 (s, 1H), 8.05 (d, *J* = 8.0 Hz, 1H), 8.00 (d, *J* = 8.0 Hz, 1H), 7.88 (d, *J* = 8.0 Hz, 1H), 7.84–7.74 (m, 2H) ppm. ^13^C NMR (100 MHz, DMSO-d_6_) *δ*: 162.4, 150.7, 150.1, 148.5, 137.9, 134.8, 129.8, 129.4, 128.2, 127.1, 126.0, 125.9, 124.7, 122.4, 110.4 ppm. IR *ν*: 3063, 3038, 3022, 3007, 2841, 1688, 1645, 1618, 1601, 1578, 1559, 1541, 1508, 1473, 1437, 1419, 1394, 1327, 1281, 1271, 1225, 1213, 1196, 1146 cm^−1^. Anal. Calcd. for C_15_H_9_N_3_O_2_ · 1.25 H_2_O (285.77): C, 63.04; H, 4.06; N, 14.70. Found: C, 62.93; H, 3.58; N, 14.97%.

##### 4.1.1.6. Naphto[2,3-h]pyrimido[4,5-b]quinoline-2,4(1H,3H)-dione (2o)

Method B using 1-aminoanthracene. Work up was the same as used for **2e**. Recrystallization from DMF afforded **2o** as a green-brownish solid. mp: > 360 °C. ^1^H NMR (400 MHz, DMSO-d_6_) *δ*: 11.96 (s, 1H), 11.58 (s, 1H), 9.66 (s, 1H), 8.93 (s, 1H), 8.62 (s, 1H), 8.25 (d, *J* = 8.0 Hz, 1H), 8.21 (d, *J* = 8.0 Hz, 1H), 7.91 (q, *J* = 8.0 Hz, 2H), 7.70 (m, 2H) ppm. ^13^C NMR (100 MHz, DMSO-d_6_) *δ*: 162.8, 151.1, 150.9, 150.5, 137.6, 133.7, 132.4, 131.7, 129.4, 128.4, 128.3, 127.9, 127.2, 127.0, 126.8, 125.9, 125.2, 122.9, 110.8 ppm. IR *ν*: 3042, 3028, 3011, 2992, 2974, 1713, 1682, 1609, 1585, 1566, 1541, 1506, 1489, 1454, 1393, 1337, 1277, 1206, 1038 cm^−1^. Anal. Calcd. for C_19_H_11_N_3_O_2_ · 0.75 H_2_O (326.82): C, 69.82; H, 3.86; N, 12.86. Found: C, 69.98; H, 3.38; N, 12.95%.

##### 4.1.1.7. 6-(3,4-Methylenedioxyphenylamino)pyrimidine-2,4(1H,3H)-dione (5)

A suspension of 6-chloropyrimidinedione (**3**) (146 mg, 1.00 mmol, 1.0 eq.) and methylenedioxyaniline (**4**) (548 mg, 4.00 mmol, 4.0 eq.) in DMAC. After evaporation of the solvent under reduced pressure, Et_2_O was added. The resulting suspension was filtered and washed with Et_2_O and MeOH to give crude **5** (185 mg, 75% yield) which was used without further purification in the next step. An analytical sample was obtained by recrystallization of a small amount from MeOH. mp: 305 °C. ^1^H NMR (400 MHz, DMSO-d_6_) *δ*: 10.40 (s, 1H), 10.16 (s, 1H), 8.01 (s, 1H), 6.91 (d, *J* = 9.0 Hz, 1H), 6.83 (d, *J* = 2.0 Hz, 1H), 6.67 (dd, *J* = 9.0 and 2.0 Hz, 1H), 6.04 (s, 2H), 4.46 (s, 1H) ppm. ^13^C NMR (100 MHz, DMSO-d_6_) *δ*: 164.7, 153.7, 151.3, 148.2, 145.4, 131.9, 117.9, 108.9, 106.3, 101.9, 75.6 ppm. IR *ν*: 3298, 2899, 1732, 1625, 1490, 1395, 1354, 1326, 1288, 1248, 1226, 1199, 1102, 1040, 990, 928, 813, 728, 757, 649, 600, 547, 531 cm^−1^. Anal. Calcd. for C_11_H_9_N_3_O_4_ · 0.25 H_2_O (251.71): C, 52.49; H, 3.80; N, 16.69. Found: C, 52.16; H, 3.67; N, 16.82%.

##### 4.1.1.8. 5-Dimethylamino-7,8-methylenedioxypyrimido[4,5-b]quinoline-2,4(1H,3H)-dione (2s)

To a suspension of **5** (247 mg, 1.00 mmol, 1.0 eq.) in anhydrous chlorobenzene (11 mL) was added Viehe’s salt (195 mg, 1.20 mmol, 1.2 eq.). The reaction mixture was refluxed for 12 h. The resulting solid was isolated by filtration, washed with H_2_O and recrystallized from DMF to give **2s** as a yellow solid (186 mg, 62% yield). mp: 348 °C. ^1^H NMR (400 MHz, DMSO-d_6_) *δ*: 11.15 (s, 1H), 10.94 (s, 1H), 7.44 (s, 1H), 7.05 (s, 1H), 6.20 (s, 2H), 3.06 (s, 6H) ppm. ^13^C NMR (100 MHz, DMSO-d_6_) *δ*: 161.4, 159.3, 152.6, 151.3, 150.8, 148.8, 146.4, 119.2, 104.3, 102.7, 102.0, 101.8, 44.5 ppm. IR *ν*: 3166, 3053, 2922, 1732, 1691, 1670, 1614, 1577, 1523, 1468, 1433, 1309, 1254, 1231, 1045, 801, 573, 535.cm^−1^. Anal. Calcd. for C_14_H_12_N_4_O_4_ · 0.5 H_2_O (309.28): C, 54.37; H, 4.24; N, 18.12. Found: C, 54.07; H, 3.88; N, 17.94%.

#### 4.1.2. 2H-Pyrroloquinoline-1,3-diones/pyrimidinediones (series B and C)

##### 4.1.2.1. Diethyl 6,7-methylenedioxyquinoline-2,3-dicarboxylate (8)

To a solution of aniline **4** (137 mg, 1.00 mmol, 1.0 eq.) in EtOH (10 mL) was added 37% aqueous formaldehyde solution (0.24 mL, 3.00 mmol, 3.0 eq.) and ethyl acetylene dicarboxylate (**7**) (0.16 mL, 1.00 mmol, 1.0 eq.). The reaction mixture was refluxed for 1 h. After evaporation of the solvent under vacuum, the residue was purified by flash column chromatography, using DMC as an eluant to afford **8** as a yellow powder (127 mg, 40% yield). mp: 132.5–133.5 °C. ^1^H NMR (400 MHz, DMSO-d_6_) *δ*: 9.23 (s, 1H), 7.43 (s, 1H), 7.07 (s, 1H), 6.17 (s, 2H), 4.56 (q, *J* = 7.0 Hz, 2H), 4.45 (q, *J* = 7.0 Hz, 2H), 1.45 (t, *J* = 7.0 Hz, 3H), 1.42 (t, *J* = 7.0 Hz, 3H) ppm. ^13^C NMR (100 MHz, DMSO-d_6_) *δ*: 167.2, 164.6, 152.7, 149.4, 149.0, 148.0, 140.7, 120.5, 117.9, 106.1, 102.5, 100.7, 62.3, 61.2, 14.2, 14.1 ppm. IR *ν*: 2983, 2968, 2928, 1757, 1723, 1573, 1502, 1467, 1368, 1344, 1315, 1278, 1198, 1150, 1104, 1049, 1035, 1020, 941, 851, 760, 742, 669, 637 cm^−1^. Anal. Calcd. for C_16_H_15_NO_6_ (317.29): C, 60.57; H, 4.77; N, 4.41. Found: C, 60.61; H, 4.72; N, 4.21%.

##### 4.1.2.2. 6,7-Methylenedioxypyrrolo[3,4-b]quinoline-1,3-dione (10)

*Method A:* To a solution of Na (23 mg, 1.00 mmol, 2.8 eq.) in EtOH (0.5 mL) were added **8** (115 mg, 0.36 mmol, 1.0 eq.) and urea (34 mg, 0.56 mmol, 1.6 eq.). The reaction mixture was heated under reflux for 1.5 h. The resulting brown solid was isolated by filtration, washed with a 1:1 AcOH/H_2_O mixture (5 mL) to afford **10** as a yellow solid (10 mg, 12% yield). *Method B:* 3,4-Methylenedioxyaniline (**4**) (411 mg, 3.00 mmol, 1.0 eq.), paraformaldehyde (270 mg, 9.00 mmol, 3.0 eq.) and 3-bromomaleimide **9** (528 mg, 3.00 mmol, 1.0 eq.) were stirred in EtOH (30 mL) at r.t. for 1 night. The reaction mixture was then heated at 60 °C for 6 h. The resulting suspension was filtered off and the solid was washed with EtOH and H_2_O to afford crude **10** (283 mg, 39%) which was used without further purification. A small amount was recrystallized from DMF to give an analytical sample (yellow solid). mp: 366–368 °C (decomposition). ^1^H NMR (400 MHz, DMSO-d_6_) *δ*: 11.50 (s, 1H), 9.01 (s, 1H), 7.88 (s, 1H), 7.57 (s, 1H), 6.35 (s, 2H) ppm. ^13^C NMR (100 MHz, DMSO-d_6_) *δ*: 170.2, 169.3, 153.6, 151.2, 151.0, 141.3, 134.6, 123.2, 119.0, 106.3, 103.8, 98.7 ppm. IR *ν*: 3449, 2956, 2723, 1771, 1720, 1623, 1573, 1471, 1411, 1330, 1265, 1100, 1030, 935, 872, 790, 748 cm^−1^. Anal. Calcd. for C_12_H_6_N_2_O_4_ · 0.25 H_2_O (246.69): C, 58.42; H, 2.66; N, 11.36. Found: C, 58.35; H, 2.49; N, 11.35%.

##### 4.1.2.3. General method for preparation of compounds 12 and 15

To a suspension of thymine (**11**) or 5-bromouracile (**14**) (4.00 mmol, 1.0 eq.) in MeCN (6 mL) was added BSA (2.5 mL, 10.00 mmol, 2.5 eq.) at r.t. When the reaction mixture became clear (15 min), the ω-dibromoalkyl derivative (6.00 mmol, 1.5 eq.) and I_2_ (cat. amount) were added. The mixture was refluxed for 2 h and then either maintained at r.t. for 1 night (**12a,b**) or refluxed for 2 days (**15**). After elimination of the solvent under vacuum, the residue was stirred with water (10 mL), isolated by filtration, and then purified by flash column chromatography.

##### 4.1.2.4. 1-(4-Bromobutyl)-5-methylpyrimidine-2,4(1H,3H)-dione (12a)

Chromatography solvents: DCM/MeOH, 100:0 to 97:3. White solid, 720 mg, 69% yield. mp: 143 °C. ^1^H NMR (300 MHz, DMSO-d_6_) *δ*: 11.23 (s, 1H), 7.54 (s, 1H), 3.65 (t, *J* = 7.0 Hz, 2H), 3.55 (t, *J* = 7.0 Hz, 2H), 1.75 (m, 7H) ppm. ^13^C NMR (75 MHz, DMSO-d_6_) *δ*: 164.5, 151.5, 141.8, 109.2, 46.8, 35.0, 29.6, 27.8, 12.4 ppm. IR *ν*: 3161, 3033, 2929, 2858, 2834, 1692, 1673, 1474, 1426, 1356, 1270, 1222, 872, 765, 692, 560 cm^−1^. Anal. Calcd. for C_9_H_13_BrN_2_O_2_ (261.11): C, 41.40; H, 5.02; N, 10.73. Found: C, 41.59; H, 4.86; N, 10.73%.

##### 4.1.2.5. 1-(6-Bromohexyl)-5-methylpyrimidine-2,4(1H,3H)-dione (12b)

Chromatography solvents: DCM/MeOH, 100:0 to 97:3. White solid, 600 mg, 52% yield. mp: 118 °C. ^1^H NMR (300 MHz, DMSO-*d_6_*) *δ*: 11.20 (s, 1H), 7.53 (s, 1H), 3.60 (t, *J* = 7.0 Hz, 2H), 3.52 (t, *J* = 7.0 Hz, 2H), 1.79 (quint, *J* = 7.0 Hz, 2H), 1.75 (s, 3H), 1.56 (quint, *J* = 7.0 Hz, 2H), 1.40 (quint, *J* = 7.0 Hz, 2H), 1.26 (quint, *J* = 7.0 Hz, 2H) ppm. ^13^C NMR (75 MHz, DMSO-*d_6_*) *δ*: 164.7, 151.3, 141.9, 108.8, 47.5, 35.5, 32.5, 28.7, 27.6, 25.4, 12.4 ppm. IR *ν*: 3166, 3036, 2929, 1702, 1647, 1473, 1425, 1356, 1270, 1221, 1185, 1113, 1066, 927, 911, 871, 784, 764, 692, 560 cm^1^. Anal. Calcd. for C_11_H_17_BrN_2_O_2_ (289.17): C, 45.69; H, 5.93; N, 9.69. Found: C, 45.67; H, 5.68; N, 9.77%.

##### 4.1.2.6. 5-Bromo-1-(6-bromohexyl)pyrimidine-2,4(1H,3H)-dione (15)

Chromatography solvents: DCM/AcOEt, 90:10. White solid, 610 mg, 43% yield. mp: 151 °C. ^1^H NMR (300 MHz, DMSO-d_6_) *δ*: 11.73 (s, 1H), 8.24 (s, 1H), 3.65 (t, *J* = 7.0, 2H), 3.52 (t, *J* = 7.0, 2H), 1.79 (quint, *J* = 7.0, 2H), 1.58 (quint, *J* = 7.0, 2H), 1.39 (quint, *J* = 7.0, 2H), 1.26 (quint, *J* = 7.0, 2H) ppm. ^13^C NMR (100 MHz, DMSO-d_6_): *δ*: 160.1, 150.7, 145.8, 94.9, 48.2, 35.5, 32.5, 28.6, 25.6, 25.3 ppm. IR (KBr) *ν*: 3151, 3028, 2932, 2854, 1693, 1620, 1460, 1430, 1357, 1336, 1261, 1047, 749, 635, 560 cm^−1^. Anal. Calcd. for C_10_H_14_Br_2_N_2_O_2_ (354.04): C, 33.92; H, 3.99; N, 7.91. Found C, 34.31; H, 4.03; N, 7.85%.

##### 4.1.2.7. 2-[4-(2,4-Dioxo-5-methyl-3,4-dihydropyrimidine-1(2H)-yl)butyl]-6,7-methylenedioxy-2H-pyrrolo[3,4-b]quinoline-1,3-dione (13a)

A suspension of imide **10** (242 mg, 1.00 mmol, 1.0 eq.) and K_2_CO_3_ (166 mg, 1.20 mmol, 1.2 eq.) in anhydrous DMF (9 mL) was stirred for 0.5 h and then a solution of **12a** (390 mg, 1.50 mmol, 1.5 eq.) in anhydrous DMF (1 mL). was added. The reaction mixture was heated at 100 °C for 1 h. After evaporation of the solvent under vacuum, H_2_O (10 mL) was added to give a suspension which was filtered, washed with H_2_O, and purified by column chromatography (DCM/MeOH, 98:2 to 90:10) to afford **13a** as a yellow solid (130 mg, 31% yield). mp: 302 °C. ^1^H NMR (400 MHz, DMSO-d_6_) *δ*: 11.19 (s, 1H), 9.05 (s, 1H), 7.89 (s, 1H), 7.57 (s, 1H), 7.52 (s, 1H), 6.36 (s, 2H), 5.76 (CH_2_Cl_2_), 3.62 (m, 4H), 1.72 (s, 3H), 1.62 (m, 4H) ppm. ^13^C NMR (100 MHz, DMSO-d_6_) *δ*: 168.8, 168.1, 164.7, 153.8, 151.4, 151.3, 151.2, 141.9, 141.1, 133.9, 122.3, 118.9, 108.9, 106.3, 103.8, 98.7, 55.4 (CH_2_Cl_2_), 47.0, 37.6, 26.3, 25.3, 12.4 ppm. IR *ν*: 3062, 3039, 2991, 2817, 1770, 1761, 1690, 1625, 1496, 1467, 1434, 1397, 1364, 1268, 1233, 1180, 1104, 1032, 936, 913, 857, 803, 761, 747, 708, 569 cm^−1^. Anal. Calcd. for C_21_H_18_N_4_O_6_ · 0.5 CH_2_Cl_2_ (464.86): C, 54.72; H, 4.06; N, 11.87. Found: C, 54.84; H, 4.11; N, 11.76%.

##### 4.1.2.8. 2-[4-(2,4-Dioxo-5-methyl-3,4-dihydropyrimidine-1(2H)-yl)hexyl]-6,7-methylenedioxy-2H-pyrrolo[3,4-b]quinoline-1,3-dione (13b)

Starting from **12b** (432 mg, 1.50 mmol, 1.5 eq.), **13b** was prepared in the same procedure and work-up as described for **13a**. Yellow solid, 170 mg, 38% yield. mp: 262 °C. ^1^H NMR (400 MHz, DMSO-d_6_) *δ*: 11.18 (s, 1H), 9.02 (s, 1H), 7.86 (s, 1H), 7.54 (s, 1H), 7.51 (s, 1H), 6.37 (s, 2H), 3.58 (m, 4H), 1.73 (s, 3H), 1.57 (m, 4H), 1.30 (m, 4H) ppm. ^13^C NMR (100 MHz, DMSO-d_6_) *δ*: 168.7, 168.1, 164.7, 153.8, 151.3, 151.2, 141.8, 141.1, 133.8, 122.2, 118.9, 108.8, 106.3, 103.8, 98.6, 47.5, 37.9, 28.8, 28.2, 26.3, 25.9, 12.4 ppm. IR *ν*: 3042, 2936, 2854, 2817, 1768, 1712, 1666, 1623, 1498, 1463, 1429, 1395, 1351, 1310, 1267, 1232, 1181, 1033, 941, 914, 885, 864, 810, 793, 760, 566 cm^−1^. Anal. Calcd. for C_23_H_22_N_4_O_6_ · 0.25 H_2_O (454.95): C, 60.72; H, 4.99; N, 12.31. Found: C, 60.73; H, 4.82; N, 12.39%.

##### 4.1.2.9. 2-[4-[5-Bromo-3-[4-(5-bromo-2,4-dioxo-3,4-dihydropyrimidine-1(2H)-yl)hexyl]-2,4-dioxo-3,4-dihydropyrimidine-1(2H)-yl]hexyl]-6,7-méthylènedioxy-2H-pyrrolo[3,4-b]quinoline-1,3-dione (17)

To a suspension of imide **10** (200 mg, 0.82 mmol, 1.0 eq.) and K_2_CO_3_ (136 mg, 0.924 mmol, 1.2 eq.) in anhydrous DMF (8 mL) was added, drop by drop, a solution of **15** (400 mg, 1.23 mmol, 1.5 eq.) in anhydrous DMF (12 mL). The mixture was heated at 100 °C for 6.5 h. After removing the solvent under vacuum, the residue was stirred with water (10 mL) and a aqueous 1 N HCl solution was added until a pH = 3 was reached. The resulting solid was isolated by filtration, washed with H_2_O and EtOH and recrystallized from MeOH to afford **17** as a yellow solid (194 mg, 30% yield). mp: 144–148 °C. ^1^H NMR (400 MHz, DMSO-d_6_) *δ*: 11.72 (s, 1H), 9.05 (s, 1H), 8.28 (s, 1H), 8.21 (s, 1H), 7.89 (s, 1H), 7.57 (s, 1H), 6.36 (s, 2H), 3.69 (m, 8H), 1.54 (m, 8H), 1.30 (m, 8H) ppm. ^13^C NMR (100 MHz, DMSO-d_6_) *δ*: 168.8, 168.1, 160.1, 159.2, 153.8, 151.3, 151.2, 150.8, 150.7, 150.6, 145.7, 144.3, 141.1, 133.8, 122.2, 118.9, 106.3, 103.8, 98.7, 94.9, 94.4, 49.4, 48.2, 42.1, 37.9, 28.7, 28.6, 28.2, 27.2, 26.2, 25.8, 25.7 ppm. IR *ν*: 3186, 3057, 2934, 2858, 1768, 1709, 1655, 1460, 1343, 1262, 1228, 1178, 1032, 939, 865, 803, 760, 620, 574 cm^−1^. Anal. Calcd. for C_32_H_32_Br_2_N_6_O_8_ · 0.5 H_2_O (797.45): C, 48.20; H, 4.17; N, 10.54. Found: C, 48.03; H, 3.94; N, 10.61%.

##### 4.1.2.10. 3-Benzoyl-5-bromo-1-(4-bromobutyl)pyrimidine-2,4(1H,3H)-dione (19a)

1,4-Dibromobutane (3.24 mL, 24.00 mmol) was added to a suspension of **18**^48^ (885 mg, 3.00 mmol) and K_2_CO_3_ (1.656 g, 12.00 mmol, 4.0 eq.) in anhydrous DMF (40 mL). The mixture was stirred at r.t. for 2 h. After removing the solvent under vacuum, the residue was stirred with water (10 mL), isolated by filtration, and purified by flash column chromatography using DCM as a solvent to give **19a** as a white solid (1.058 g, 82% yield). mp: 129 °C. ^1^H NMR (300 MHz, DMSO-d_6_) *δ*: 8.51 (s, 1H), 8.04 (d, *J* = 8.0 Hz, 2H), 7.81 (t, *J* = 8.0 Hz, 1H), 7.61 (t, *J* = 8.0 Hz, 2H), 3.79 (t, *J* = 6.0 Hz, 2H), 3.58 (t, *J* = 6.0 Hz, 2H), 1.81 (m, 4H) ppm. ^13^C NMR (75 MHz, DMSO-d_6_) *δ*: 167.9, 158.5, 149.2, 143.9, 135.6, 130.9, 130.5, 129.4, 96.1, 48.5, 32.7, 29.2, 27.7 ppm. IR *ν*: 3081, 2961, 1751, 1702, 1655, 1619, 1598, 1427, 1243, 1179, 1091, 985, 937, 924, 798, 782, 761, 743, 708, 686, 657, 563, 546 cm^−1^. Anal. Calcd. for C_15_H_14_Br_2_N_2_O_3_ (430.09): C, 41.89; H, 3.28; N, 6.51. Found: C, 41.73; H, 3.21; N, 6.47%.

##### 4.1.2.11. 3-Benzoyl-5-bromo-1-(4-bromohexyl)pyrimidine-2,4(1H,3H)-dione (19b)

Starting from 1,6-dibromohexane (7.23 mL, 24.00 mmol), **19b** was prepared in the same procedure, work-up and purification as described for **19a**. White solid (1.18 g, 86% yield). mp: 89 °C. ^1^H NMR (300 MHz, DMSO-d_6_) *δ*: 8.51 (s, 1H), 8.01 (d, *J* = 8.0 Hz, 2H), 7.80 (t, *J* = 8.0 Hz, 1H), 7.61 (t, *J* = 8.0 Hz, 2H), 3.74 (t, *J* = 7.0 Hz, 2H), 3.52 (t, *J* = 7.0 Hz, 2H), 1.80 (quint, *J* = 7.0 Hz, 2H), 1.65 (quint, *J* = 7.0 Hz, 2H), 1.41 (quint, *J* = 7.0 Hz, 2H), 1.30 (quint, *J* = 7.0 Hz, 2H) ppm. ^13^C NMR (75 MHz, DMSO-d_6_) *δ*: 169.2, 158.9, 149.5, 146.8, 136.2, 131.2, 131.0, 130.0, 94.5, 48.9, 35.6, 32.5, 28.5, 27.5, 25.2 ppm. IR *ν*: 2939, 1745, 1699, 1662, 1619, 1599, 1427, 1352, 1333, 1255, 1189, 986, 708, 686, 659, 559 cm^−1^. Anal. Calcd. for C_17_H_18_Br_2_N_2_O_3_ (458.14): C, 44.57; H, 3.96; N, 6.11. Found: C, 44.46; H, 3.86; N, 6.04%.

##### 4.1.2.12. 2-[4-(3-Benzoyl-5-bromo-2,4-dioxo-3,4-dihydropyrimidine-1(2H)-yl)butyl]-6,7-methylenedioxy-2H-pyrrolo[3,4-b]quinoline-1,3-dione (20a)

To a suspension of **10** (242 mg, 1.00 mmol, 1.0 eq.) and K_2_CO_3_ (166 mg, 1.20 mmol, 1.2 eq.) in anhydrous DMF (10 mL) was added **19a** (645 mg, 1.50 mmol, 1.5 eq.). The mixture was stirred at r.t. for 0.5 h. After elimination of the solvent by evaporation under vacuum, the residue was purified by flash column chromatography (DCM/EtOAc, 95:5 to 80:20) to afford **20a** (496 mg, 84% yield). mp: 257 °C. ^1^H NMR (400 MHz, DMSO-d_6_) *δ*: 9.07 (s, 1H), 8.48 (s, 1H), 8.01 (d, *J* = 9.0 Hz, 2H), 7.91 (s, 1H), 7.79 (t, *J* = 9 Hz, 1H), 7.60 (m, 3H), 6.37 (s, 2H), 3.78 (t, *J* = 6.0 Hz, 2H), 3.63 (t, *J* = 6.0 Hz, 2H), 1.68 (m, 4H) ppm. ^13^C NMR (100 MHz, DMSO-d_6_) *δ*: 169.1, 168.8, 168.1, 158.9,153.8, 151.3, 151.2, 149.5, 146.8, 141.1, 136.2, 133.8, 131.1, 131.0, 130.0, 122.2, 118.9, 106.3, 103.8, 98.6, 94.5, 48.6, 37.6, 26.1, 25.3 ppm. IR *ν*: 2920, 1742, 1704, 1659, 1618, 1455, 1426, 1399, 1330, 1277, 1259, 1230, 1177, 1085, 1032, 940, 912, 867, 802, 758, 712, 658, 572 cm^−1^. Anal. Calcd. for C_27_H_19_BrN_4_O_7_ (591.37): C, 54.84; H, 3.24; N, 9.47. Found: C, 55.01; H, 3.52; N, 9.08%.

##### 4.1.2.13. 2-[6-(3-Benzoyl-5-bromo-2,4-dioxo-3,4-dihydropyrimidine-1(2H)-yl)hexyl]-6,7-methylenedioxy-2H-pyrrolo[3,4-b]quinoline-1,3-dione (20b)

Starting from **19b** (596 mg, 1.30 mmol, 1.3 eq.), **20b** was prepared in the same procedure, work-up and purification as described for **20a.** Yellow solid (520 mg, 84% yield). mp: 196 °C. ^1^H NMR (400 MHz, DMSO-d_6_) *δ*: 9.05 (s, 1H), 8.48 (s, 1H), 8.01 (d, *J* = 6.0 Hz, 2H), 7.90 (s, 1H), 7.78 (t, *J* = 6 Hz, 1H), 7.59 (m, 3H), 6.36 (s, 2H), 3.74 (t, *J* = 6.0 Hz, 2H), 3.59 (t, *J* = 6.0 Hz, 2H), 1.62 (m, 4H), 1.32 (m, 4H) ppm. ^13^C NMR (100 MHz, DMSO-d_6_) *δ*: 169.1, 168.8, 168.1, 158.7,153.8, 151.3, 151.2, 149.4, 146.7, 141.1, 136.1, 133.8, 131.2, 131.0, 130.0, 122.2, 118.9, 106.3, 103.8, 98.7, 94.4, 48.9, 37.8, 28.6, 28.2, 26.2, 25.7 ppm. IR *ν*: 2933, 1746, 1704, 1663, 1621, 1598, 1498, 1458, 1425, 1396, 1334, 1318, 1260, 1230, 1176, 1033, 973, 943, 865, 799, 769, 757, 657, 572 cm^−1^. Anal. Calcd. for C_29_H_23_BrN_4_O_7_. 0.25 H_2_O (623.92): C, 55.83; H, 3.80; N, 8.98. Found: C, 55.71; H, 3.84; N, 8.79%.

##### 4.1.2.14. 2-[4-(5-Bromo-2,4-dioxo-3,4-dihydropyrimidine-1(2H)-yl)butyl]-6,7-methylenedioxy-2H-pyrrolo[3,4-b]quinoline-1,3-dione (16a)

To a suspension of **19a** (387 mg, 0.65 mmol, 1.0 eq.) in EtOH (6 mL) was added an aqueous 5 N HCl solution (4 mL). The mixture was refluxed for 2 days. After cooling at r.t., the solid was collected by filtration, washed with H_2_O. Recrystallization from DMF afforded **16a** as a yellow solid (168 mg, 53% yield). mp: 276 °C. ^1^H NMR (400 MHz, DMSO-d_6_) *δ*: 11.72 (s, 1H), 9.06 (s, 1H), 8.21 (s, 1H), 7.89 (s, 1H), 7.58 (s, 1H), 6.37 (s, 2H), 3.69 (t, *J* = 6.0 Hz, 2H), 3.63 (t, *J* = 6.0 Hz, 2H), 1.63 (m, 4H) ppm. ^13^C NMR (100 MHz, DMSO-d_6_) *δ*: 168.8, 168.1, 160.1, 153.8, 151.3, 151.2, 150.8, 145.8, 141.1, 133.9, 122.3, 118.9, 106.3, 103.8, 98.7, 95.0, 47.8, 37.6, 26.2, 25.2 ppm. IR *ν*: 3449, 3042, 2953, 2792, 1769, 1709, 1623, 1497, 1465, 1397, 1349, 1266, 1232, 1181, 1108, 1034, 938, 913, 868, 803, 749, 619, 576, 566 cm^−1^. Anal. Calcd. for C_20_H_15_BrN_4_O_6_ · 0.5 H_2_O (496.27): C, 48.40; H, 3.25; N, 11.29. Found: C, 48.17; H, 3.41; N, 11.64%.

##### 4.1.2.15. 2-[6-(5-Bromo-2,4-dioxo-3,4-dihydropyrimidine-1(2H)-yl)hexyl]-6,7-methylenedioxy-2H-pyrrolo[3,4-b]quinoline-1,3-dione (16b)

Starting from **20b** (520 mg, 0.84 mmol, 1.0 eq.), **16b** was prepared in the same procedure, work-up and purification as described for **16a.** Yellow solid (168 mg, 39% yield). mp: 282–283 °C. ^1^H NMR (400 MHz, DMSO-d_6_) *δ*: 11.72 (s, 1H), 9.04 (s, 1H), 8.22 (s, 1H), 7.89 (s, 1H), 7.57 (s, 1H), 6.36 (s, 2H), 3.64 (t, *J* = 6.0 Hz, 2H), 3.57 (t, *J* = 6.0 Hz, 2H), 1.59 (m, 4H), 1.30 (m, 4H) ppm. ^13^C NMR (100 MHz, DMSO-d_6_) *δ*: 168.8, 168.1, 160.1, 153.8, 151.3, 151.2, 150.7, 145.7, 141.1, 133.8, 122.2, 118.9, 106.3, 103.8, 98.7, 94.6, 48.2, 37.9, 28.7, 28.2, 26.3, 25.8 ppm. IR *ν*: 3043, 2989, 2934, 2788, 1770, 1714, 1691, 1622, 1495, 1468, 1438, 1392, 1349, 1335, 1269, 1231, 1146, 1181, 1037, 941, 868, 743, 617 cm^−1^. Anal. Calcd. for C_22_H_19_BrN_4_O_6_ (515.31): C, 51.28; H, 3.72; N, 10.87. Found: C, 51.04; H, 3.66; N, 10.87%.

##### 4.1.2.16. 2-[(2,6-Dioxo-1,2,3,6-tetrahydropyrimidin-4-yl)methyl]-6,7-methylenedioxy-1H-pyrrolo[3,4-b]quinoline-1,3(2H)-dione (22)

To a suspension **21** (482 mg, 3.00 mmol, 1.5 eq.) in anhydrous MeCN (10 mL) was added BSA (1.5 mL, 6.00 mmol, 3.0 eq.) at r.t. When the reaction mixture became clear (15 min), a suspension of imide **10** (484 mg, 2.00 mmol, 1.0 eq.) and K_2_CO_3_ (415 mg, 3.00 mmol, 1.5 eq.) in anhydrous MeCN (10 mL) was added and the mixture was refluxed for 5 days. Then, another amount of **21** (241 mg, 1.50 mmol, 0.75 eq.) and BSA (0.85 mL, 3.48 mmol, 1.74 eq.) in MeCN (5 mL) were added and the reflux was maintained for 2 more days. After evaporation of the solvent under vacuum, the residue was stirred with H_2_O (10 mL) and an aqueous 1 N HCl solution was added until a pH = 3 was reached. The resulting solid was isolated by filtration, washed with H_2_O and EtOH. Recrystallization from DMF afforded **22** as a yellow solid (579 mg, 79% yield). mp: 407–409 °C (decomposition). ^1^H NMR (400 MHz, DMSO-d_6_) *δ*: 11.07 (s, 1H), 11.03 (s, 1H), 9.10 (s, 1H), 7.89 (s, 1H), 7.60 (s, 1H), 6.37 (s, 2H), 5.55 (s, 1H), 4.51 (s, 2H) ppm. ^13^C NMR (100 MHz, DMSO-d_6_) *δ*: 167.8, 167.0, 163.8, 153.4, 151.3, 150.9, 150.8, 150.7, 140.8, 133.8, 122.1, 118.5, 105.9, 103.4, 98.2, 97.1, 37.4 ppm. IR *ν*: 3185, 3106, 3056, 3001, 2817, 1772, 1702, 1650, 1494, 1455, 1414, 1392, 1341, 1322, 1307, 1264, 1230, 1177, 1104, 1030, 1014, 930, 866, 847, 828, 794, 752 cm^−1^. Anal. Calcd. for C_17_H_10_N_4_O_6_ · 0.5 H_2_O (375.29): C, 54.41; H, 2.95; N, 14.93. Found: C, 54.10; H, 3.05; N, 14.94%.

##### 4.1.2.17. 2-[(5-Chloro-2,6-dioxo-1,2,3,6-tetrahydropyrimidin-4-yl)methyl]-6,7-methylenedioxy-1H-pyrrolo[3,4-b]quinoline-1,3(2H)-dione (23a)

To a suspension of **22** (183 mg, 0.50 mmol) in anhydrous DMF (5 mL), NCS (93.5 mg, 0.70 mmol, 1.4 eq.) was added and the mixture was stirred overnight at r.t. Then, the reaction was cooled at 0 °C and H_2_O (5 mL) was added. The resulting precipitate was isolated by filtration, washed successively with H_2_O and EtOH. Recrystallization from DMF afforded **23b** as a yellow solid (88 mg, 44% yield). mp: 352–354 °C (decomposition). ^1^H NMR (400 MHz, DMSO-d_6_) *δ*: 11.65 (s, 1H), 11.33 (s, 1H), 9.12 (s, 1H), 7.91 (s, 1H), 7.61 (s, 1H), 6.38 (s, 2H), 4.77 (s, 2H) ppm. ^13^C NMR (100 MHz, DMSO-d_6_) *δ*: 167.7, 166.9, 159.4, 153.5, 151.1, 150.7, 149.9, 146.7, 140.8, 133.8, 122.1, 118.4, 105.9, 104.3, 103.4, 98.1, 36.9 ppm. IR *ν*: 3256, 3180, 3143, 3051, 3005, 2808, 1773, 1702, 1668, 1618, 1501, 1462, 1420, 1386, 1344, 1316, 1259, 1224, 1105, 1035, 925, 892, 876, 796, 762, 750 cm^−1^. Anal. Calcd. for C_17_H_9_ClN_4_O_6_ · 0.5 H_2_O (409.74): C, 49.83; H, 2.46; N, 13.67. Found: C, 49.87; H, 2.22; N, 13.48%.

##### 4.1.2.18. 2-[(5-Bromo-2,6-dioxo-1,2,3,6-tetrahydropyrimidin-4-yl)methyl]-6,7-methylenedioxy-1H-pyrrolo[3,4-b]quinoline-1,3(2H)-dione (23b)

To a suspension of **22** (293 mg, 0.80 mmol) in anhydrous DMF (8 mL), NBS (214 mg, 1.20 mmol, 1.5 eq.) was added and the mixture was stirred overnight at r.t. Work up and purification were the same as used for **23a** to give **23b** as a yellow solid (210 mg, 59% yield). mp: 370–372 °C (decomposition). ^1^H NMR (400 MHz, DMSO-d_6_) *δ*: 11.64 (s, 1H), 11.37 (s, 1H), 9.14 (s, 1H), 7.92 (s, 1H), 7.63 (s, 1H), 6.39 (s, 2H), 4.74 (s, 2H) ppm. ^13^C NMR (100 MHz, DMSO-d_6_) *δ*: 168.3, 167.0, 160.2, 154.0, 151.6, 151.2, 150.7, 148.8, 141.3, 134.4, 122.7, 118.9, 106.4, 103.9, 98.6, 94.8, 39.7 ppm. IR *ν*: 3002, 2957, 2912, 2820, 1786, 1707, 1650, 1619, 1492, 1457, 1427, 1385, 1340, 1314, 1263, 1233, 1032, 930, 873, 861, 802, 757, 743, 733, 684, 662 cm^−1^. Anal. Calcd. for C_17_H_9_BrN_4_O_6_ · 0.75 H_2_O (458.69): C, 44.51; H, 2.31; N, 12.21. Found: C, 44.64; H, 2.22; N, 12.00%.

##### 4.1.2.19. 2-[(5-Iodo-2,6-dioxo-1,2,3,6-tetrahydropyrimidin-4-yl)methyl]-6,7-methylenedioxy-1H-pyrrolo[3,4-b]quinoline-1,3(2H)-dione (23c)

To a suspension of **22** (147 mg, 0.40 mmol) in anhydrous DMF (4 mL), NIS (135 mg, 0.60 mmol, 1.5 eq.) was added and the mixture was stirred overnight at r.t. Work up was the same as used for **23a** to give pure crude **23c** as a white solid (180 mg, 91% yield). mp: 365–367 °C (decomposition). ^1^H NMR (400 MHz, DMSO-d_6_) *δ*: 11.49 (s, 1H), 11.27 (s, 1H), 9.12 (s, 1H), 7.92 (s, 1H), 7.62 (s, 1H), 6.38 (s, 2H), 4.70 (s, 2H) ppm. ^13^C NMR (100 MHz, DMSO-d_6_) *δ*: 167.9, 167.1, 161.2, 153.5, 151.1, 150.8, 150.7, 150.6, 140.8, 134.0, 122.3, 118.4, 105.9, 103.4, 98.1, 70.4, 43.5 ppm. IR *ν*: 3040, 2948, 2912, 2814, 1785, 1725, 1703, 1649, 1614, 1492, 1458, 1437, 1387, 1338, 1314, 1264, 1233, 1032, 931, 873, 862, 803, 760, 743, 733 cm^−1^. Anal. Calcd. for C_17_H_9_IN_4_O_6_ (492.18): C, 41.49; H, 1.84; N, 11.38. Found: C, 41.10; H, 1.51; N, 11.08%.

##### 4.1.2.20. 6,7-Methylenedioxy-2-benzylpyrrolo[3,4-b]quinoline-1,3-dione (24)

A suspension of imide **10** (121 mg, 0.50 mmol) and K_2_CO_3_ (207 mg, 1.50 mmol, 3.0 eq.) in anhydrous DMF (7 mL) was stirred at r.t. After solubilisation of **10**, benzyl bromide (0.293 mL, 2.50 mmol, 5.0 eq.) was added. After 5 min, K_2_CO_3_ was removed by filtration, and H_2_O (40 mL) was added. The resulting precipitate was isolated by filtration and solubilised in DCM. The organic layer was dried over anhydrous Na_2_SO_4_, filtrated, and concentrated under reduced pressure. The residual solid was washed 4 times with Et_2_O to afford pure **24** as a yellowish solid (108 mg, 65% yield). mp: 191–193 °C. ^1^H NMR (300 MHz, DMSO-d_6_) *δ*: 9.05 (s, 1H), 7.85 (s, 1H,), 7.54 (s, 1H), 7.42–7.23 (m, 5H), 6.35 (s, 2H), 4.79 (s, 2H) ppm. ^13^C NMR (75 MHz, DMSO-d_6_) *δ*: 168.1, 167.4, 153.4, 150.9, 150.8, 140.8, 136.5, 133.3, 128.6, 127.5, 121.7, 118.5, 105.9, 103.4, 98.2, 41.0 ppm. IR *ν*: 3107, 3048, 2914, 1765, 1704, 1617, 1495, 1454, 1427, 1390, 1343, 1316, 1258, 1228, 1175, 1033, 938, 886, 860, 796, 757, 747, 703, 696 cm^−1^. Anal. Calcd. for C_19_H_12_N_2_O_4_ · 0.25 H_2_O (336.81): C, 67.75; H, 3.74; N, 8.32. Found: C, 67.46; H, 3.71; N, 8.06%.

#### 4.1.3. 1 H-Pyrrolo[3′,4′:5,6]pyrido[2,3-d]pyrimidine-2,4,6,8(3H,7H)-tetraones (series D)

##### 4.1.3.1. General method for preparation of compounds 28

To a suspension of **27**^28^ (70.0 mg, 0.30 mmol, 1.0 eq.) in DMF (444 µL), the requisite amine (0.60 mmol, 2.0 eq.) was added. The reaction mixture was refluxed for a time T_1_. To the resulting solution, PTSA (115 mg, 0.60 mmol, 2.0 eq.) was added and reflux was maintained during a time T_2_. The resulting precipitate was filtered off and washed to give a crude pure compound.

##### 4.1.3.2. 1H-7-(3,4,5-Trimethoxybenzyl)-pyrrolo[3′,4′:5,6]pyrido[2,3-d]pyrimidine-2,4,6,8 (3H,7H)-tetraone (28c)

T_1_ = 1 h, T_2_ = 17 h. Another amount of PTSA (0.5 eq.) was added and the reaction was heated at 120 °C for additional 24 h. The resulting precipitate was washed with DMF and H_2_O to afford pure **28c** as a yellow solid (81 mg, 66% yield). mp: 316–318 °C (decomposition). ^1^H NMR (400 MHz, DMSO-d_6_) *δ*: 12.50 (s, 1H), 11.88 (s, 1H), 8.51 (s, 1H), 6.64 (s, 2H), 4.73 (s, 2H), 3.73 (s, 6H), 3.61 (s, 3H) ppm. ^13^C NMR (75 MHz, DMSO-d_6_) *δ*: 165.3, 165.2, 161.5, 157.0, 156.1, 152.9, 150.0, 136.8, 131.9, 131.5, 121.3, 112.7, 104.9, 60.0, 55.9, 41.5 ppm. IR *ν*: 3439, 3182, 3075, 2838, 1743, 1707, 1676, 1614, 1589, 1550, 1511, 1458, 1424, 1394, 1382, 1346, 1331, 1281, 1242, 1191, 1127, 1105, 1038, 995, 966, 942, 835, 812, 795, 777, 752, 733, 697, 667 cm^−1^. Anal. Calcd. for C_19_H_16_N_4_O_7_ · H_2_O (430.37): C, 53.02; H, 4.22; N, 13.02. Found: C, 53.09; H, 4.25; N, 13.23%.

##### 4.1.3.3. 7-(Pyridin-3-ylmethyl)-1H-pyrrolo[3′,4′:5,6]pyrido[2,3-d]pyrimidine-2,4,6,8(3H,7H)-tetraone (28f)

T_1_ = 24 h, T_2_ = 24 h, washing solvent: H_2_O. **28f**: white solid (72 mg, 74% yield). mp: >360 °C. ^1^H NMR (400 MHz, DMSO-d_6_) *δ*: 12.50 (s, 1H), 11.87 (s, 1H), 8.60 (s, 1H), 8.55–8.44 (m, 2H), 7.77 (d, *J* = 7.9 Hz, 1H), 7.36 (dd, *J* = 7.9, 4.8 Hz, 1H), 4.85 (s, 2H) ppm. ^13^C NMR (75 MHz, DMSO-d_6_) *δ*: 165.2, 165.1, 161.5, 157.0, 156.1, 150.0, 149.0, 148.7, 135.6, 131.9, 131.5, 123.6, 121.3, 112.7, 38.9 ppm. IR *ν*: 3232, 3101, 3030, 2905, 2795, 1777, 1715, 1606, 1584, 1478, 1462, 1424, 1381, 1356, 1343, 1306, 1287, 1248, 1187, 1112, 1098, 1066, 1043, 985, 964, 932, 853, 837, 818, 795, 748, 723, 678 cm^−1^. Anal. Calcd. for C_15_H_9_N_5_O_4_ · 0.5 H_2_O (332.27): C, 54.22; H, 3.03; N, 21.08. Found: C, 54.18; H, 3.00; N, 20.99%.

##### 4.1.3.4. 7-Phenylethyl-1H-pyrrolo[3′,4′:5,6]pyrido[2,3-d]pyrimidine-2,4,6,8(3H,7H)-tetraone (28e)

T_1_ = 5 h, T_2_ = 17 h, washing solvent: H_2_O. **28e**: white solid (76 mg, 75% yield). mp: 359–361 °C. ^1^H NMR (300 MHz, DMSO-d_6_) *δ*: 12.47 (s, 1H), 11.85 (s, 1H), 8.46 (s, 1H), 7.15–7.32 (m, 5H), 3.84 (t, *J* = 6.9 Hz, 2H), 2.92 (t, *J* = 6.9 Hz, 2H) ppm. ^13^C NMR (75 MHz, DMSO-d_6_) *δ*: 165.1, 165.0, 161.5, 157.0, 155.8, 150.0, 138.2, 131.3, 128.7, 128.5, 126.5, 121.0, 112.8, 39.0, 33.6 ppm. IR *ν*: 3604, 3478, 3164, 3028, 3055, 2817, 1778, 1740, 1697, 1674, 1604, 1540, 1497, 1466, 1455, 1437, 1398, 1383, 1338, 1287, 1241, 1188, 1150, 1116, 1107, 1040, 1028, 987, 905, 866, 807 cm^−1^. Anal. Calcd. for C_17_H_12_N_4_O_4_ · 0.75 H_2_O (349.81): C, 58.37; H, 3.89; N, 16.02. Found: C, 58.48; H, 3.89; N, 16.13%.

##### 4.1.3.5. 7-(3-(Dimethylamino)propyl)-1H-pyrrolo[3′,4′:5,6]pyrido [2,3-d]pyrimidine-2,4,6,8 (3H,7H)-tetraone, hydrochloride (28h)

T_1_ = 2 h, T_2_ = overnight, washing solvent: EtOH. The solid was then stirred in EtOH (10 mL) with bubbling HCl gas during few seconds and the resulting precipitate was isolated by filtration, washed with EtOH and Et_2_O to afford pure **28h** as a white solid (67 mg, 63% yield). mp: 309–311 °C. ^1^H NMR (400 MHz, DMSO-d_6_) *δ*: 12.47 (s, 1H), 11.85 (s, 1H), 10.29 (s, 1H), 8.50 (s, 1H), 3.69 (t, *J* = 6.5 Hz, 2H), 3.15–3.05 (m, 2H), 2.71 (s, 6H), 2.08–1.96 (m, 2H) ppm. ^13^C NMR (100 MHz, DMSO-d_6_) *δ*: 165.3, 165.2, 161.4, 156.9, 156.1, 149.9, 131.2, 121.2, 112.5, 54.1, 42.0, 35.1, 23.0 ppm. IR *ν*: 3184, 3134, 3034, 2910, 2694, 1785, 1713, 1680, 1607, 1527, 1435, 1396, 1366, 1355, 1285, 1185, 1114, 1000, 886, 778, 768, 742, 692 cm^−1^. Anal. Calcd. for C_14_H_16_ClN_5_O_4_ · 0.75 H_2_O (367.27): C, 45.78; H, 4.80; N, 19.07. Found: C, 46.18; H, 4.49; N, 18.83%.

##### 4.1.3.6. N^6^,N^7^-Bis((2,6-dioxo-1,2,3,6-tetrahydropyrimidin-4-yl) methyl)-2,4-dioxo-1,2,3,4-tetrahydropyrido[2,3-d]pyrimidine-6,7-dicarboxamide (30)

To a suspension of **27** (232 mg, 1.00 mmol) in DMF (10 mL), Et_3_N (0.697 mL, 5.00 mmol, 5 eq.) and **29**^49^^,^^50^ (533 mg, 3.00 mmol, 3 eq.) were added and the mixture was heated at 90 °C overnight. Once cooled at r.t., the reaction mixture was poured on Et_2_O (10 mL) and maintained at 4 °C for 2 h. To a solution of the resulting solid in H_2_O (12 mL), a 1 N HCl solution was added until a pH < 3 was reached. The solid was filtrated, washed successively with H_2_O and EtOH to afford pure **30** as a white solid (410 mg, 82% yield). mp: 259–261 °C (decomposition). ^1^H NMR (400 MHz, DMSO-d_6_) *δ*: 12.10 (s, 1H), 11.70 (s, 1H), 10.97 (s, 2H), 10.90 (s, 1H), 10.82 (s, 1H), 9.10 (t, *J* = 5.8 Hz, 1H), 8.96 (t, *J* = 6.1 Hz, 1H), 8.58 (s, 1H), 5.53 (s, 1H), 5.48 (s, 1H), 4.18–4.07 (m, 4H) ppm. ^13^C NMR (100 MHz, DMSO-d_6_) *δ*: 166.0, 165.3, 164.0, 161.6, 156.9, 153.4, 153.3, 152.4, 151.4, 151.3, 150.3, 136.8, 124.6, 109.8, 96.8, 96.6, 39.3, 38.7 ppm. IR *ν*: 3563, 3489, 3247, 3118, 2988, 2819, 1689, 1657, 1609, 1583, 1527, 1502, 1419, 1357, 1321, 1284, 1233, 1020, 841, 819, 766, 743, 703 cm^−1^. Anal. Calcd. for C_19_H_15_N_9_O_8_ · 3.5 H_2_O (560.43): C, 40.72; H, 3.96; N, 22.49. Found: C, 40.97; H, 3.73; N, 22.02%.

### 4.2. Biologicals

A spectrophotometric assay method was adopted to evaluate *in vitro E. coli* TP inhibiting activity of all the synthesised compounds. The conversion of thymidine to thymine was recorded at 290 nm. Thymidine was dissolved in buffer (3 mM). DMSO stock solutions of the tested compounds were diluted in buffer, the incubation medium contained 2.5% of DMSO. All experiments were conducted in triplicate.

#### 4.2.1. In vitro thymidine phosphorylase essay

Initially, all synthesised compounds were tested against *E. coli* TP at 50 µM or at the maximum concentration allowed by their solubility.

The method reported by Khan et al.^51^ has been adapted to a screening in 96-well plates (200 µL well, UV-Star^®^ 96-well microplate, Greiner). Each well contained 160 µL of 50 mM KH_2_PO_4_ buffer (pH 7.4), 0.006 U of *E. coli* TP (T2807-1KU, Sigma Aldrich), and 20 µL of the tested compound solution. The reaction was initiated by the addition of 20 µL thymidine (i.e. a 300 µM final concentration corresponding to the Km^52^). The decrease in absorbance due to conversion of thymidine to thymine was followed during 30 min (PowerWave HT Microplate Reader, Biotek) at 25 °C, with one measure every 2 min and a 30 s shaking before each measure. Data were processed using Biotek KC4 software and the results were expressed in percentage of inhibition at 50 µM or at the maximum concentration allowed by the solubility of the tested compound (Tables 2–4). For the most active and soluble compounds **2d**, **2l**, **2p**, **28a**, and **30** (inhibition > 30% and solubility > 50 µM), IC_50_ values have been determined.

#### 4.2.2. Phosphate competition study

For this study, the reaction mixture was composed of 160 µL of 10 mM Tris buffer (pH 7.4), 150 mM NaCl, 2 mM EDTA, 0.006 U of *E. coli* TP, different concentrations of KH_2_PO_4_ (2, 5, 10 and 30 mM). Compounds were tested at a concentration close to their IC_50_. The reaction was initiated by the addition of 20 µL of thymidine (3 mM and 10 mM solutions).

#### 4.2.3. Enzyme inhibition kinetic study

The TP inhibiting activity at differents concentrations of compound **2d** (0, 15, 20, 30 µM) and **2p** (0, 4, 6, 8 µM) was evaluated in the presence of different concentrations of thymidine (200, 250, 300, 500, 750, 1000 mM) in KH_2_PO_4_ buffer (pH 7.4).

#### 4.2.4. In vitro anti-proliferative assay

Cell viability was determined on HUVEC and A431 cell lines using 3–(4,5-dimethylthiazol-2-yl)-2,5-diphenyltetrazolium bromide (MTT; Sigma-Aldrich) assay^53^ after treatment with compounds **2d** and **2p**. A total of 5 × 10^3^ cells/well (A431 cell line) or 10^4^ cells/well (HUVEC cell line) were plated in a 96-well plate and treated, in duplicates, with compounds **2d** and **2p** at five concentrations (0.1 µM, 1 μM, 10 μM, 50 μM, 100 μM) for 72 h. Absorbance was read at 570 nm using Labsystem Multiskan MS microplate reader. The results were expressed as percentages of growth inhibition compared to positive control cell growth (100%).

### 4.3. Molecular docking study

Molecular modelling studies were carried out with the GOLD software (version 5.1). The ligands were constructed using the standard fragments of the Sybyl library 6.9.1. Their geometry was optimised using the Tripos force field by assigning partial loads calculated by the Gasteiger–Hückel method to a gradient of 0.0001 Kcal/mol/Å.

The conformations obtained for each compound were classified by a consensus scoring function and then the conformation groups were observed visually to evaluate their consistency and their complementarity with the active site. Only the most representative conformations of each group were selected.

In order to check the validity of the results, the **TPI** reference inhibitor was relocated to the hTP binding site (PDB code: 1UOU) following the same protocol as for the inhibitors. The best solution has an RMS on heavy atoms of 0.654 Å with respect to the 1UOU crystallographic structure. Results were visualised by Chimaera and Maestro.

Supplementary data includes docking superposition of compounds **2d**, **2p**, and **2l** in the hTP active site; two docked conformations of compound **2c** in the hTP active site; docking superposition of compounds **2i**, and **23b** in the hTP active site; NMR spectra of the final compounds.

## Supplementary Material

Supplemental Material
