# Supplementary material for: Polycyclic nitrogen heterocycles as potential thymidine phosphorylase inhibitors: synthesis, biological evaluation, and molecular docking study
Source: J Enzyme Inhib Med Chem. 2021 Dec 22;37(1):252–68. doi: 10.1080/14756366.2021.2001806 (PMC8725971; doi:10.1080/14756366.2021.2001806)
Supplement: Supplemental Material [file IENZ_A_2001806_SM4166.pdf]

SupplementaryMaterial

Polycyclic Nitrogen Heterocycles as potential Thymidine Phosphorylase Inhibitors: Synthesis, biological Evaluation and molecular Docking Study

Karen Aknin[a], Alexis Bontemps[a], Amaury Farce[b], Eric Merlet[a], Philippe Belmont[a], Philippe Helissey[a], Philippe Chavatte[b], Marie-Agnès Sari[c], Sylviane Giorgi-Renault[a*], Stéphanie Desbène-Finck[a]*

[a] Cibles Thérapeutiques et Conception de Médicaments (CiTCoM), CNRS UMR8038, Faculté de Santé, Faculté de Pharmacie de Paris, Université de Paris, Paris, France; [b] Inserm, CHU Lille, U1286 - INFINITE - Institute for Translational Research in Inflammation, Université de Lille, France;[c] CNRS, UMR 8601, Laboratoire de Chimie et Biochimie Pharmacologiques et Toxicologiques, Faculté des Sciences, Université de Paris, Paris, France.

Contents

Page 2: **Figure S1.** Docking superposition of compounds **2d** (in blue), **2p** (in mauve), and **2l** (in yellow) in the hTP active site.

Page 3: **Figure S2.** Two docked conformations of compound **2c** in the hTP active site.

Page 4: **Figure S3.** Docking superposition of compounds **2i** (in green, series A) and **23b** (in black, series C) in the hTP active site.

Page 5- 42: **NMR** spectra of the final compounds

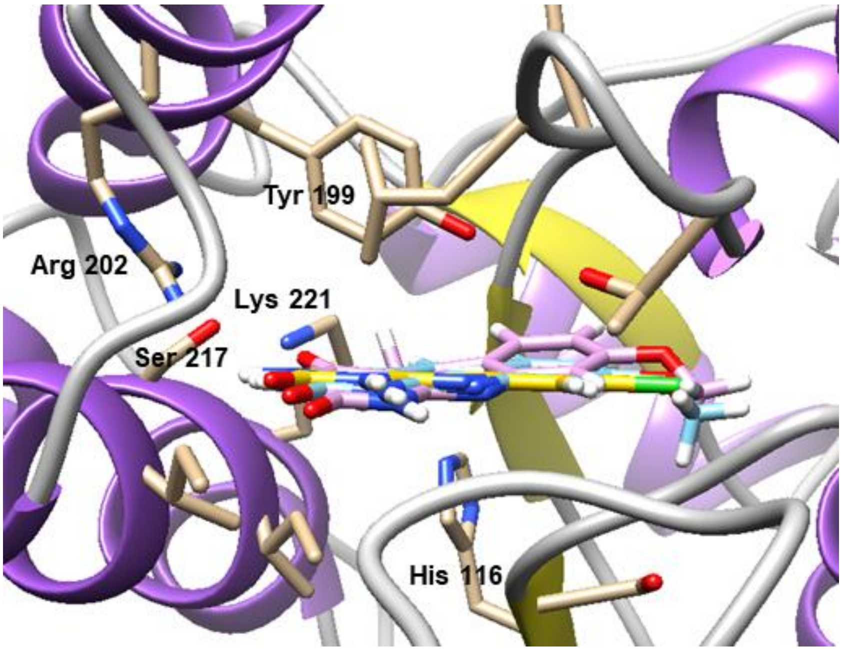

Figure S1. Docking superposition of compounds **2d** (in blue), **2p** (in mauve), and **2l** (in yellow) in the hTP active site.

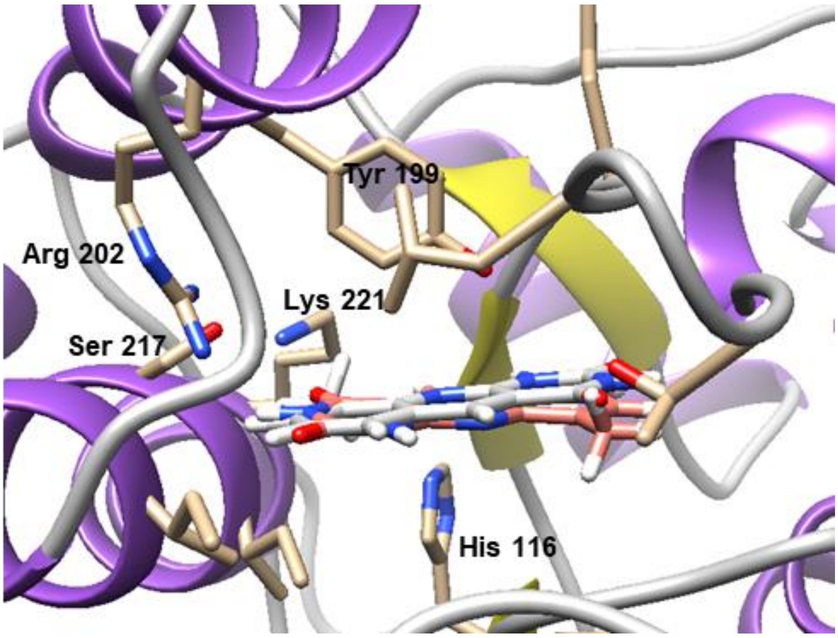

Figure S2. Two docked conformations of compound **2c** in the hTP active site.

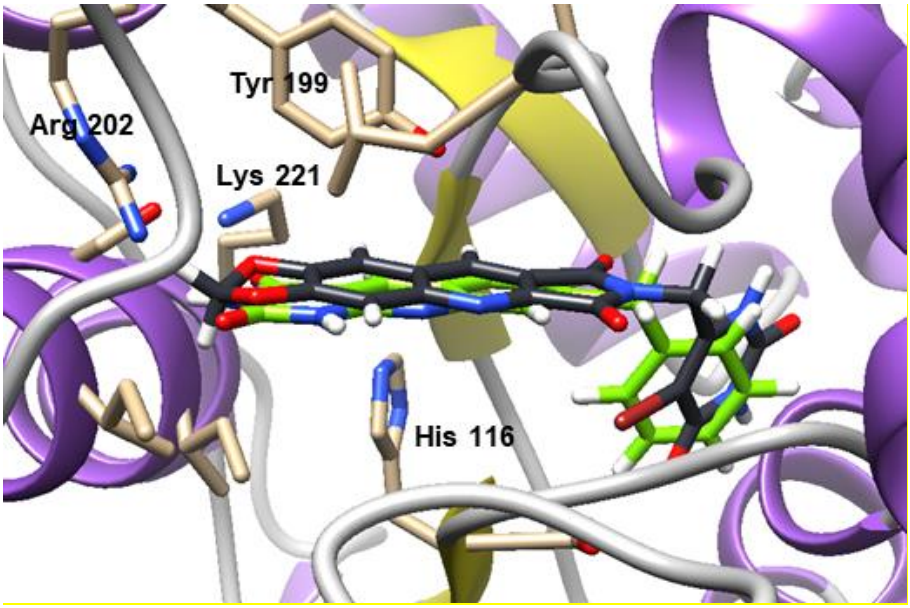

Figure S3. Docking superposition of compounds **2i** (in green, series A) and **23b** (in black, series C) in the hTP active site.

Compound 2e

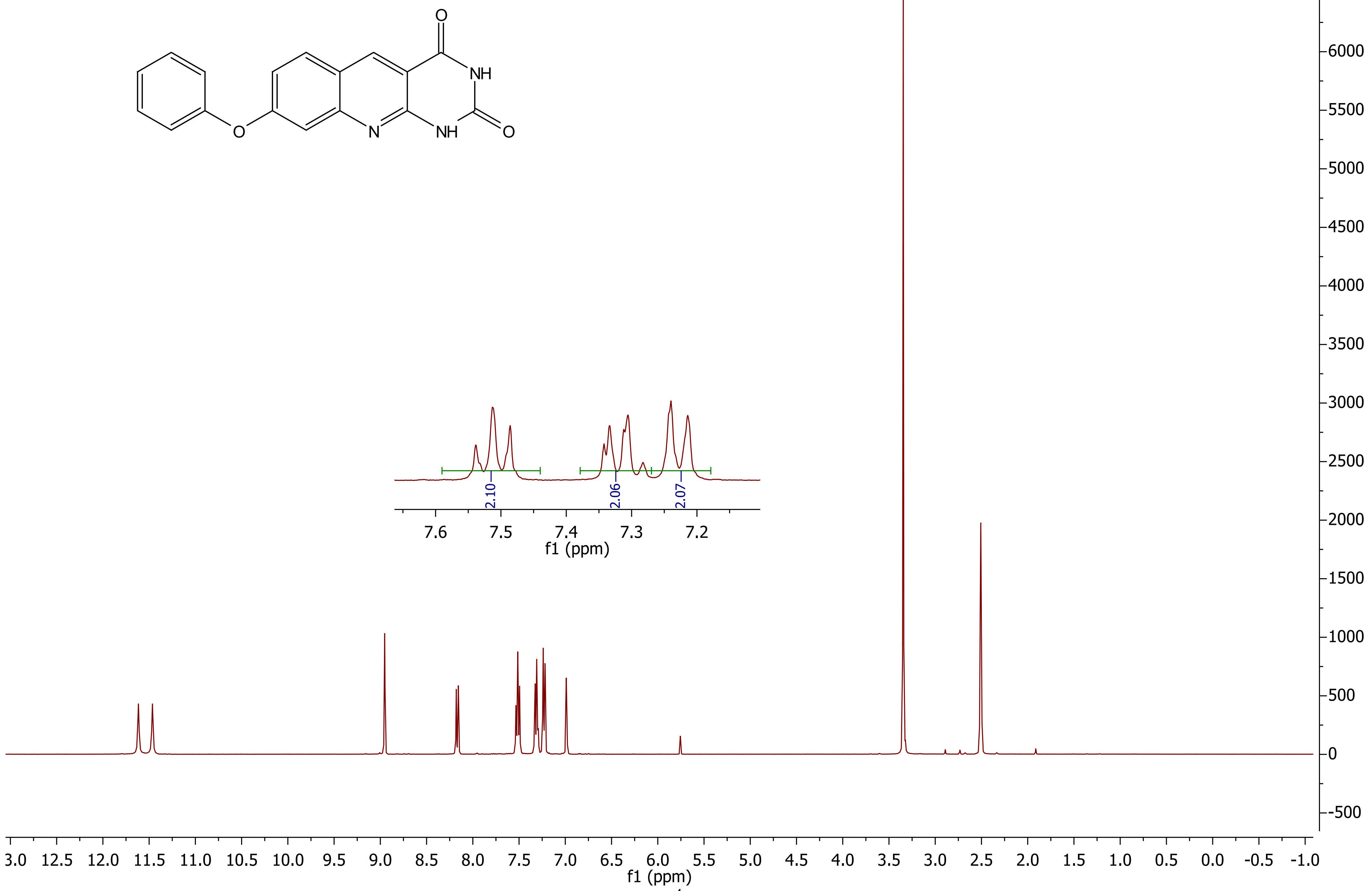
O
NH
N
NH
O
O
2.10
2.06
2.07
7.6 7.5 7.4 7.3 7.2
f1 (ppm)
7000 6500 6000 5500 5000 4500 4000 3500 3000 2500 2000 1500 1000 500 0 -500
3.0 12.5 12.0 11.5 11.0 10.5 10.0 9.5 9.0 8.5 8.0 7.5 7.0 6.5 6.0 5.5 5.0 4.5 4.0 3.5 3.0 2.5 2.0 1.5 1.0 0.5 0.0 -0.5 -1.0
f1 (ppm)

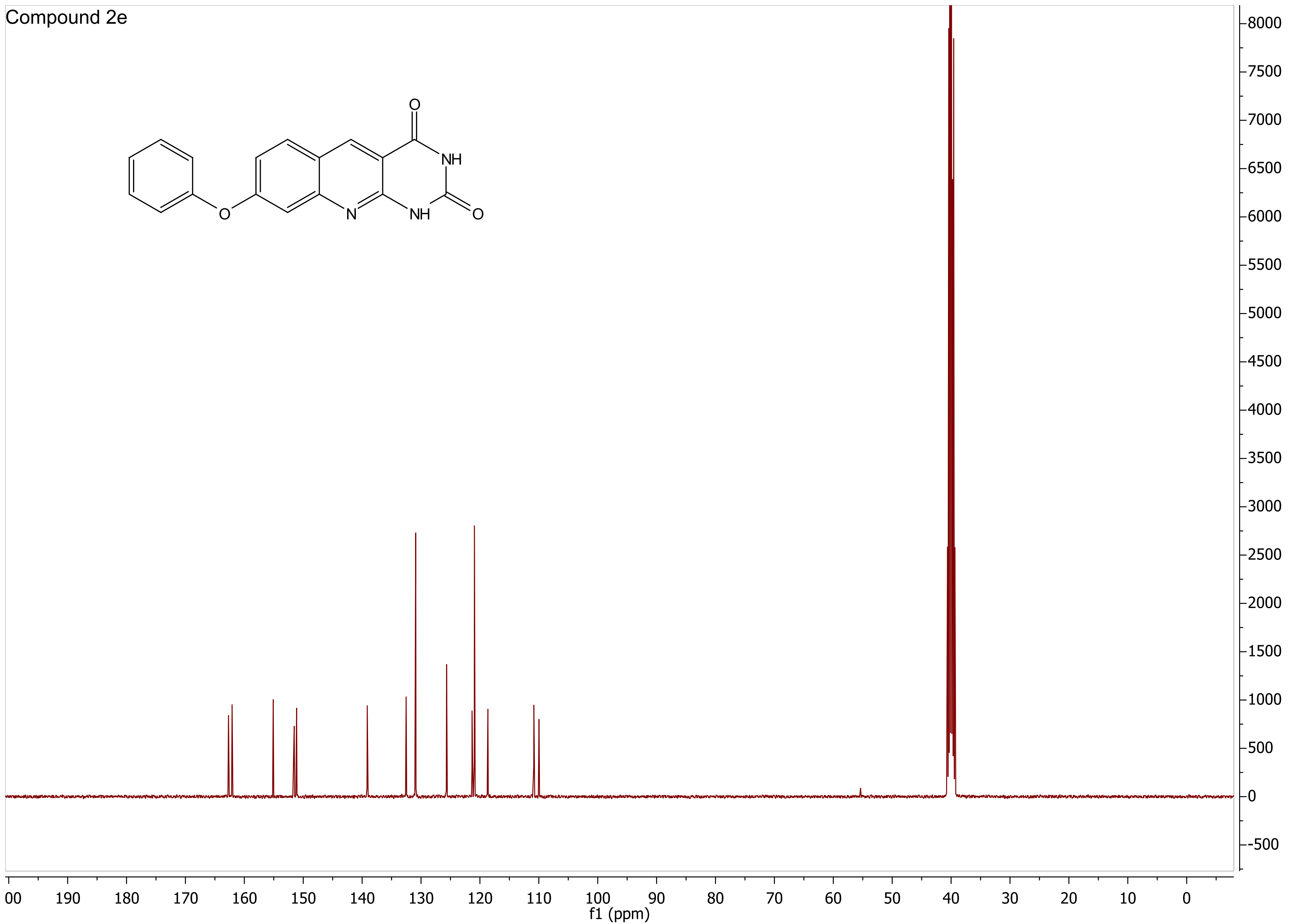
Compound 2e
O
NH
O
N
NH
O
f1 (ppm)

Compound 2i

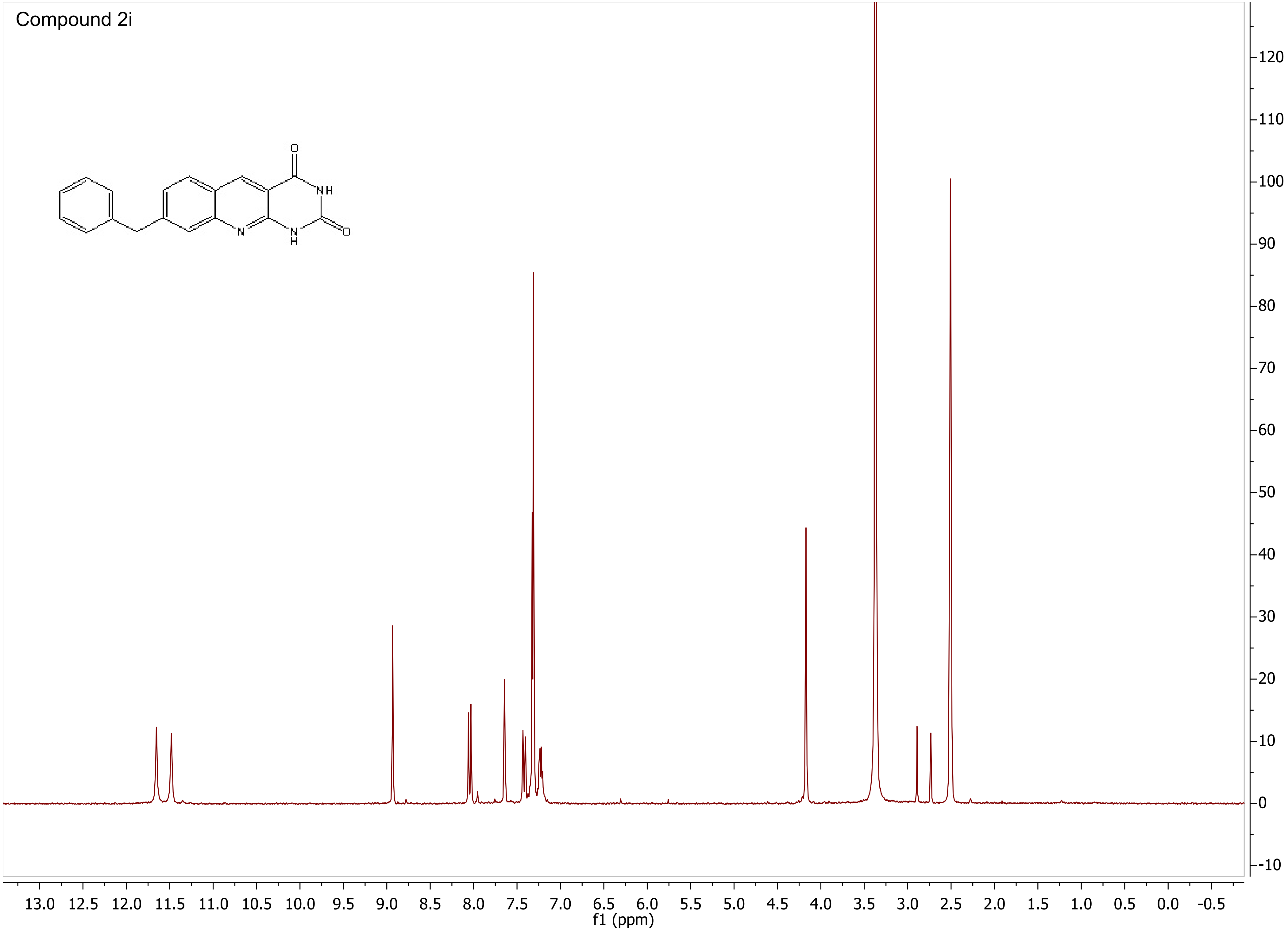

120
110
100
90
80
70
60
50
40
30
20
10
0
-10
13.0 12.5 12.0 11.5 11.0 10.5 10.0 9.5 9.0 8.5 8.0 7.5 7.0 6.5 6.0 5.5 5.0 4.5 4.0 3.5 3.0 2.5 2.0 1.5 1.0 0.5 0.0 -0.5
f1 (ppm)

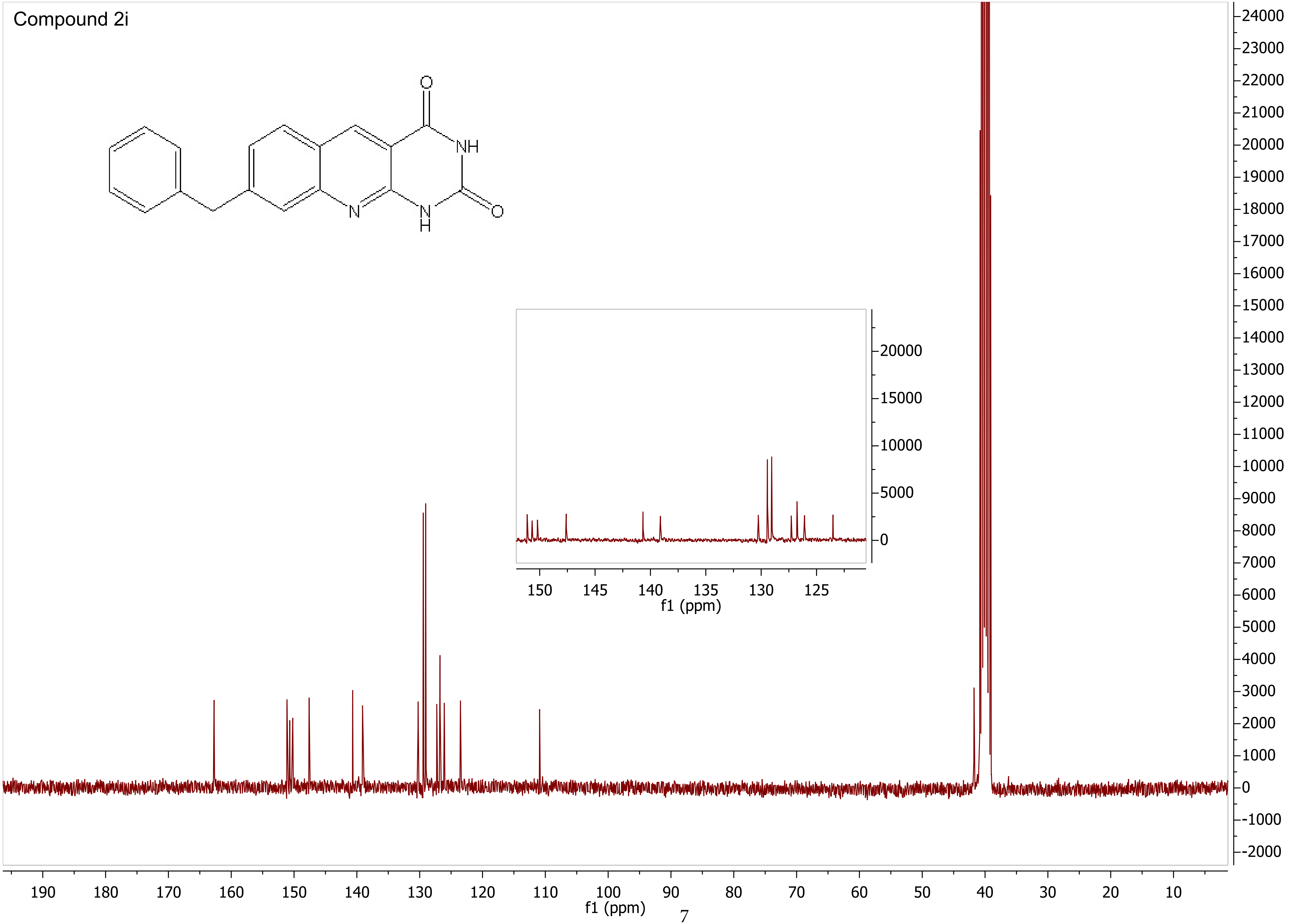

Compound 2i
O
NH
N
N
H
O
20000
15000
10000
5000
0
150
145
140
135
130
125
f1 (ppm)
24000
23000
22000
21000
20000
19000
18000
17000
16000
15000
14000
13000
12000
11000
10000
9000
8000
7000
6000
5000
4000
3000
2000
1000
0
-1000
-2000
190
180
170
160
150
140
130
120
110
100
90
80
70
60
50
40
30
20
10
f1 (ppm)

Compound 2l

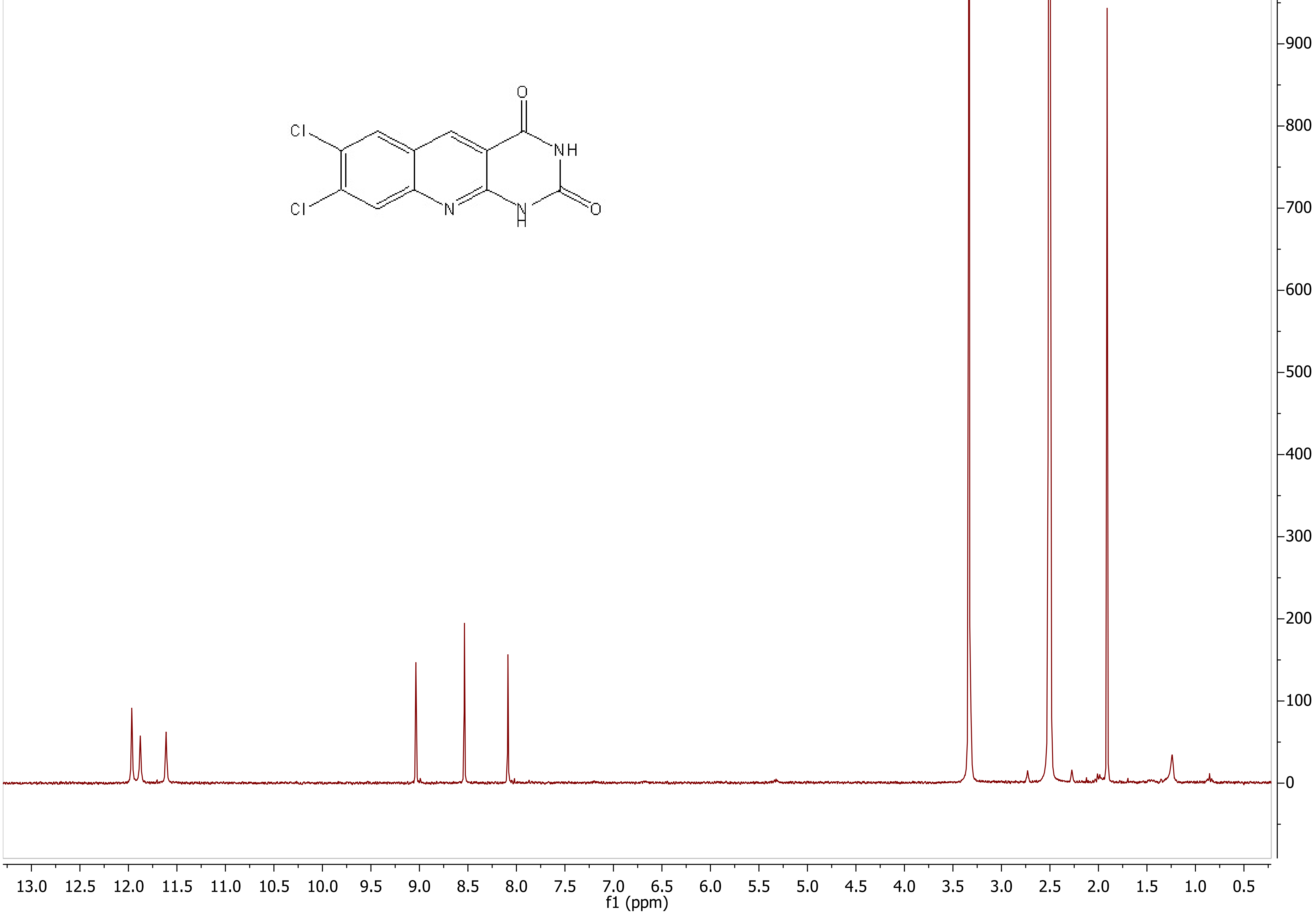

Cl
Cl
N
N
H
NH
O
O
13.0 12.5 12.0 11.5 11.0 10.5 10.0 9.5 9.0 8.5 8.0 7.5 7.0 6.5 6.0 5.5 5.0 4.5 4.0 3.5 3.0 2.5 2.0 1.5 1.0 0.5
f1 (ppm)
900
800
700
600
500
400
300
200
100
0

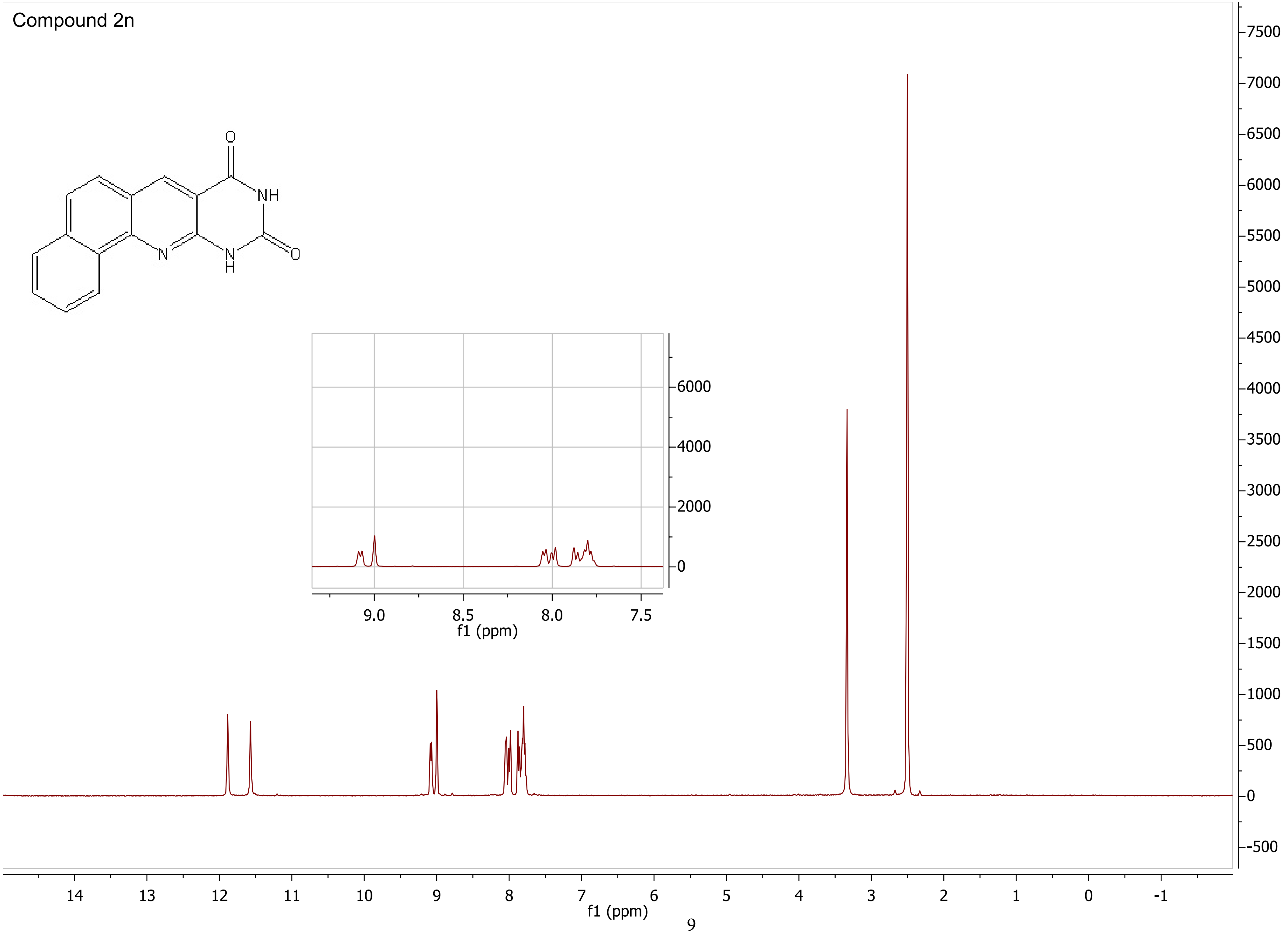
Compound 2n
O
NH
N
N
H
O
9.0
8.5
8.0
7.5
f1 (ppm)
6000
4000
2000
0
14
13
12
11
10
9
8
7
6
5
4
3
2
1
0
-1
f1 (ppm)
7500
7000
6500
6000
5500
5000
4500
4000
3500
3000
2500
2000
1500
1000
500
0
-500

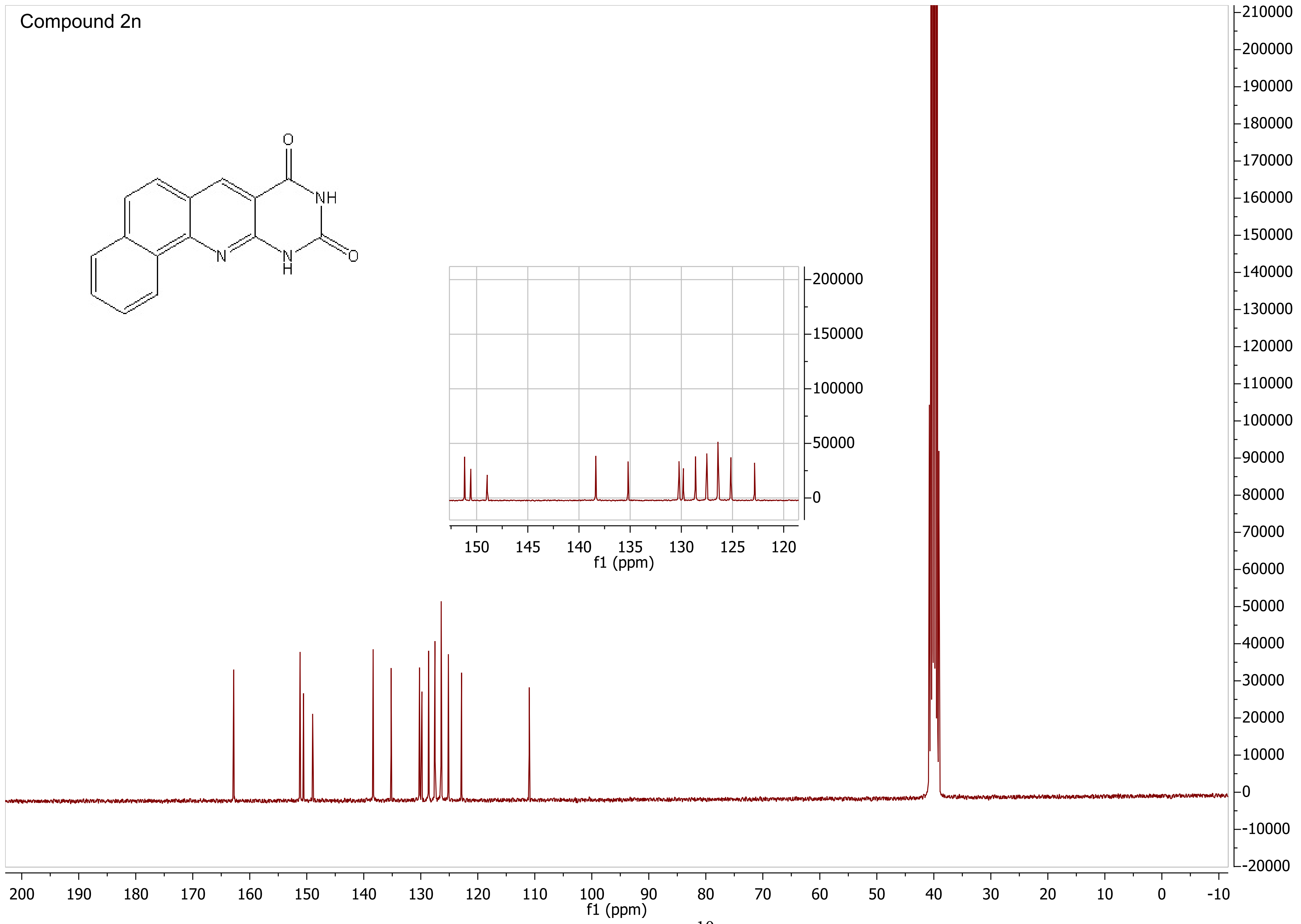
Compound 2n
O
NH
N
N
H
O
200000
150000
100000
50000
0
150 145 140 135 130 125 120
f1 (ppm)
210000
200000
190000
180000
170000
160000
150000
140000
130000
120000
110000
100000
90000
80000
70000
60000
50000
40000
30000
20000
10000
0
-10000
-20000
200 190 180 170 160 150 140 130 120 110 100 90 80 70 60 50 40 30 20 10 0 -10
f1 (ppm)

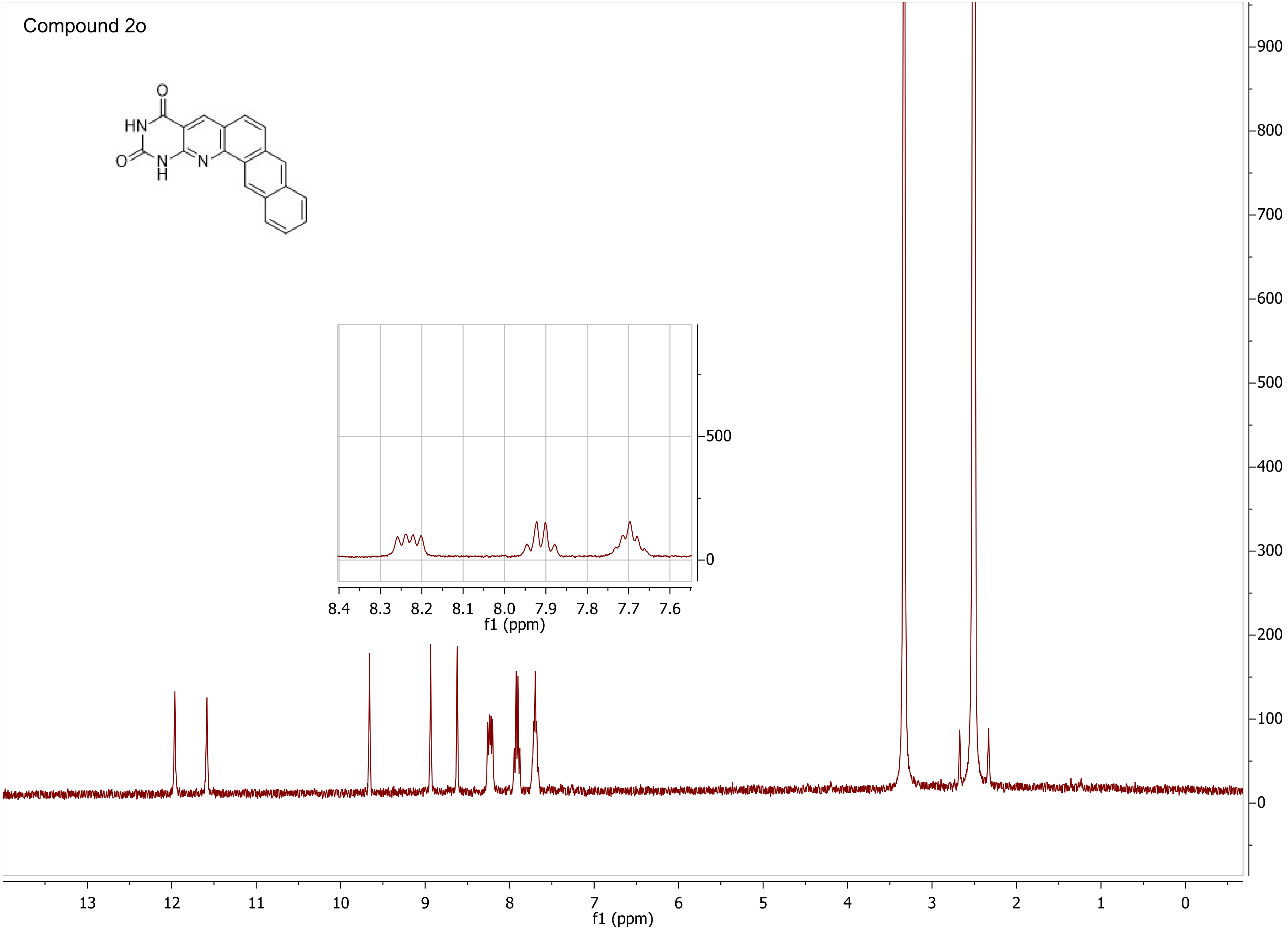

Compound 2o
HN
O
O
N
H
N
f1 (ppm)
f1 (ppm)

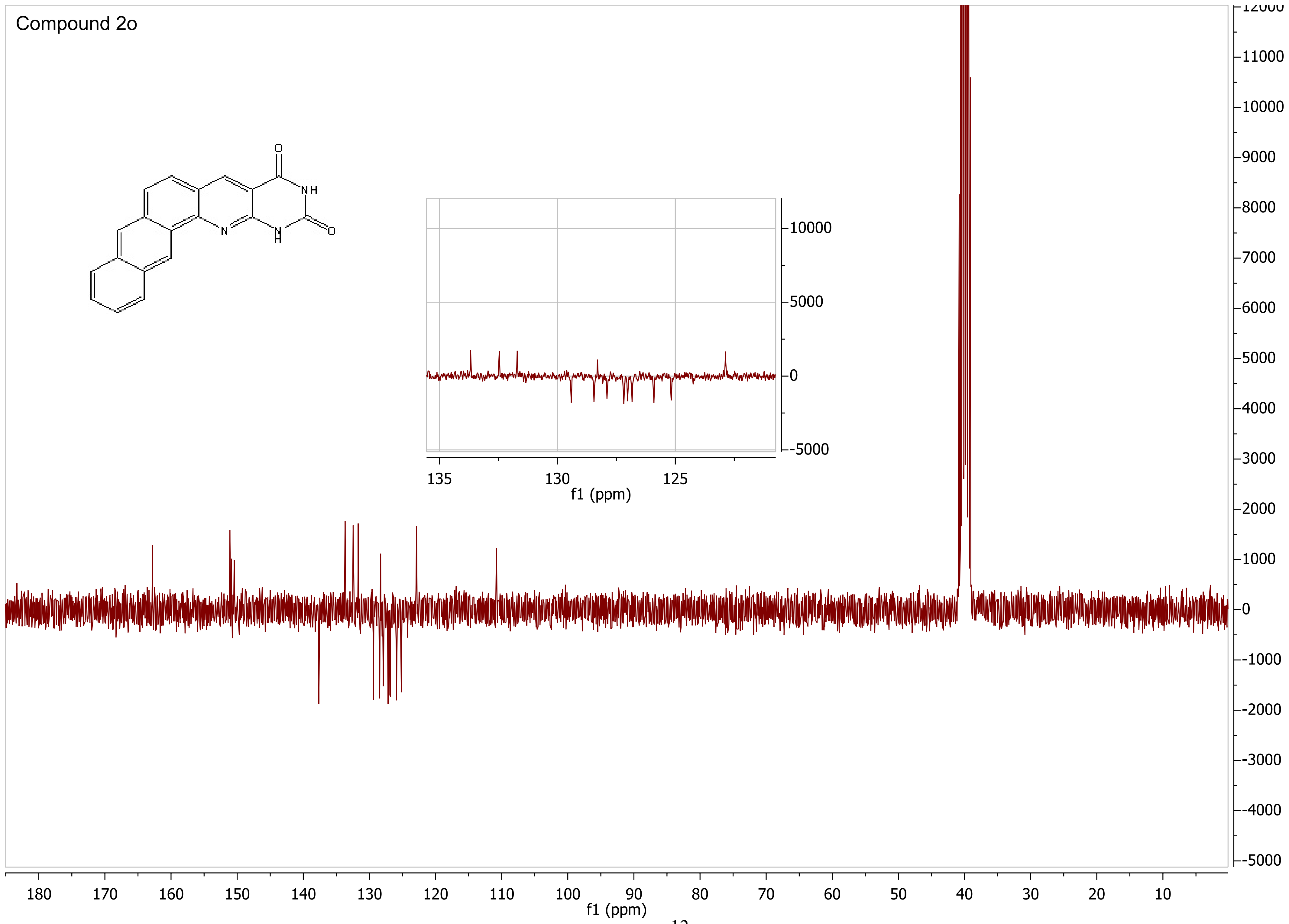
Compound 2o
f1 (ppm)
f1 (ppm)

Compound 2s

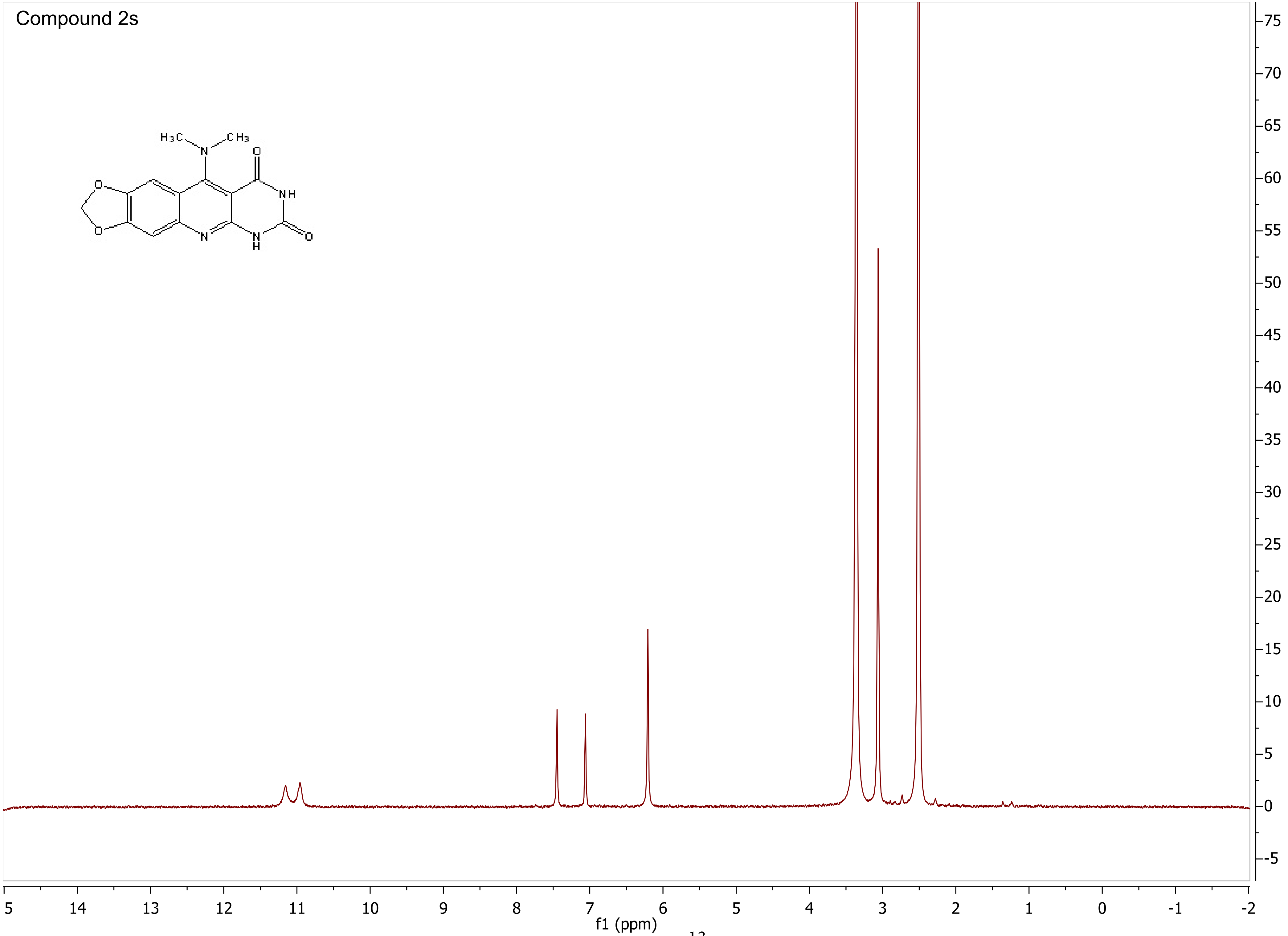

Compound 2s

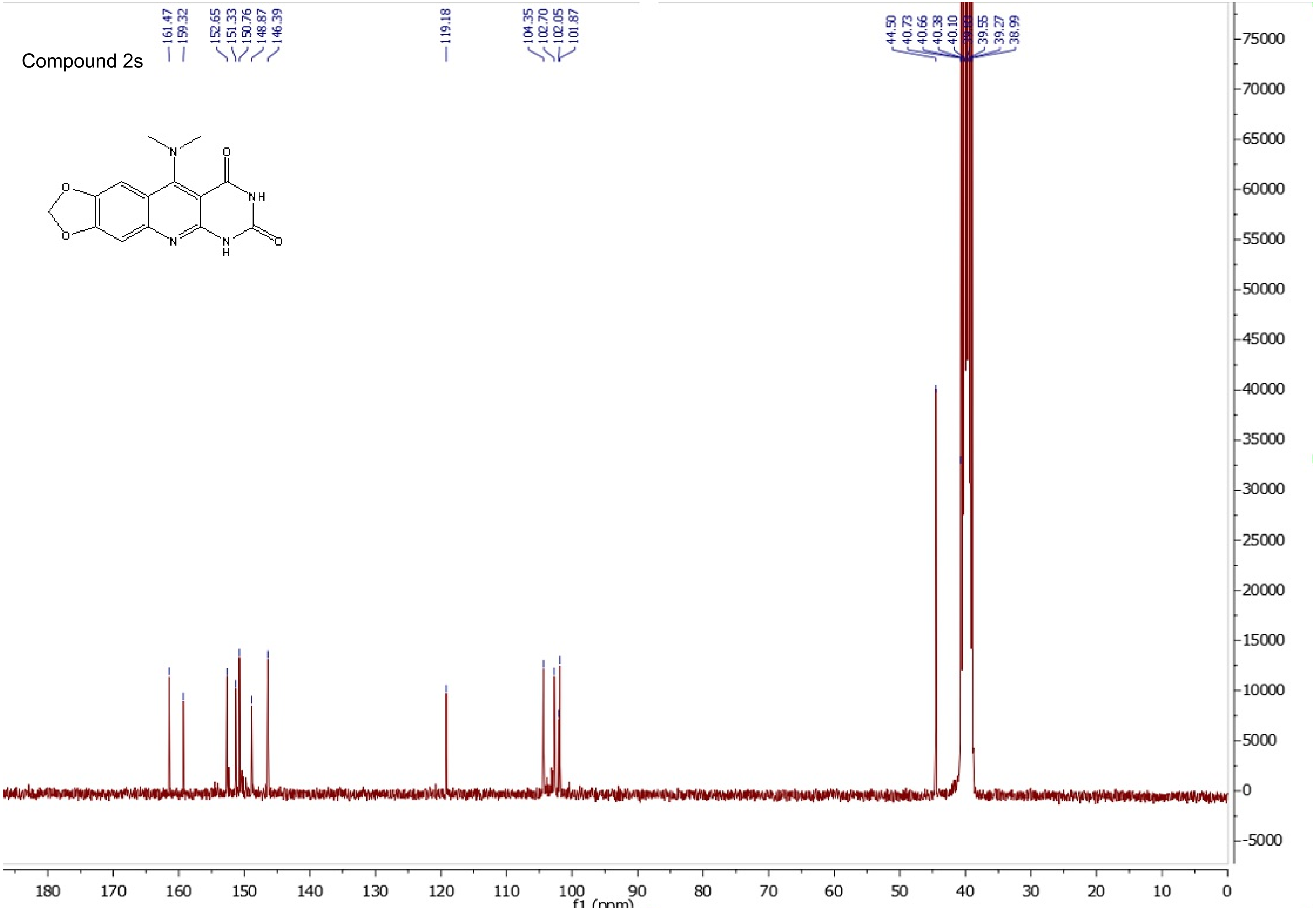
161.47
159.32
152.65
151.33
150.76
148.87
146.39
119.18
104.35
102.70
102.05
101.87
44.50
40.73
40.66
40.38
40.10
39.55
39.27
38.99
O
NH
O
N
H
N
O
O
N
f1 (ppm)

Compound 13a

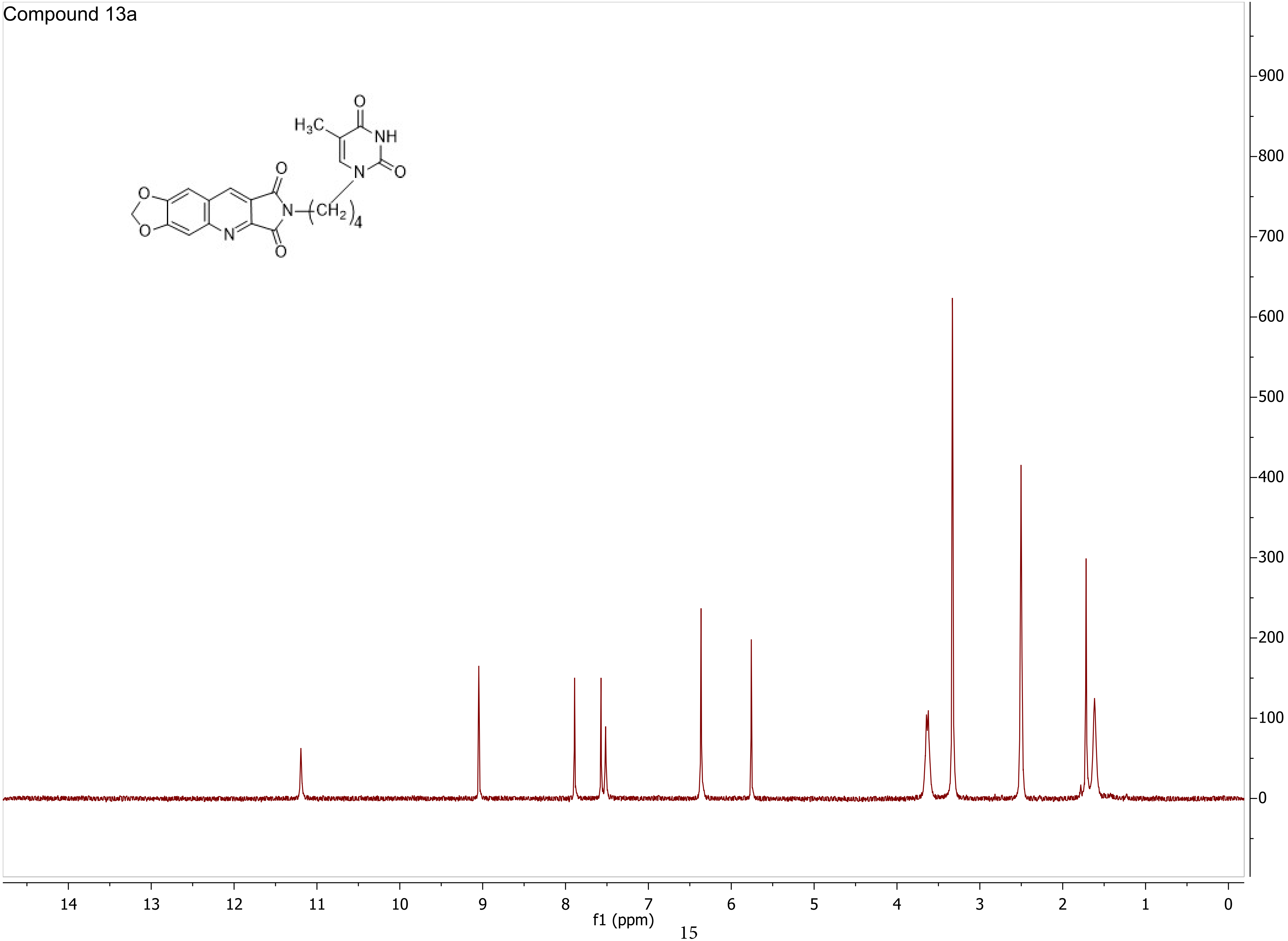

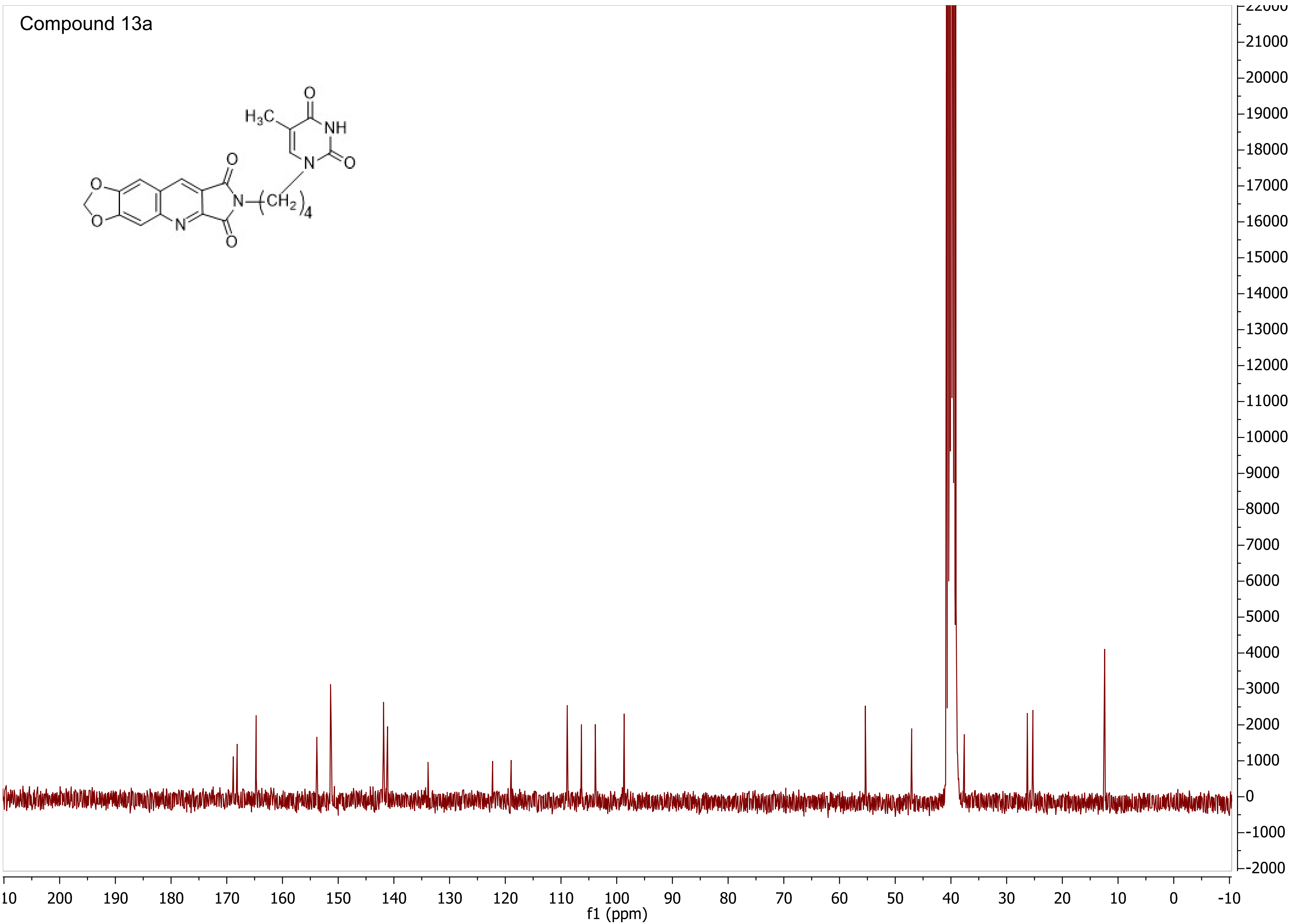
Compound 13a
f1 (ppm)

Compound 13b

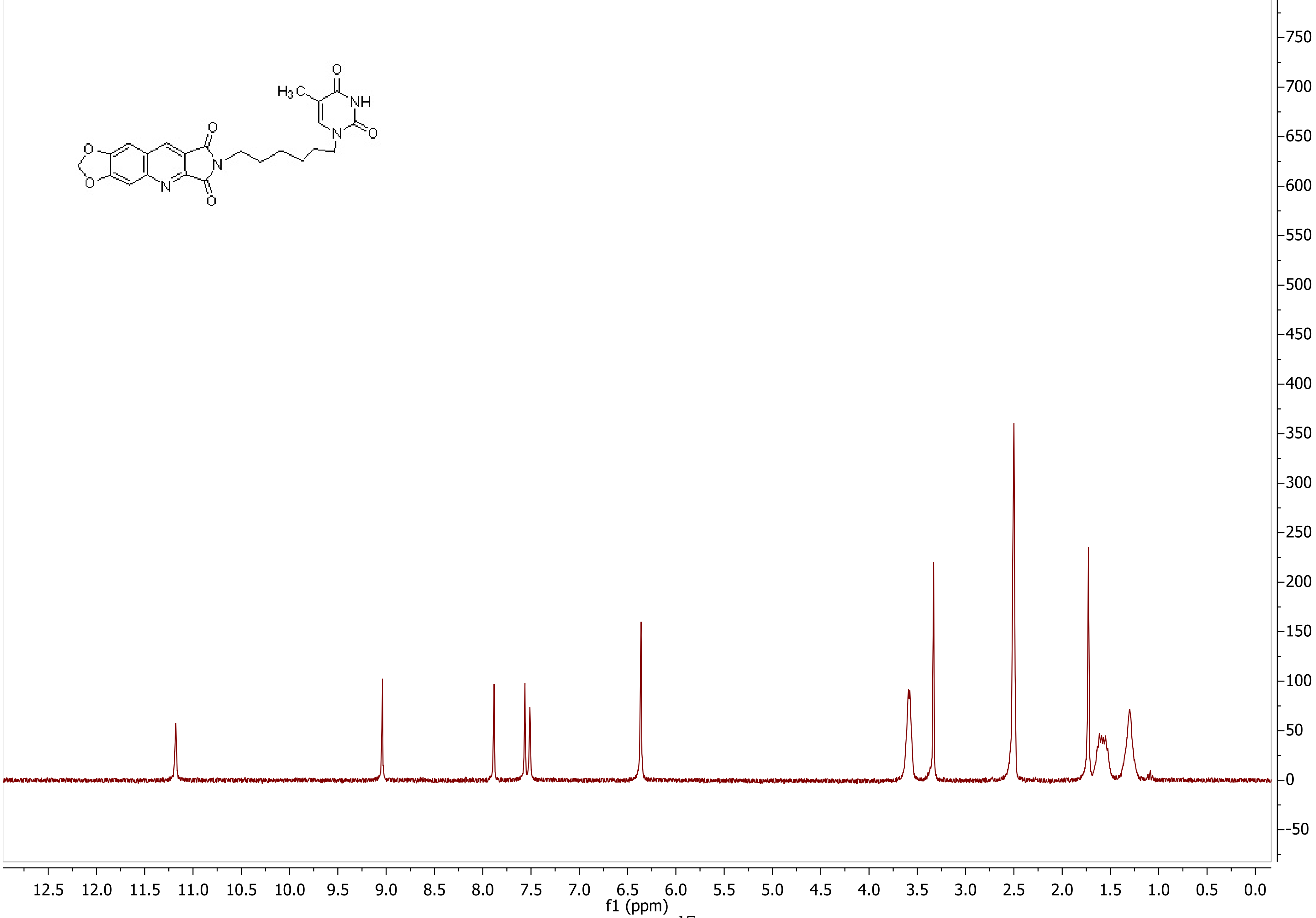

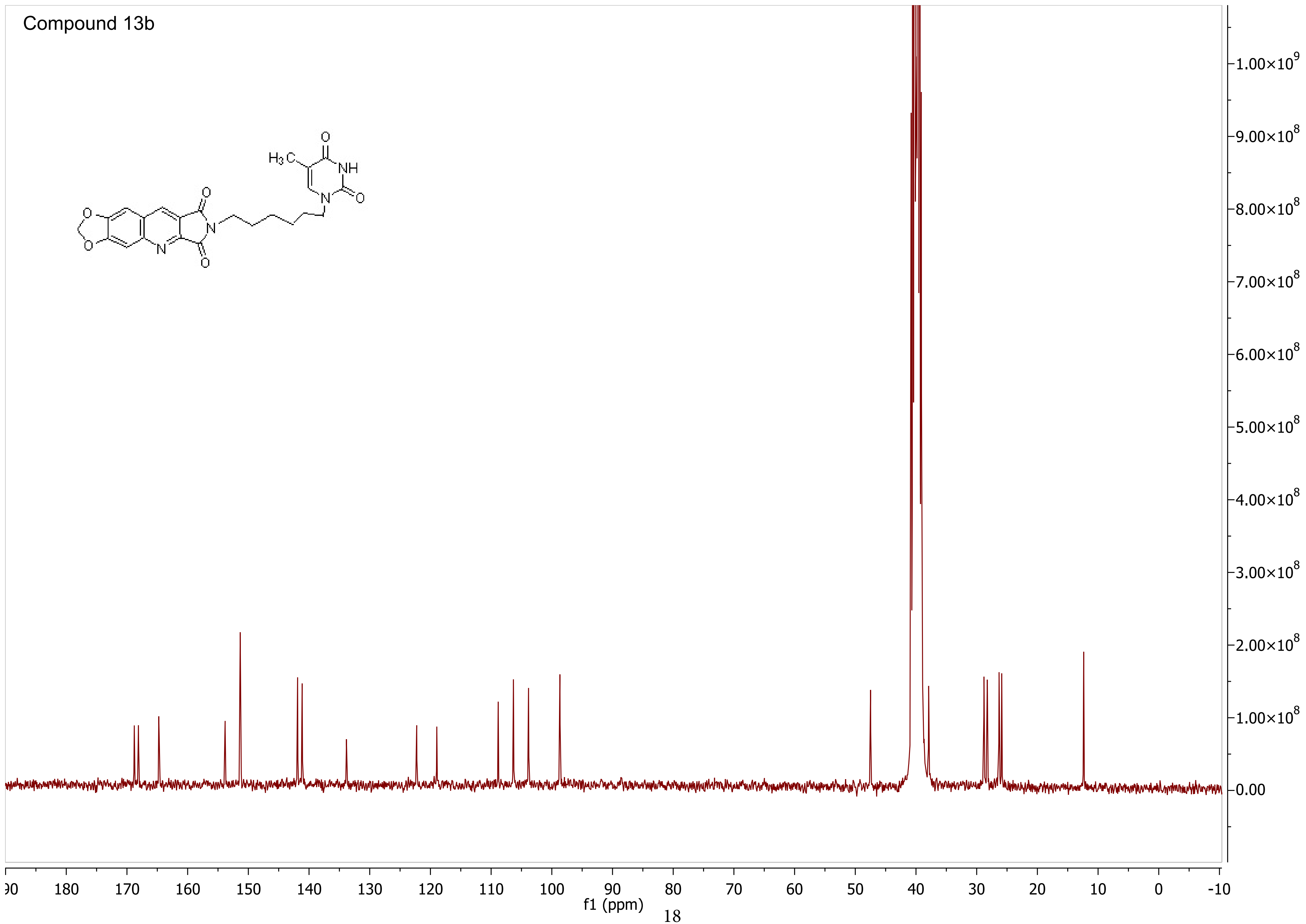
Compound 13b
f1 (ppm)
1.00×10^9
9.00×10^8
8.00×10^8
7.00×10^8
6.00×10^8
5.00×10^8
4.00×10^8
3.00×10^8
2.00×10^8
1.00×10^8
0.00
180 170 160 150 140 130 120 110 100 90 80 70 60 50 40 30 20 10 0 -10

Compound 16a

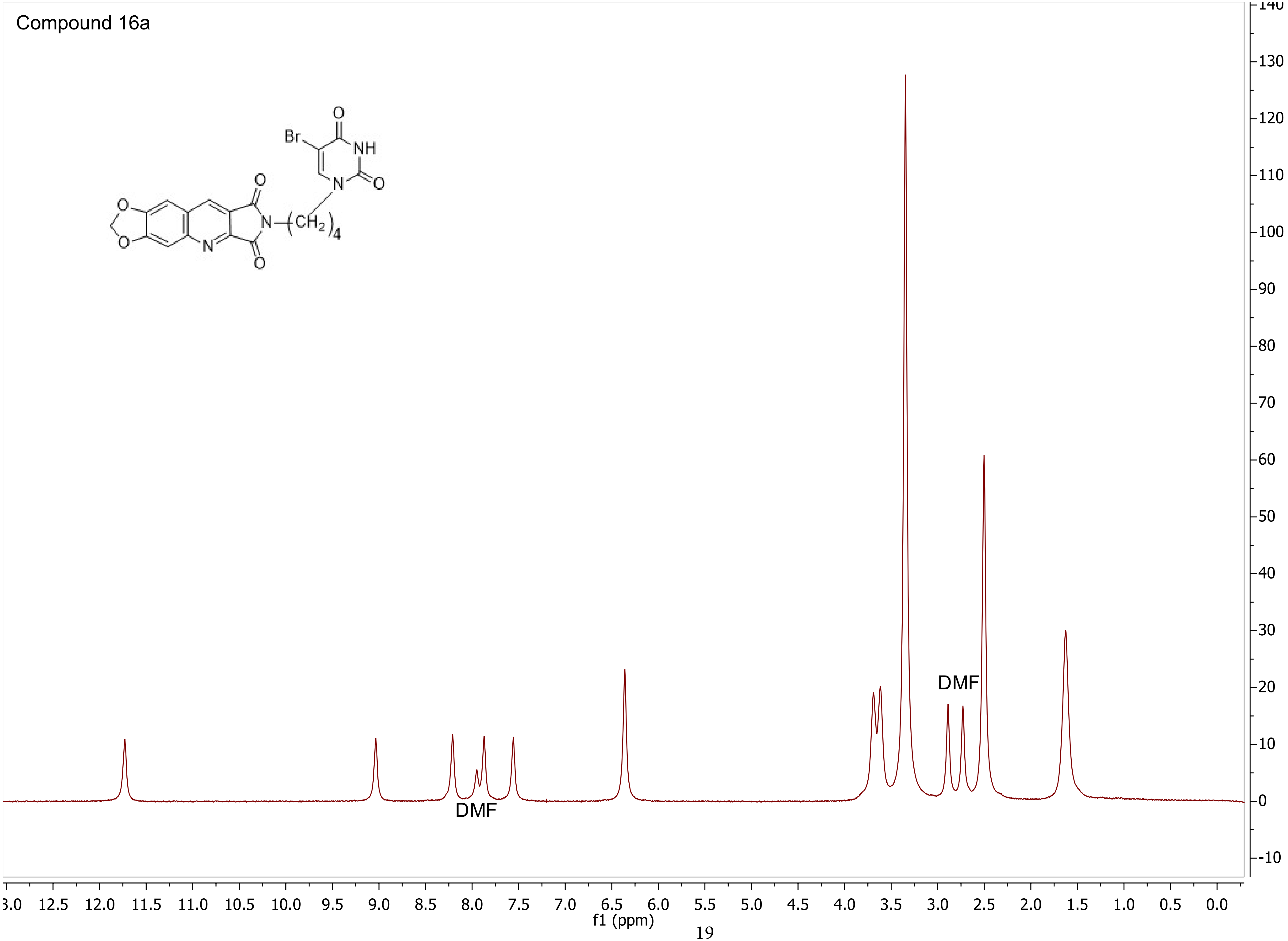

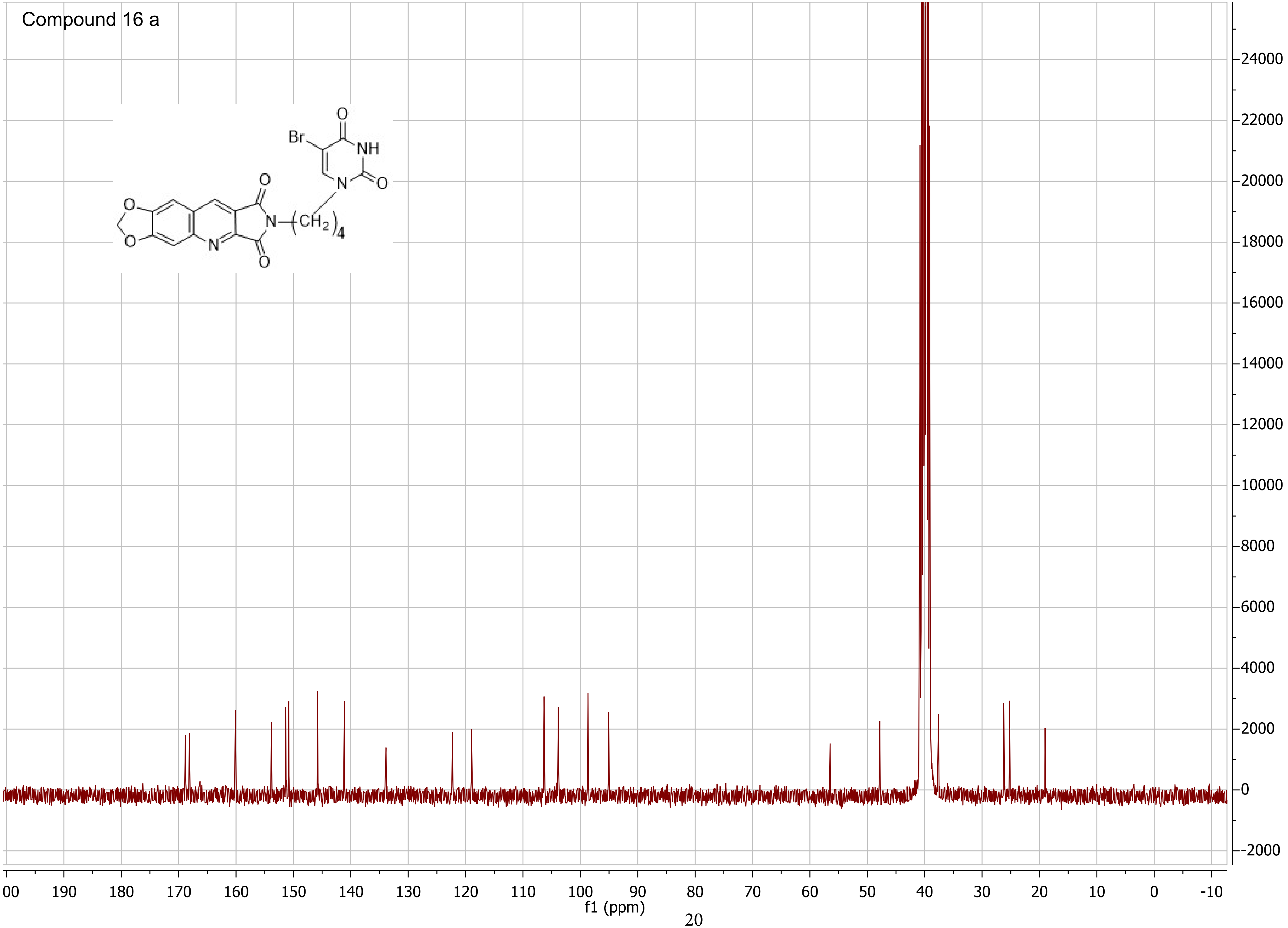
Compound 16 a
Br
O
NH
N
O
O
O
O
N
N
(CH2)4
O
f1 (ppm)
00
190
180
170
160
150
140
130
120
110
100
90
80
70
60
50
40
30
20
10
0
-10
24000
22000
20000
18000
16000
14000
12000
10000
8000
6000
4000
2000
0
-2000

Compound 16b

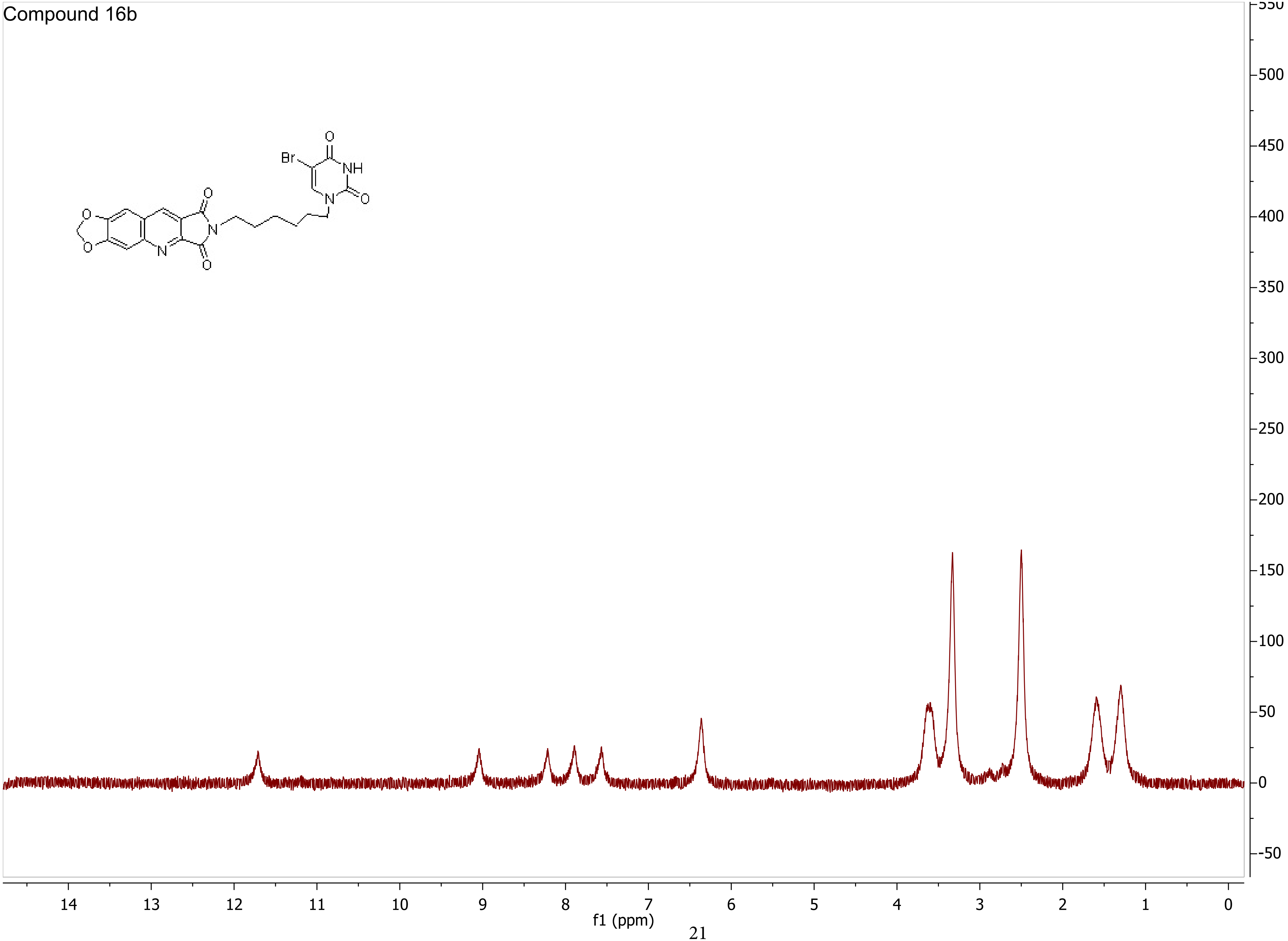

Compound 16b

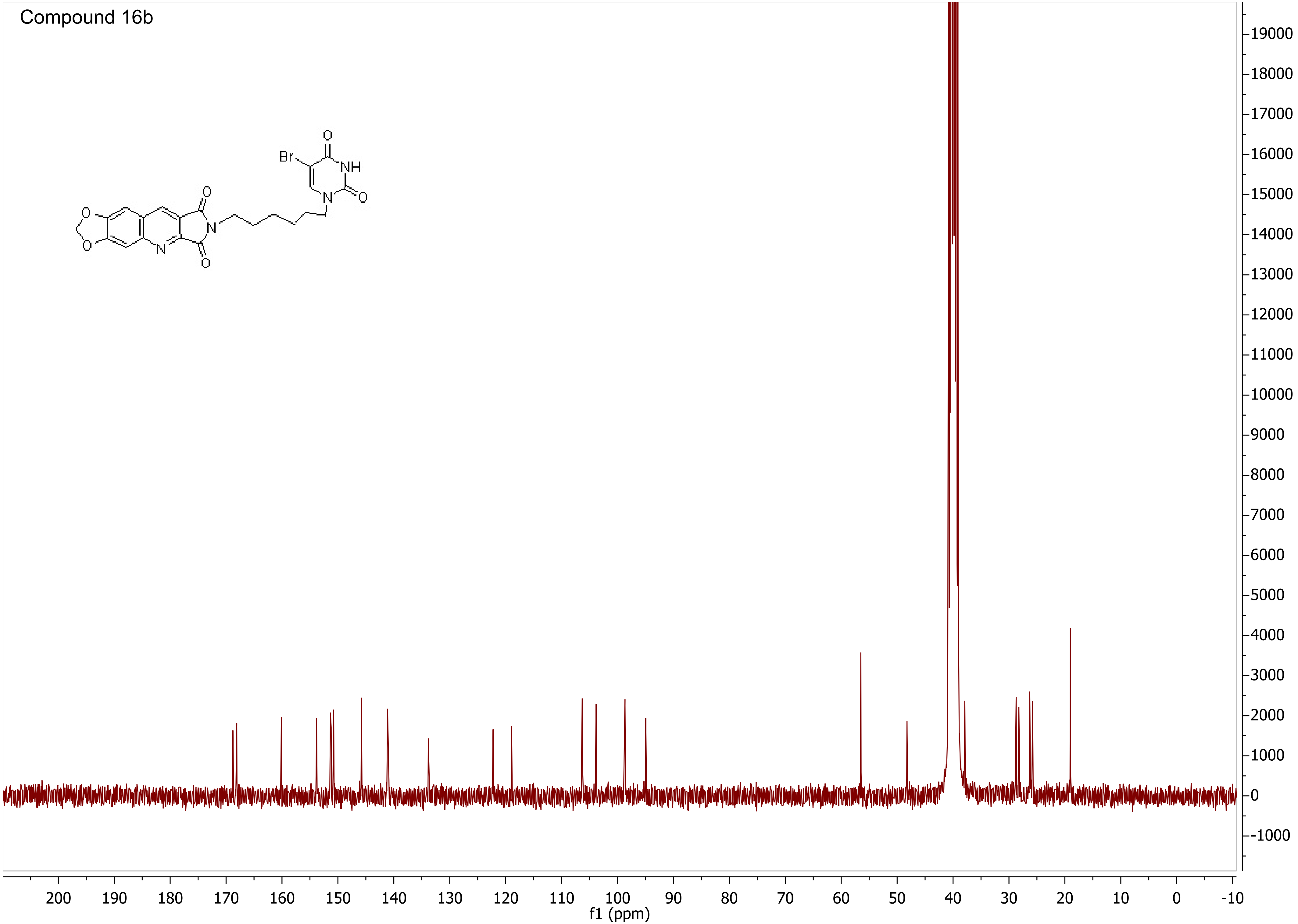

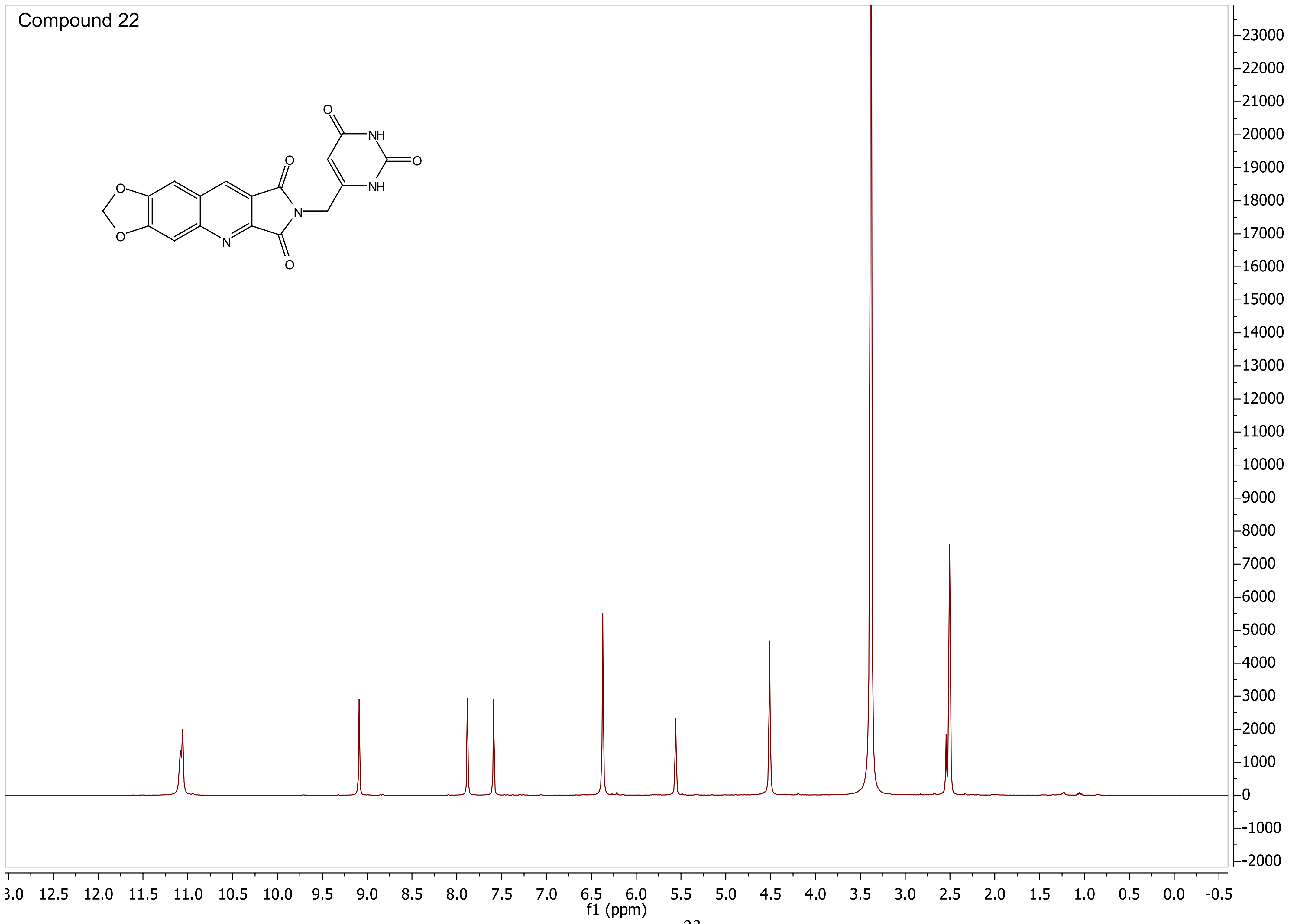
Compound 22
O
NH
O
NH
O
O
N
O
N
O
23000
22000
21000
20000
19000
18000
17000
16000
15000
14000
13000
12000
11000
10000
9000
8000
7000
6000
5000
4000
3000
2000
1000
0
-1000
-2000
3.0 12.5 12.0 11.5 11.0 10.5 10.0 9.5 9.0 8.5 8.0 7.5 7.0 6.5 6.0 5.5 5.0 4.5 4.0 3.5 3.0 2.5 2.0 1.5 1.0 0.5 0.0 -0.5
f1 (ppm)

Compound 22

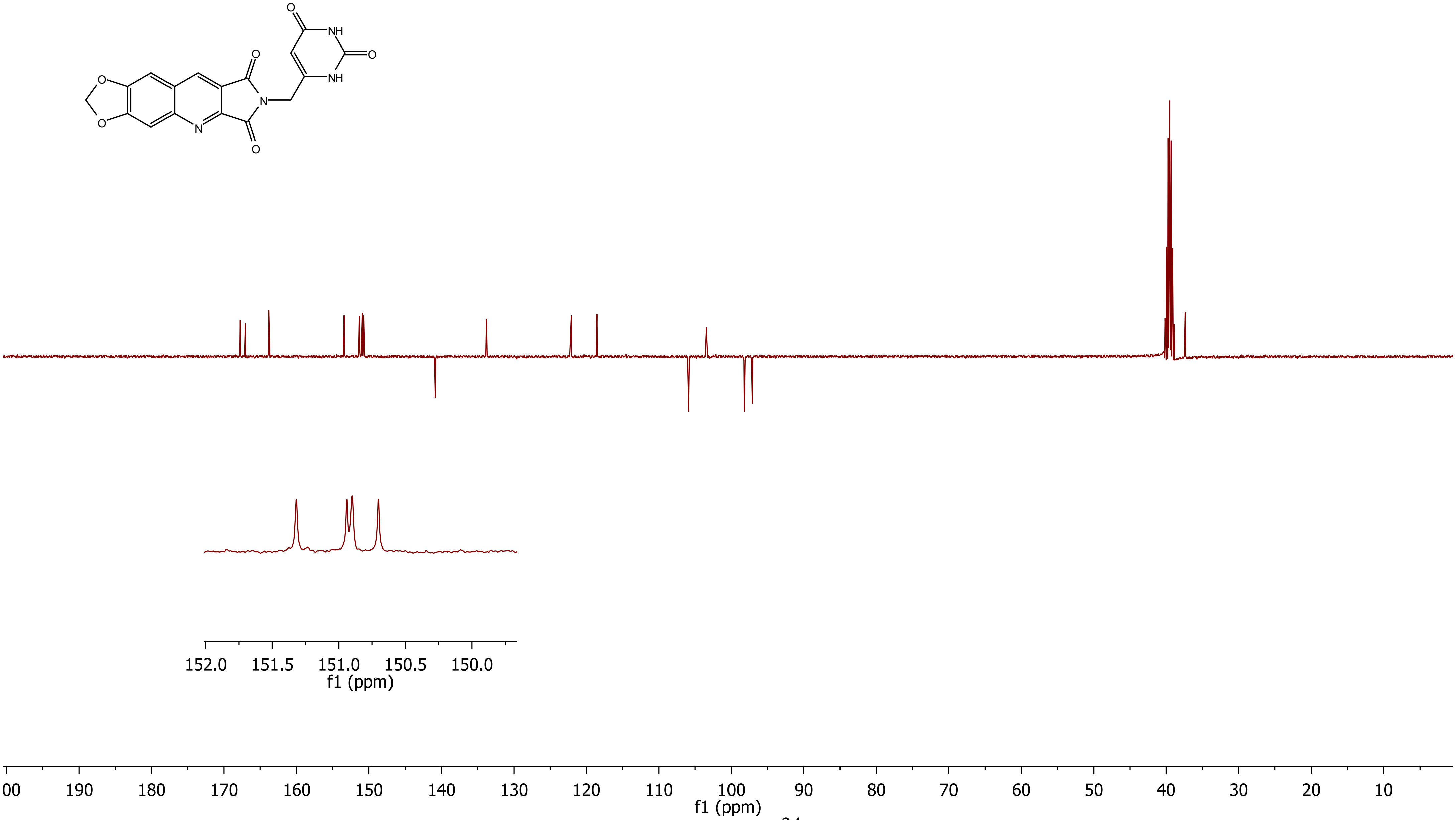
152.0 151.5 151.0 150.5 150.0
f1 (ppm)
00 190 180 170 160 150 140 130 120 110 100 90 80 70 60 50 40 30 20 10
f1 (ppm)

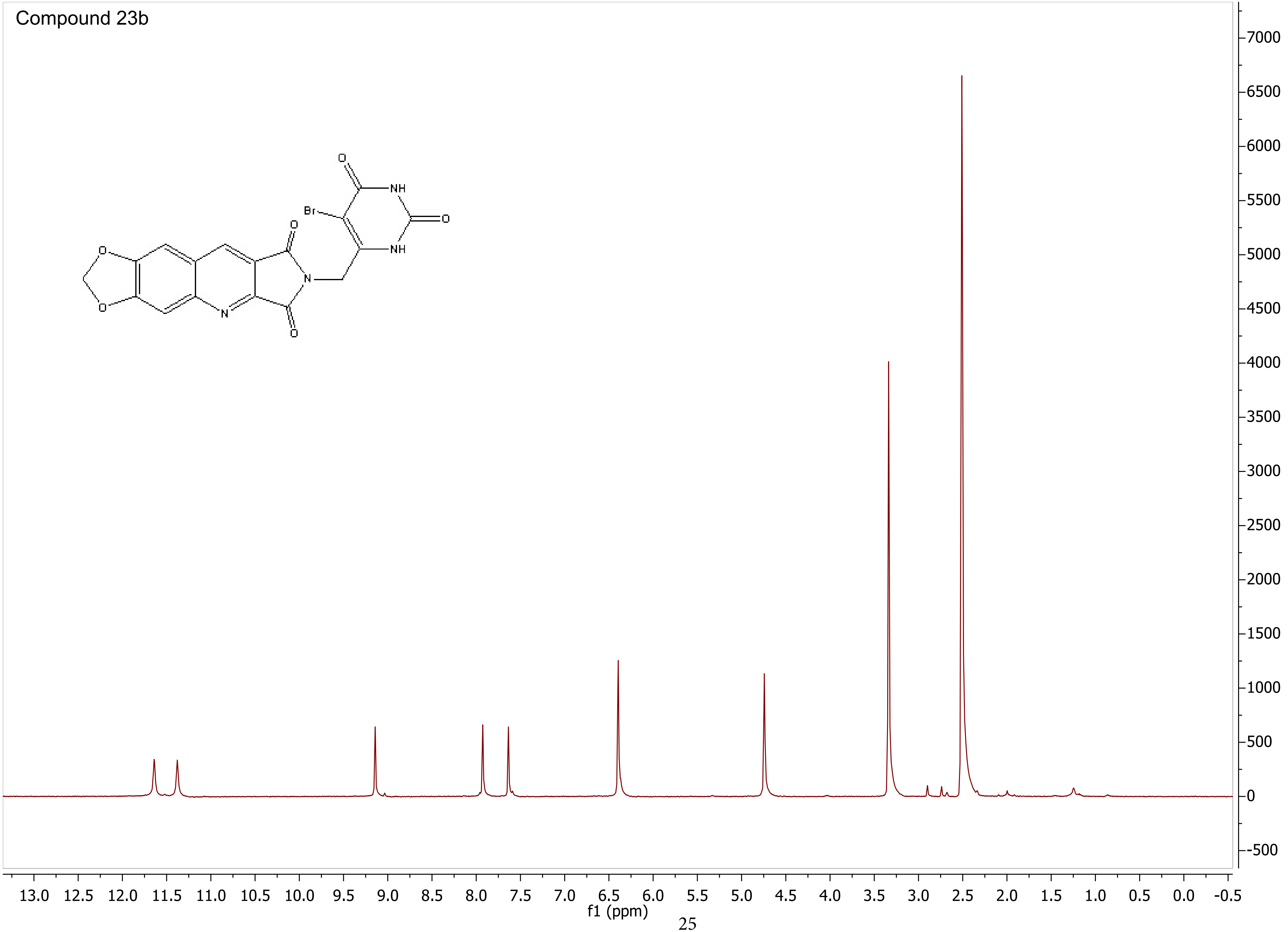
Compound 23b
O
NH
Br
O
O
NH
O
N
O
O
N
O
7000
6500
6000
5500
5000
4500
4000
3500
3000
2500
2000
1500
1000
500
0
-500
13.0 12.5 12.0 11.5 11.0 10.5 10.0 9.5 9.0 8.5 8.0 7.5 7.0 6.5 6.0 5.5 5.0 4.5 4.0 3.5 3.0 2.5 2.0 1.5 1.0 0.5 0.0 -0.5
f1 (ppm)

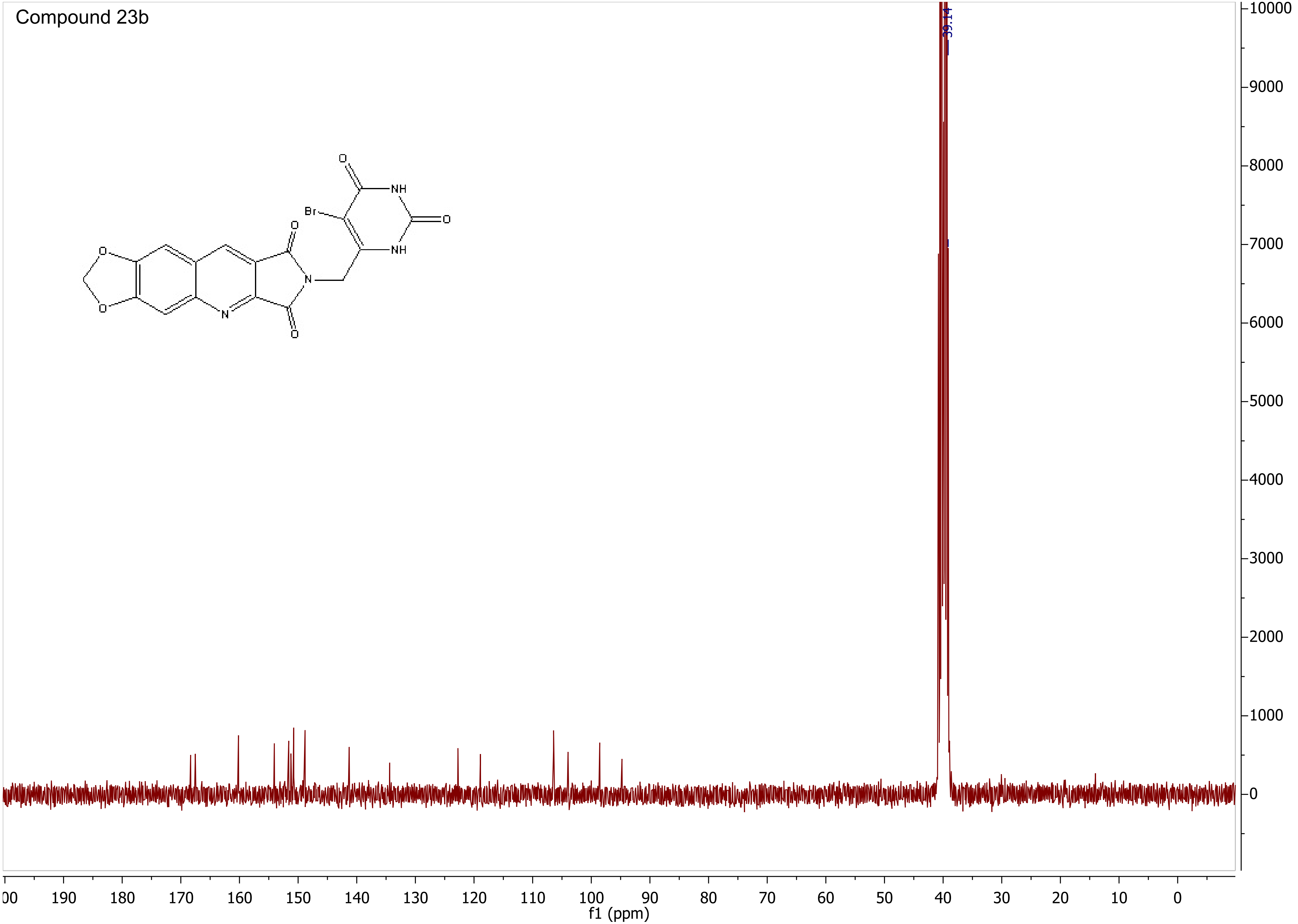
Compound 23b
39.14
f1 (ppm)
Br
NH
O
N

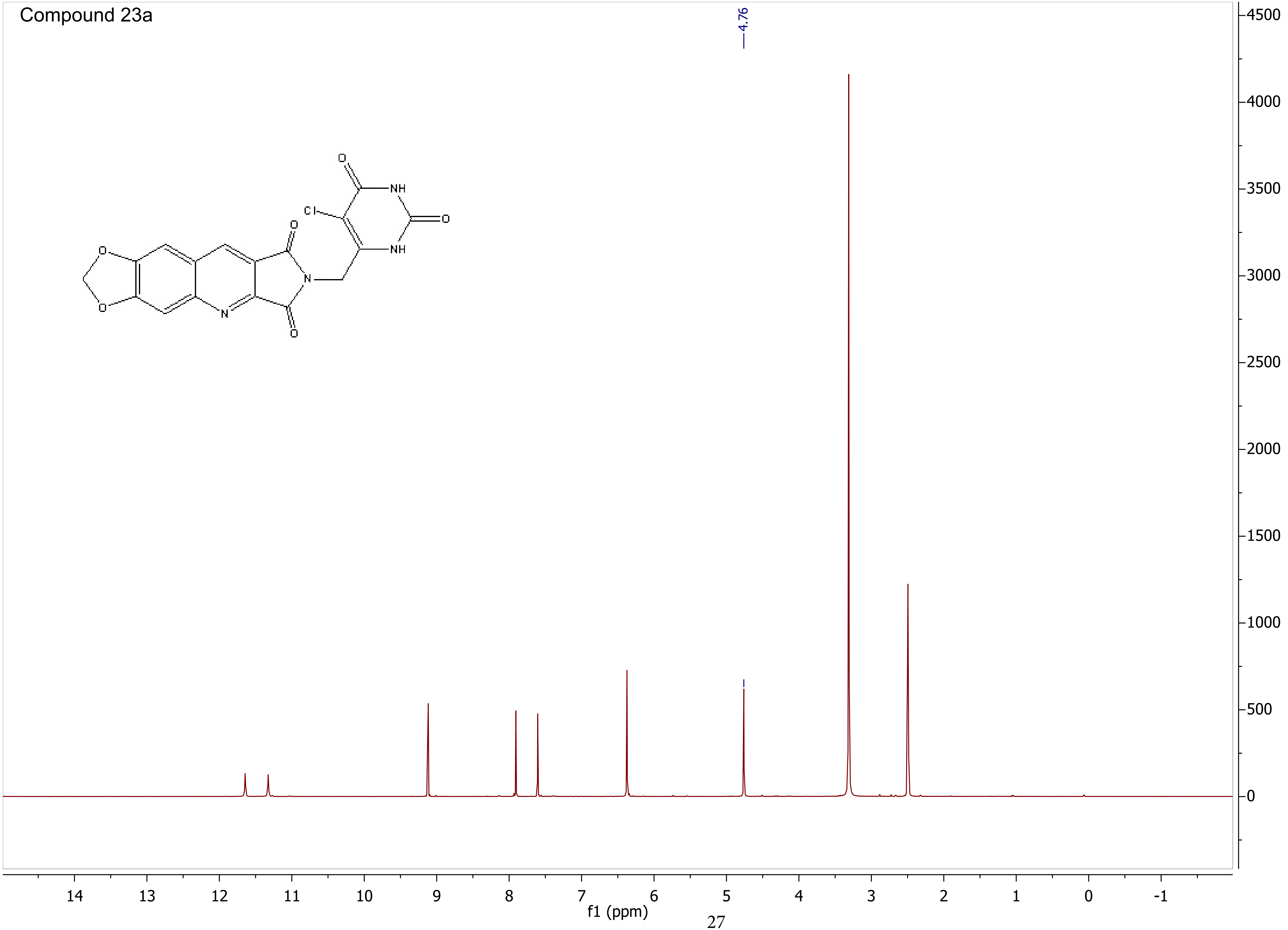
Compound 23a
4.76
O
NH
Cl
O
O
NH
O
N
O
N
O
f1 (ppm)
14 13 12 11 10 9 8 7 6 5 4 3 2 1 0 -1
4500 4000 3500 3000 2500 2000 1500 1000 500 0

Compound 23a

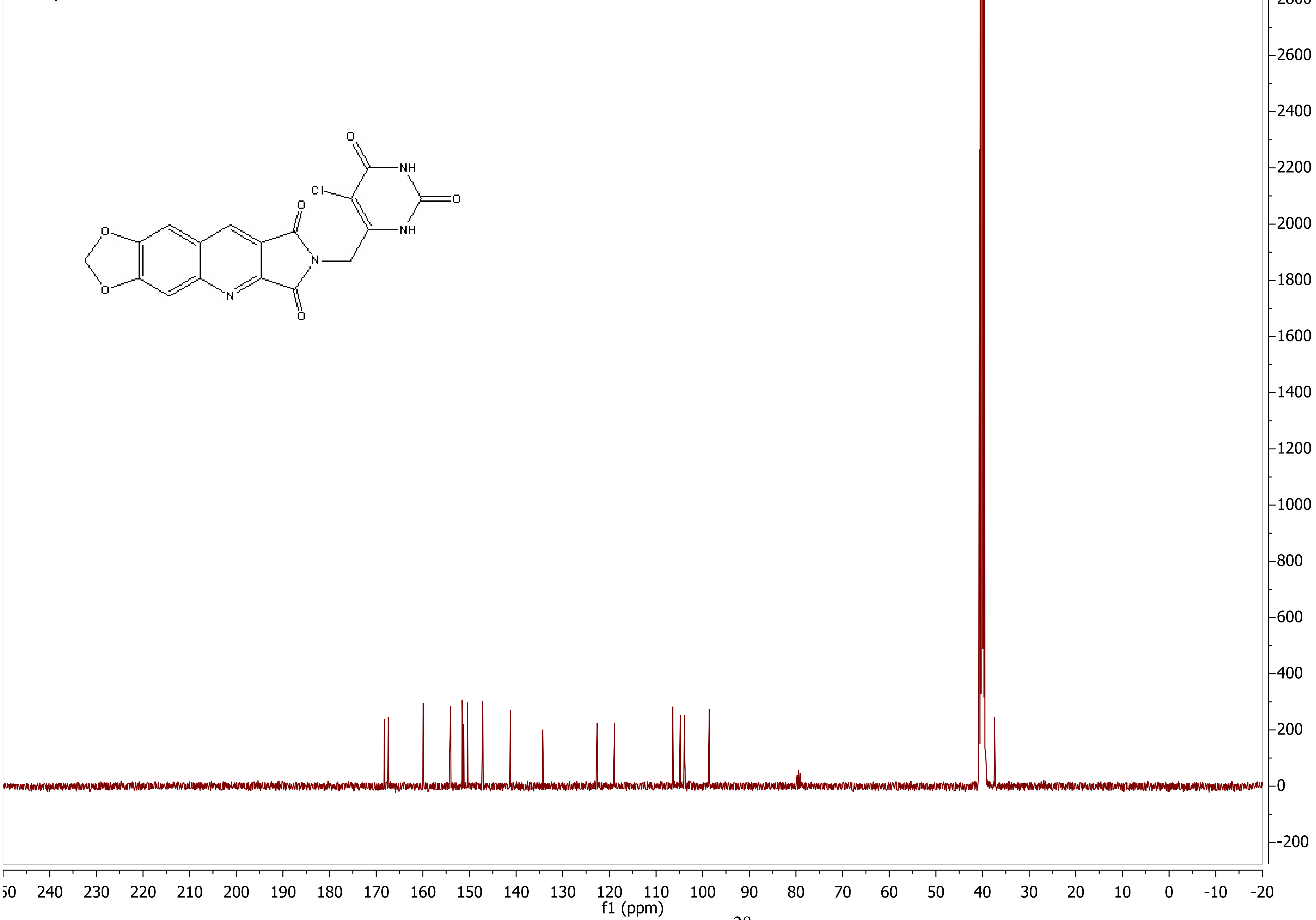

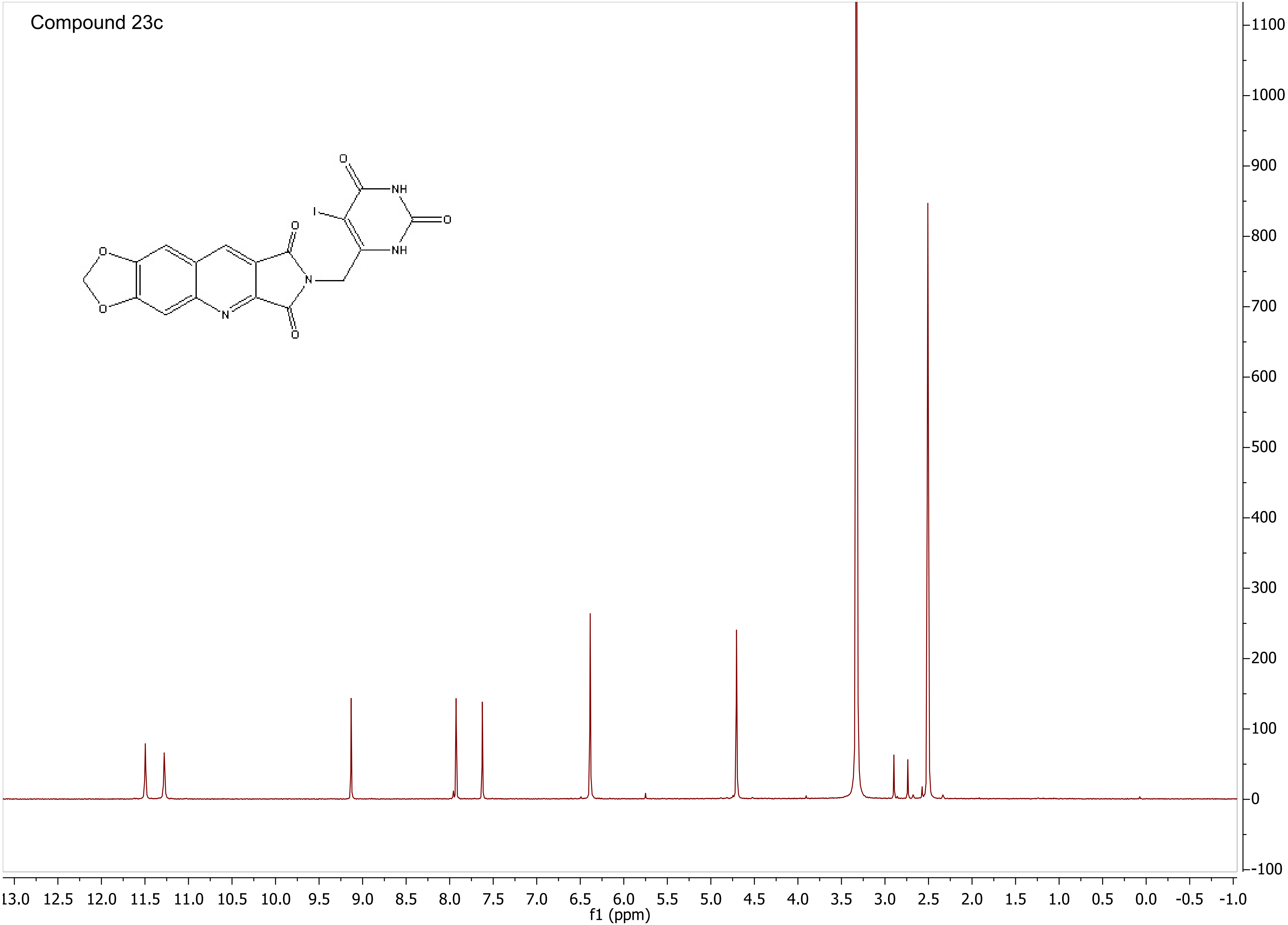
Compound 23c
O
NH
I
O
O
NH
O
N
O
N
O
f1 (ppm)

Compound 23c

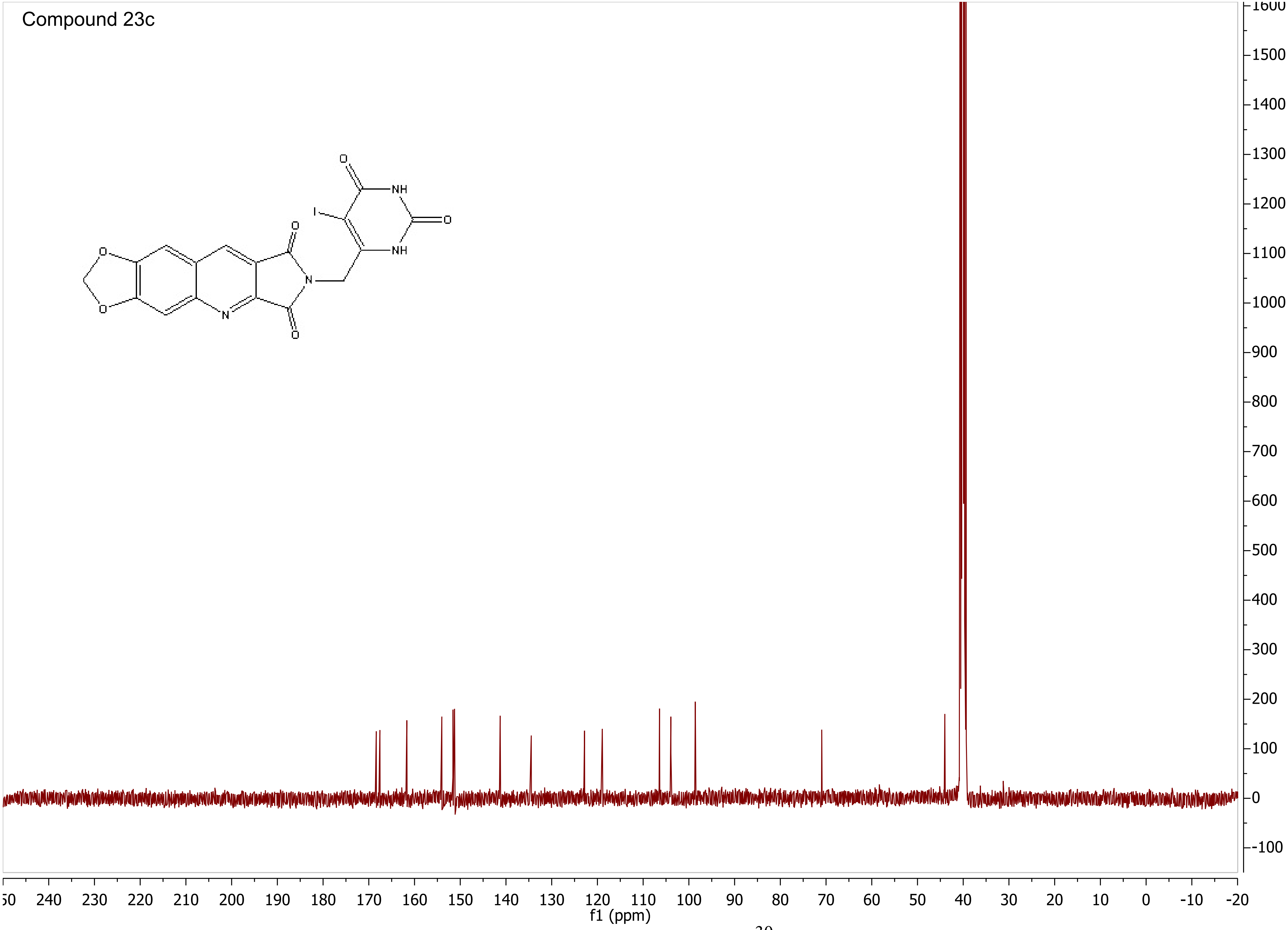

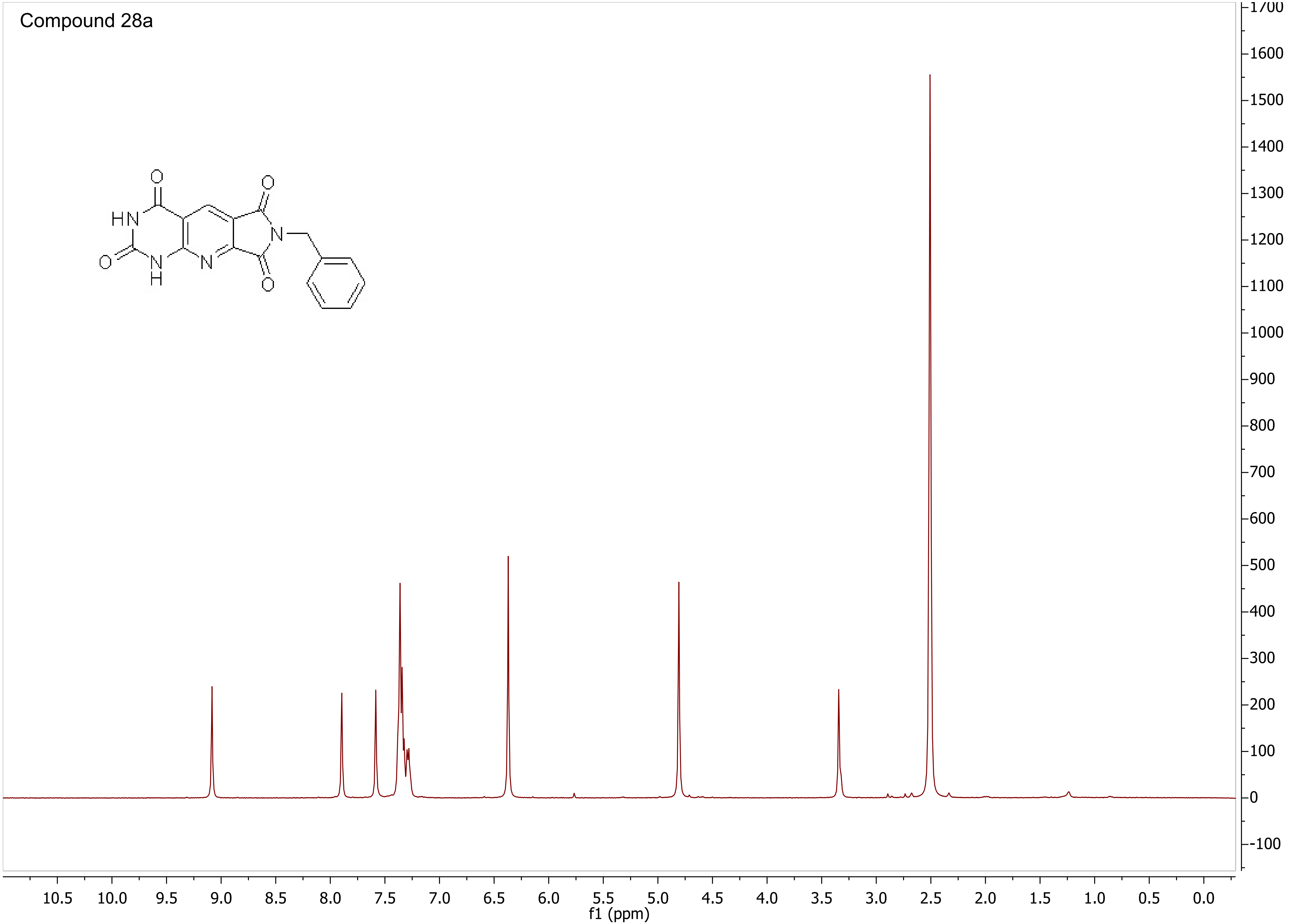
Compound 28a
f1 (ppm)
10.5 10.0 9.5 9.0 8.5 8.0 7.5 7.0 6.5 6.0 5.5 5.0 4.5 4.0 3.5 3.0 2.5 2.0 1.5 1.0 0.5 0.0
1700 1600 1500 1400 1300 1200 1100 1000 900 800 700 600 500 400 300 200 100 0 -100

Compound 28a

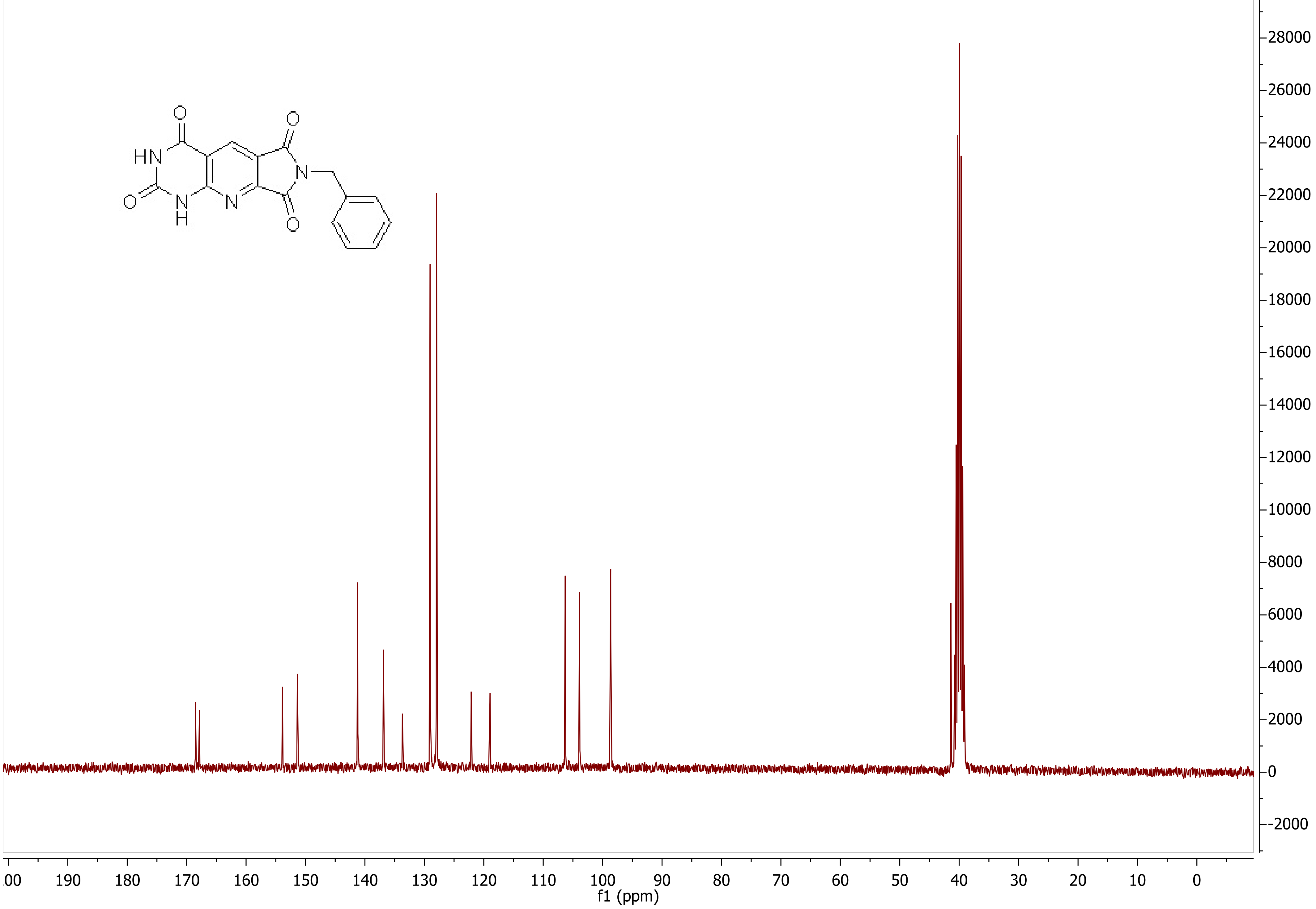

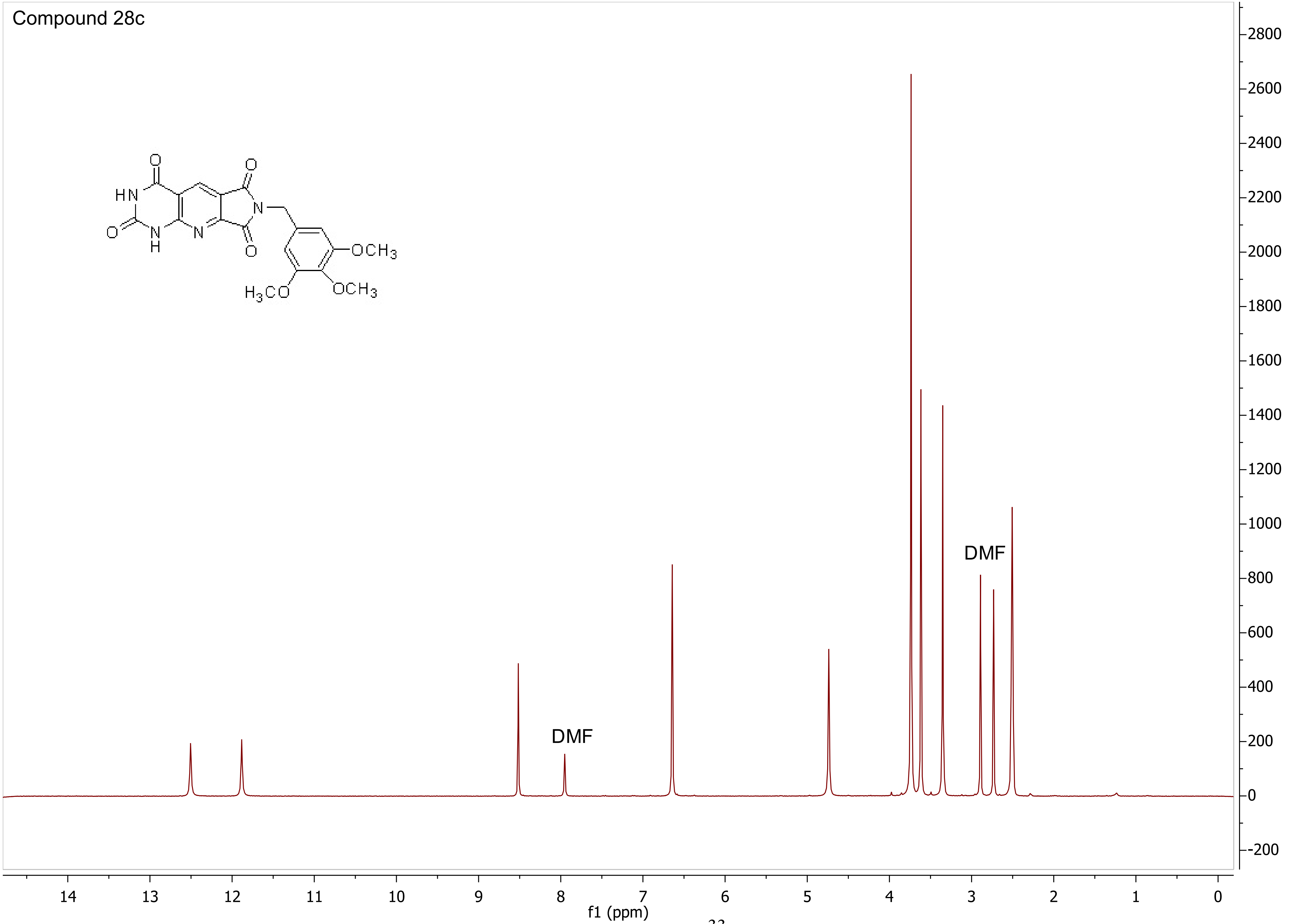
Compound 28c
HN
O
N
H
N
N
O
O
O
OCH3
H3CO
OCH3
DMF
DMF
f1 (ppm)

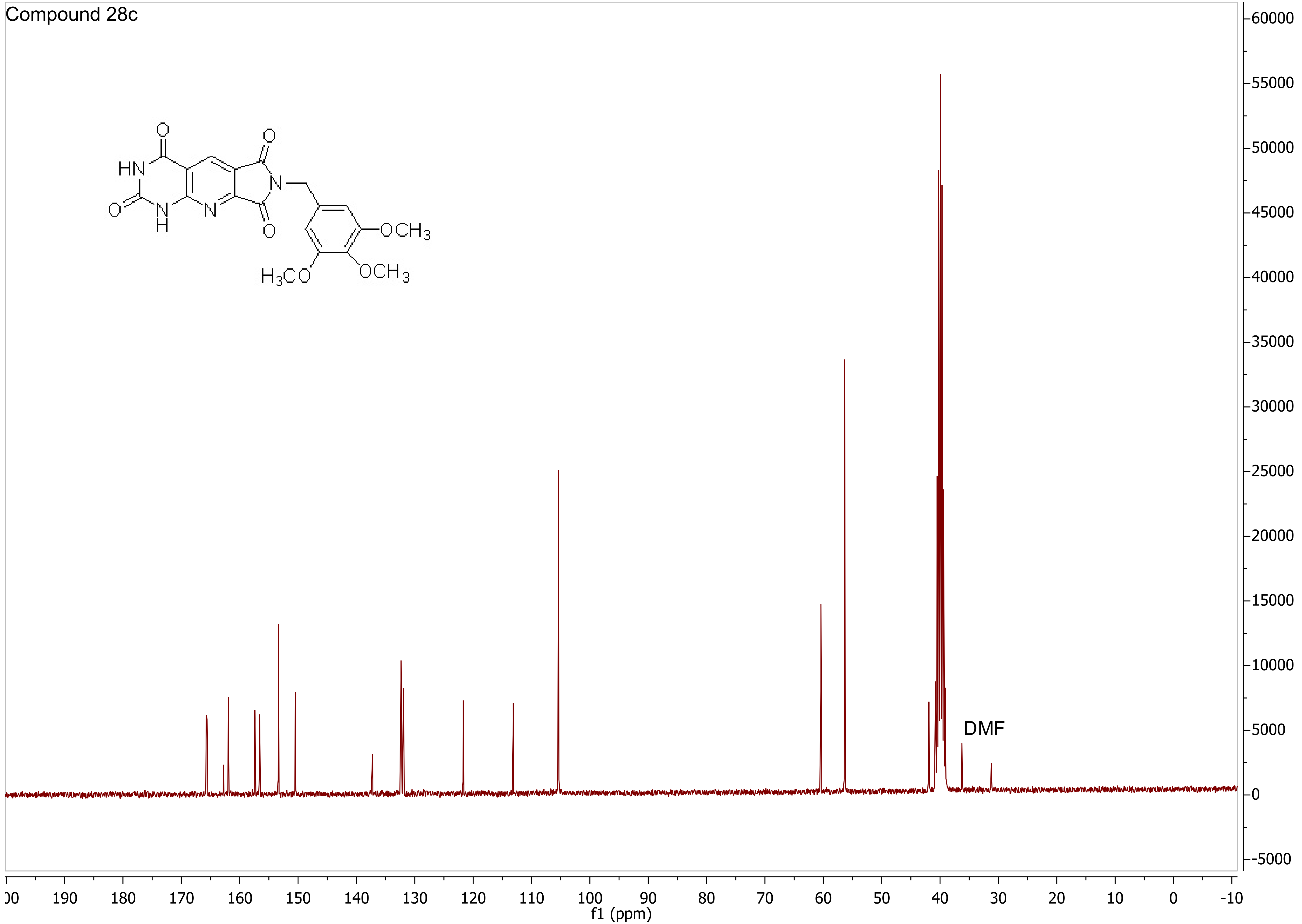
Compound 28c
HN
N
H
N
N
O
O
O
O
OCH3
OCH3
H3CO
DMF
60000
55000
50000
45000
40000
35000
30000
25000
20000
15000
10000
5000
0
-5000
00 190 180 170 160 150 140 130 120 110 100 90 80 70 60 50 40 30 20 10 0 -10
f1 (ppm)

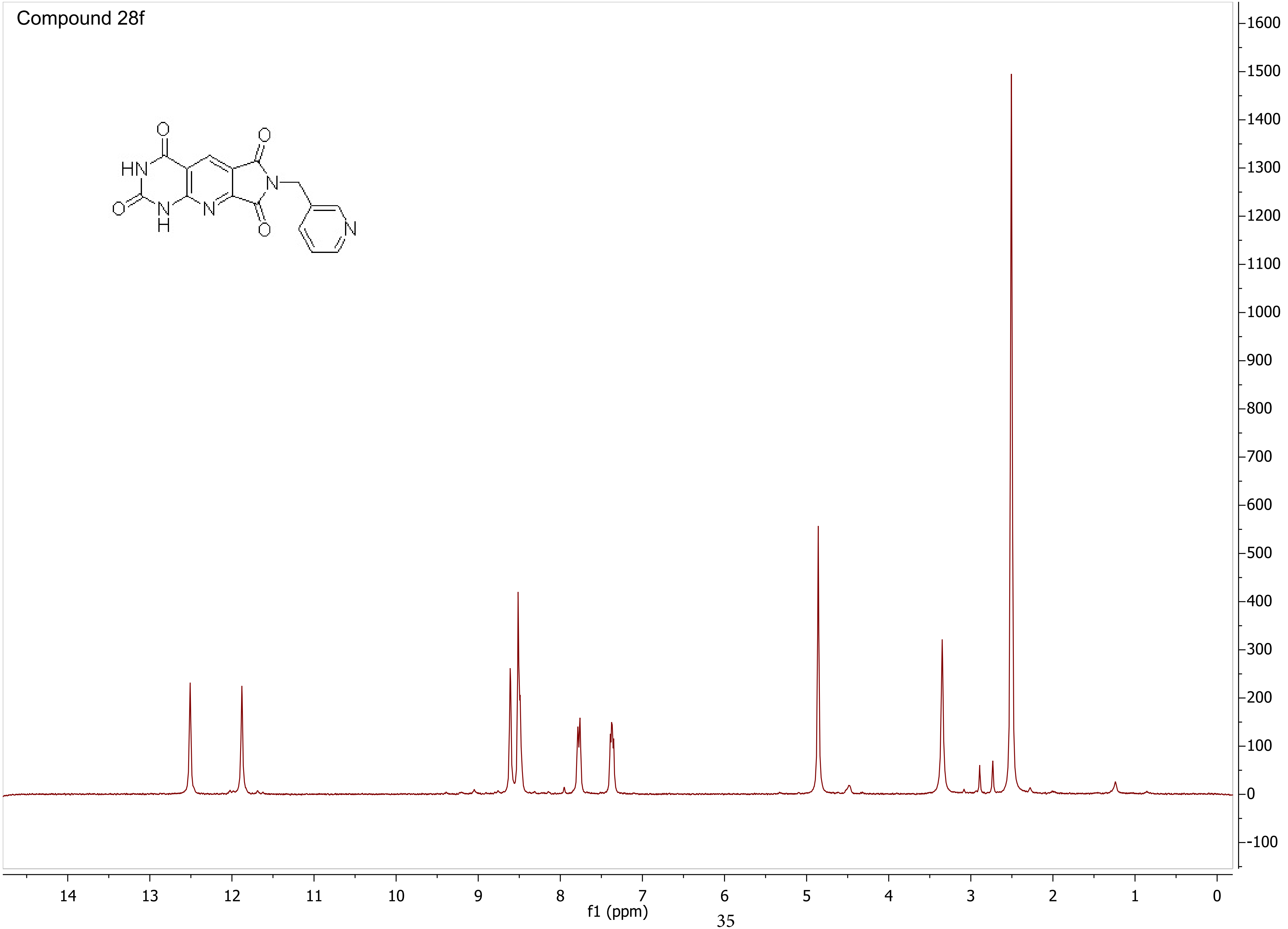
Compound 28f
HN
N
H
O
N
f1 (ppm)
14 13 12 11 10 9 8 7 6 5 4 3 2 1 0
1600 1500 1400 1300 1200 1100 1000 900 800 700 600 500 400 300 200 100 0 -100

Compound 28f

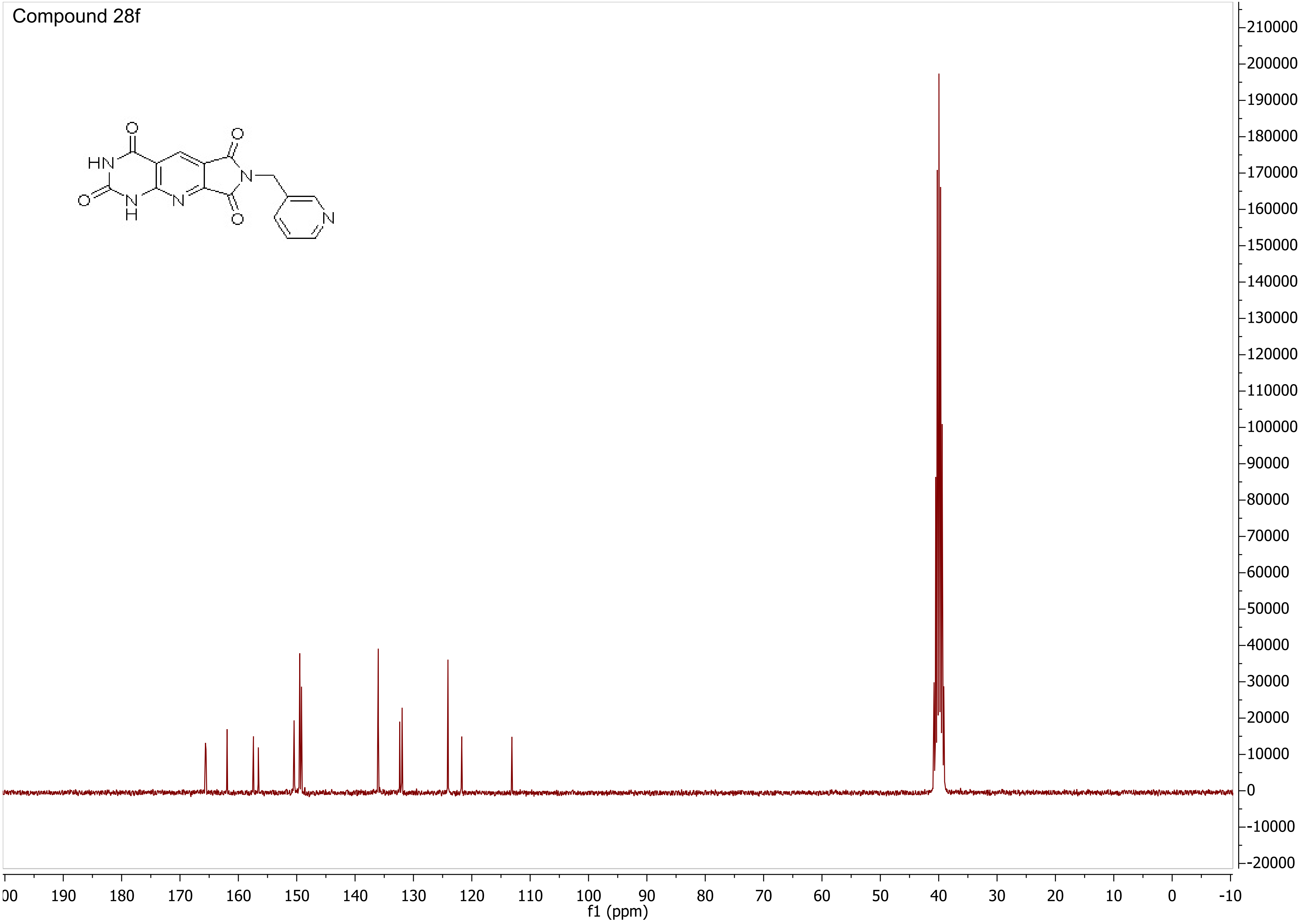

Compound 28e

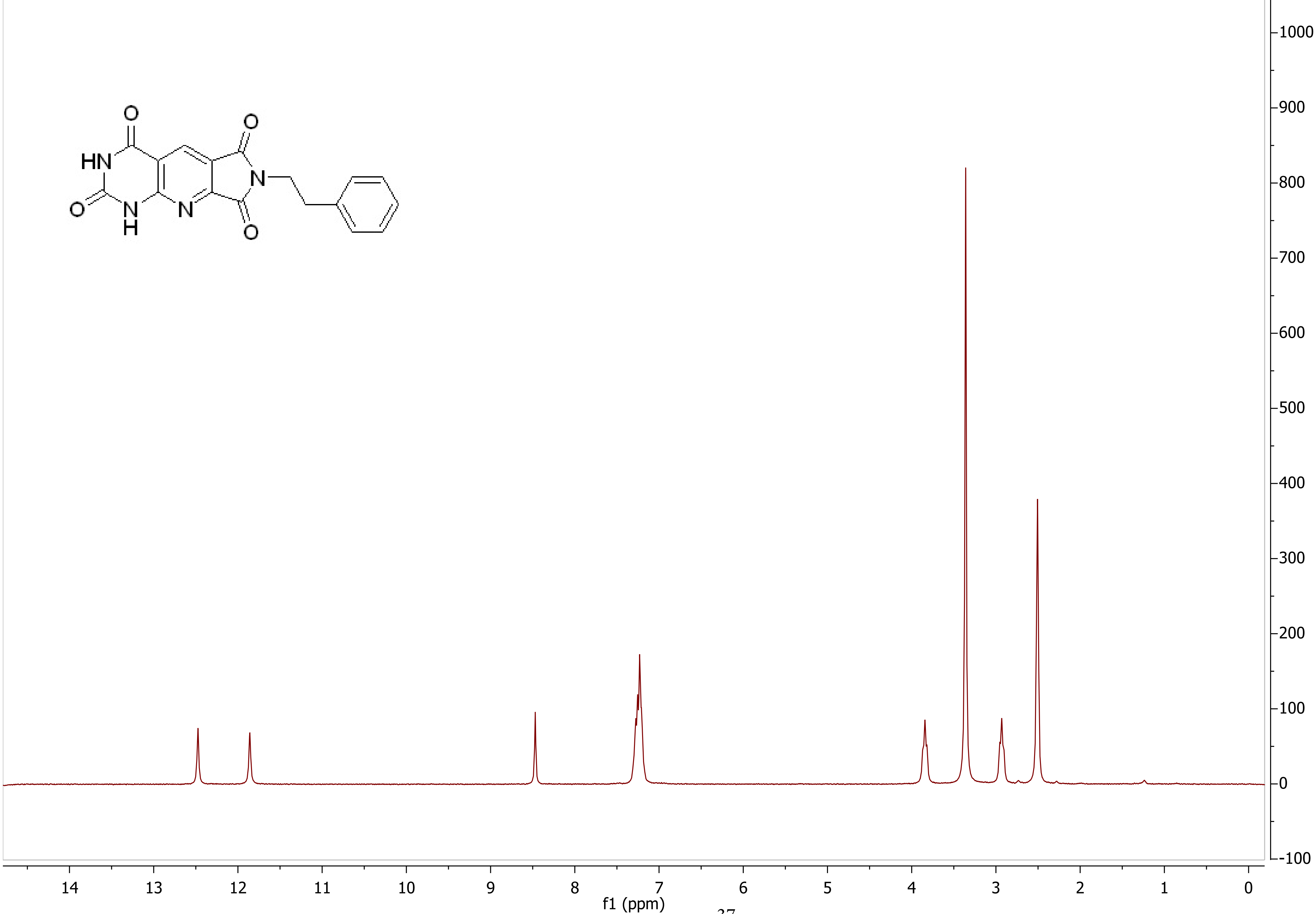

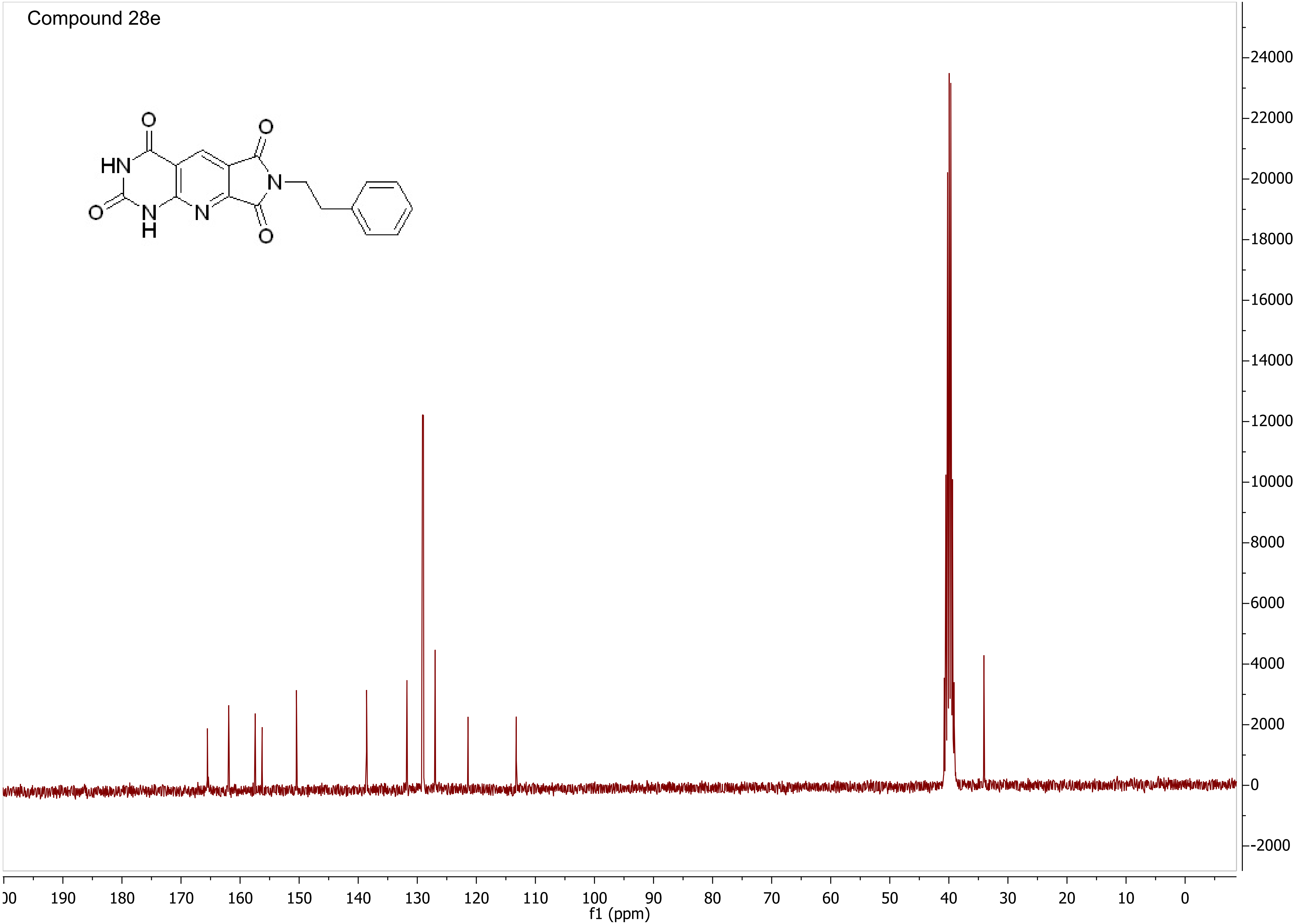
Compound 28e
f1 (ppm)

Compound 28h

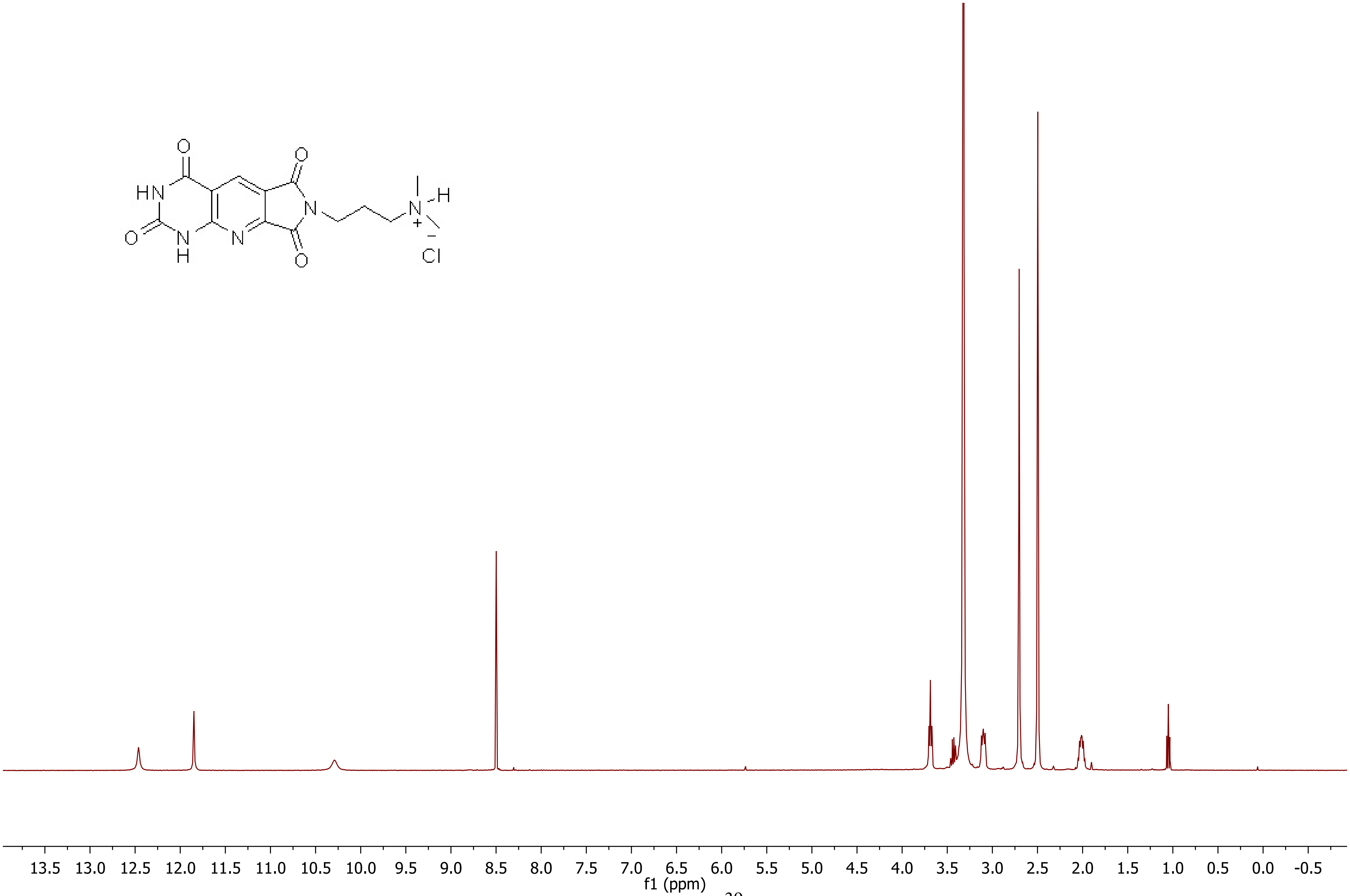

Compound 28h

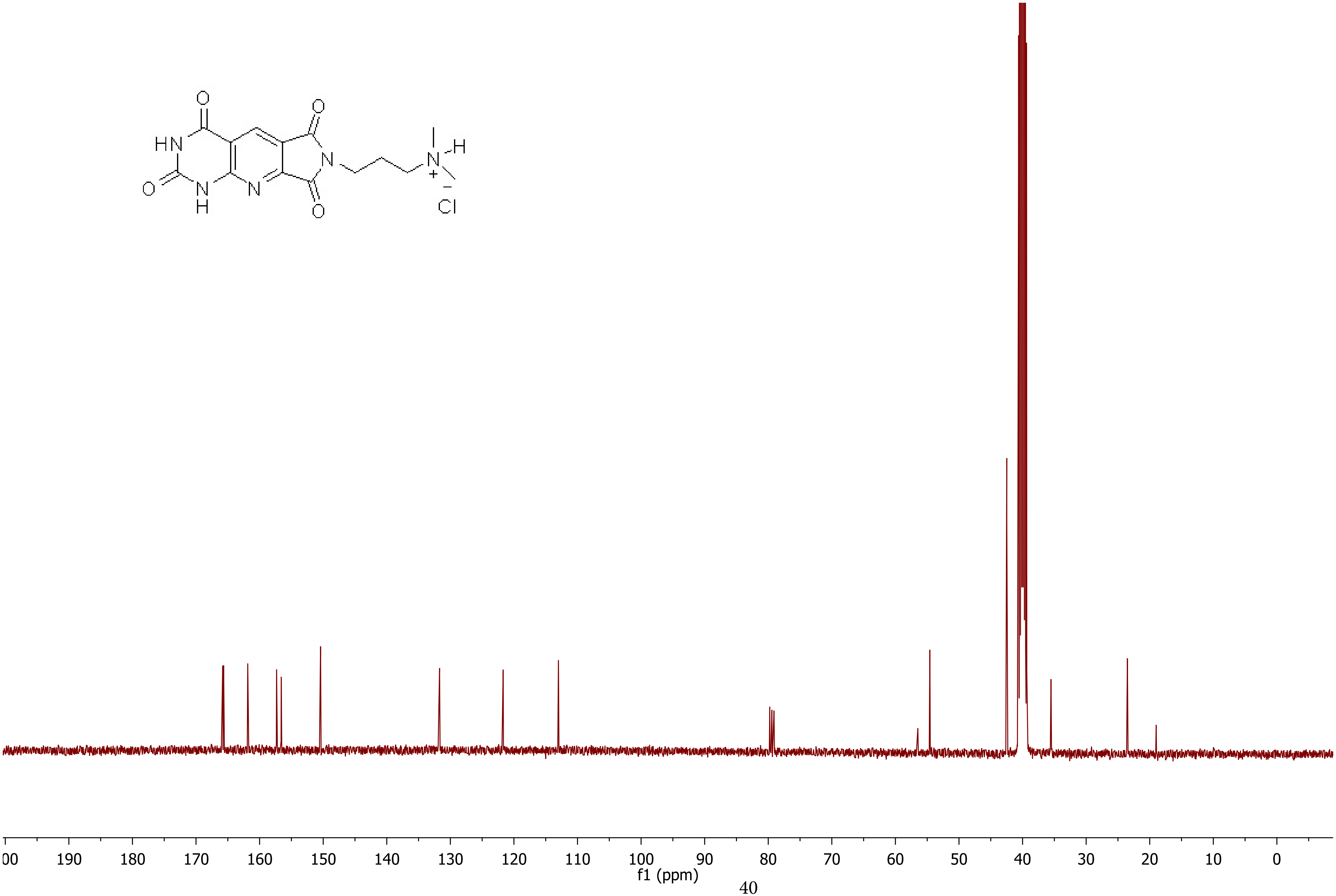

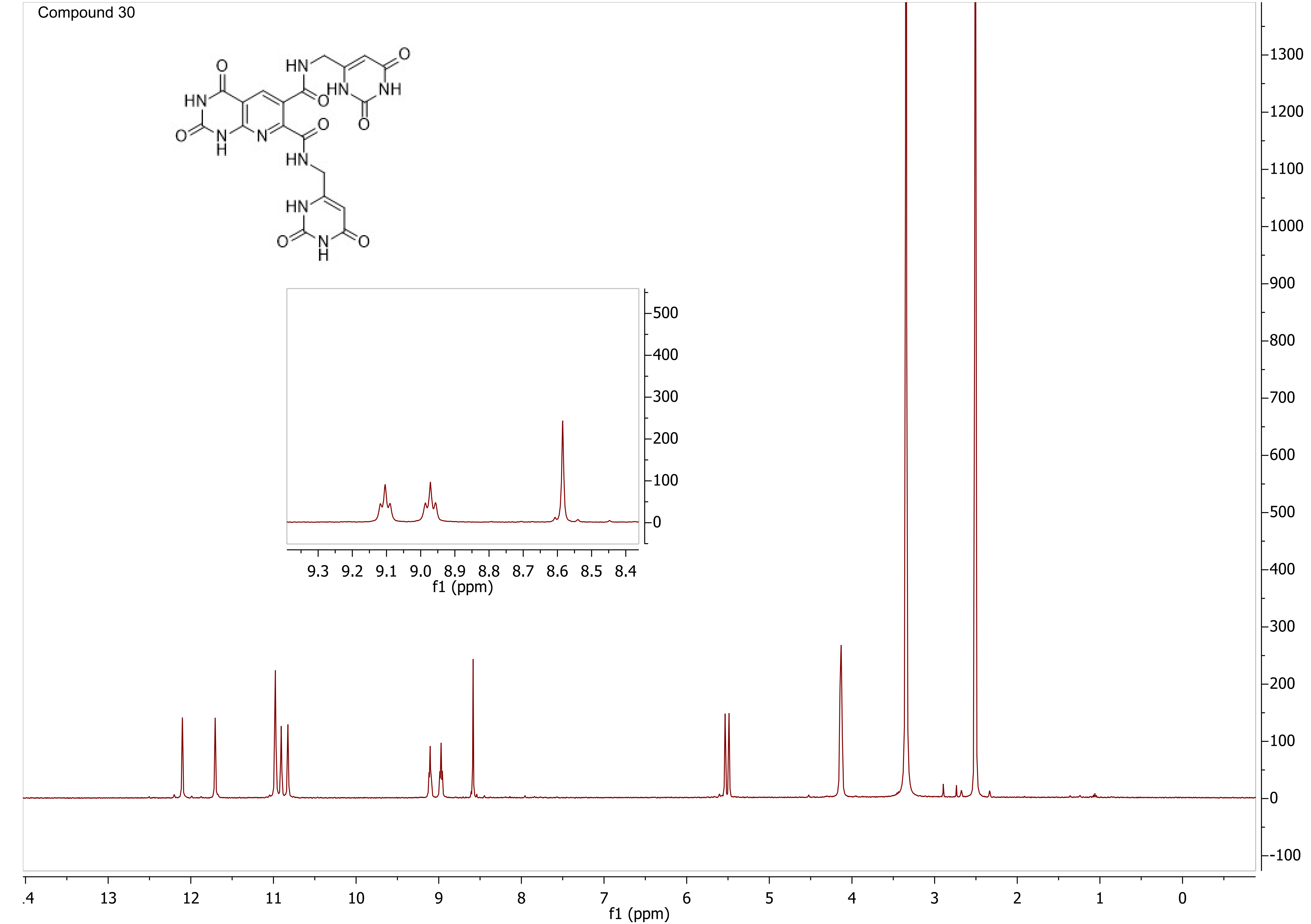
Compound 30
f1 (ppm)
f1 (ppm)

Compound 30

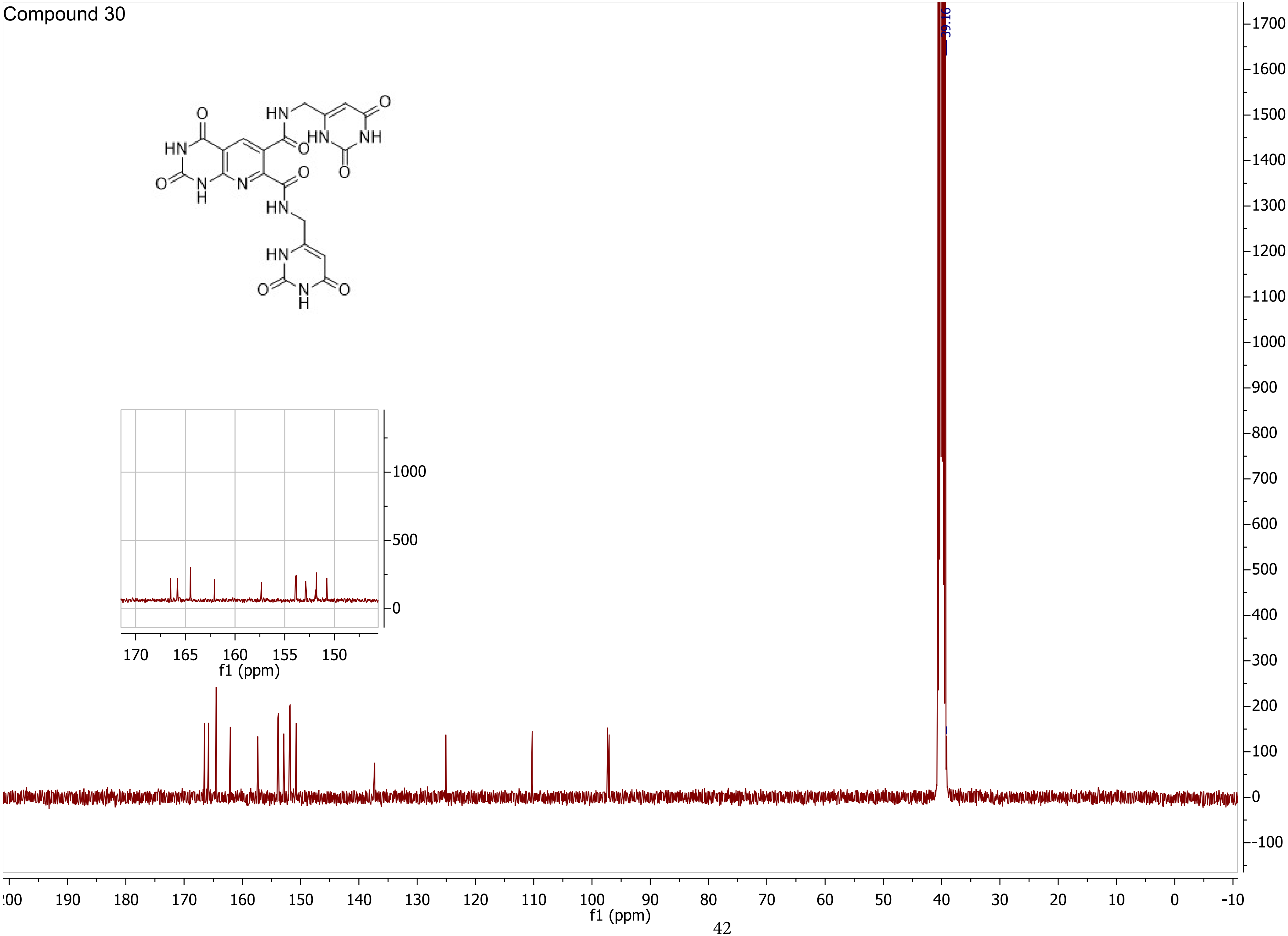

39.16
170
165
160
155
150
f1 (ppm)
1000
500
0
1700
1600
1500
1400
1300
1200
1100
1000
900
800
700
600
500
400
300
200
100
0
-100
200
190
180
170
160
150
140
130
120
110
100
90
80
70
60
50
40
30
20
10
0
-10
f1 (ppm)
